# Nucleophile promoted gold redox catalysis with diazonium salts: C–Br, C–S and C–P bond formation through catalytic Sandmeyer coupling†
†Electronic supplementary information (ESI) available. See DOI: 10.1039/c6sc01742h


**DOI:** 10.1039/c6sc01742h

**Published:** 2016-06-10

**Authors:** Haihui Peng, Rong Cai, Chang Xu, Hao Chen, Xiaodong Shi

**Affiliations:** a Department of Chemistry , University of South Florida , Tampa , FL 33620 , USA . Email: xmshi@usf.edu; b C. Eugene Bennett Department of Chemistry , West Virginia University , Morgantown , WV 26506 , USA; c Center for Intelligent Chemical Instrumentation , Department of Chemistry and Biochemistry , Edison Biotechnology Institute , Ohio University , Athens , OH 45701 , USA

## Abstract

Gold-catalyzed C-heteroatom (C–X) coupling reactions are evaluated without using sacrificial oxidants. Vital to the success of this methodology is the nucleophile-assisted activation of aryldiazonium salts, which could be an effective oxidant for converting Au(i) to Au(iii) even without the addition of an assisting ligand or photocatalyst. By accelerating the reaction kinetics to outcompete C–C homo-coupling or diazonium dediazoniation, gold-catalyzed Sandmeyer reactions were achieved with different nucleophiles, forming C–Br, C–S and C–P bonds in high yields and selectivities.

## Abstract 2

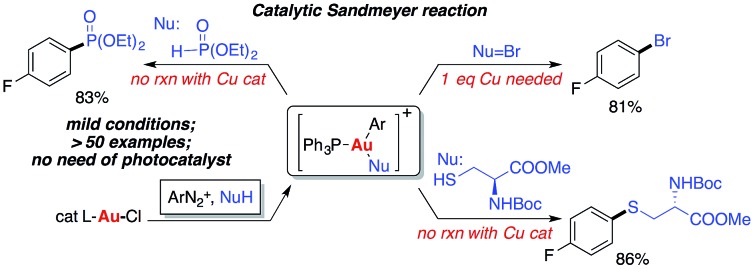
Gold-catalyzed C-heteroatom (C–X) coupling reactions are evaluated without using sacrificial oxidants.

## 

Homogeneous gold catalysis has been well developed for the activation of C–C multiple bond in the past two decades.^1^ However, compared with Pd(0), a d^10^ isoelectronic counterpart, traditional redox chemistry with Au(i) is relatively rare due to the higher oxidation potential between Au(i) and Au(iii).^2^ To maximize the potential of gold catalysis, extensive effort has been put into the development of this new branch of gold chemistry.^3^ Typically, strong external oxidants, such as Selectfluor and hypervalent iodine, are usually required to access catalytically active Au(iii) intermediates. The need for strong oxidants made gold redox chemistry less attractive, especially for the synthesis of complex molecules. One of the most significant improvements in gold redox chemistry is dual photoredox and gold catalysis, first reported by Glorius' and Toste's groups (Scheme 1).^4^ In their studies, a photocatalyst was used to promote gold redox oxidation under mild conditions. More recently, Hashmi and coworkers further extended this chemistry to photosensitizer-free conditions, achieving alkyne 1,2-difunctionalization with only a gold catalyst under visible-light.^5^ In this study, a gold(iii) intermediate was successfully isolated, which supported a gold redox catalytic mechanism under photo-initiated conditions. Herein, we report the investigation of nucleophile promoted diazonium activation for promoting gold(i) oxidation. Through mechanistic investigation using NMR and electrospray ionization mass spectrometry (ESI-MS), nucleophile was identified as a critical factor in promoting this gold redox chemistry. In addition, through suppressing the undesired C–C homocoupling (*via trans*-metallation and reductive elimination), catalytic Sandmeyer coupling was achieved and C–X bonds (X = Br, S and P) were formed in good to excellent yields.^6^ Under these new conditions, no strong oxidants or photocatalysts are required to promote gold oxidation, which will potentially open new avenues for future developments in gold redox chemistry.

**Scheme 1:**
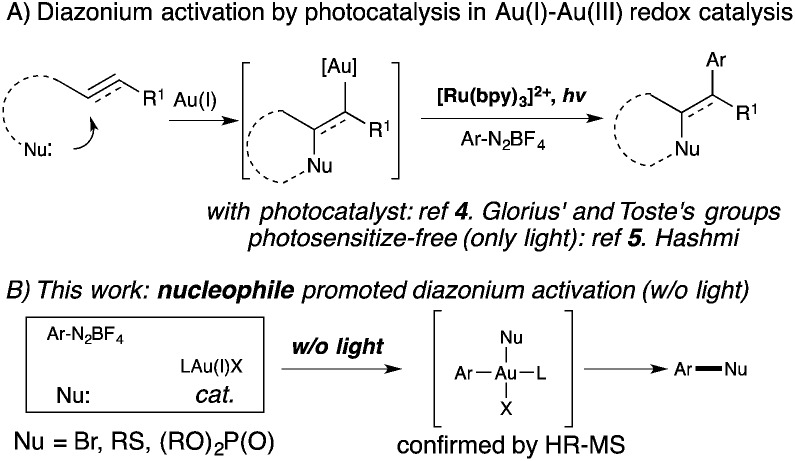
Gold redox catalysis.

The high-oxidation potential between Au(i) and Au(iii) has been a major concern that has hindered the development of gold redox catalysis for a long time. Thus, achieving gold oxidation under mild conditions is crucial. Our group recently reported the gold catalyzed C–C coupling reaction between alkynes and aryldiazonium salts.^7^ Based on that study, diazonium activation can be achieved with the help of a 2,2′-bipyridine (bpy) ligand even without light. Although visible-light conditions are extremely mild and readily accessible, understanding the function of bpy ligand will certainly help the elucidation of reaction mechanism, which will be beneficial for the further development of gold redox chemistry under mild conditions.

Notably, Shin and coworkers have reported the detection of an Au(iii) intermediate (using XPS) through mixing PPh_3_AuCl and an aryl diazonium salt in MeOH/CH_3_CN (20 : 1) at 60 °C.^8^ To explore the role of bpy ligands, we monitored the reaction of diazonium salt **1a** (*p*-F-C_6_H_4_N_2_BF_4_) and PPh_3_AuCl using ^31^P NMR. Interestingly, when mixing **1a** and PPh_3_AuCl in CH_3_CN, no reaction was observed, even under long exposure to light at 50 °C (Fig. 1a). In contrast, with MeOH/CH_3_CN (9 : 1) as the solvent, phosphonium salt **2a** was detected (22.5 ppm, Fig. 1b), though in a low yield (23% based on NMR). Interestingly, with the addition of 1.0 equiv. of bpy, **2a** was formed at a much faster rate and PPh_3_AuCl was totally consumed within an hour (Fig. 1c and d).^9^

**Fig. 1:**
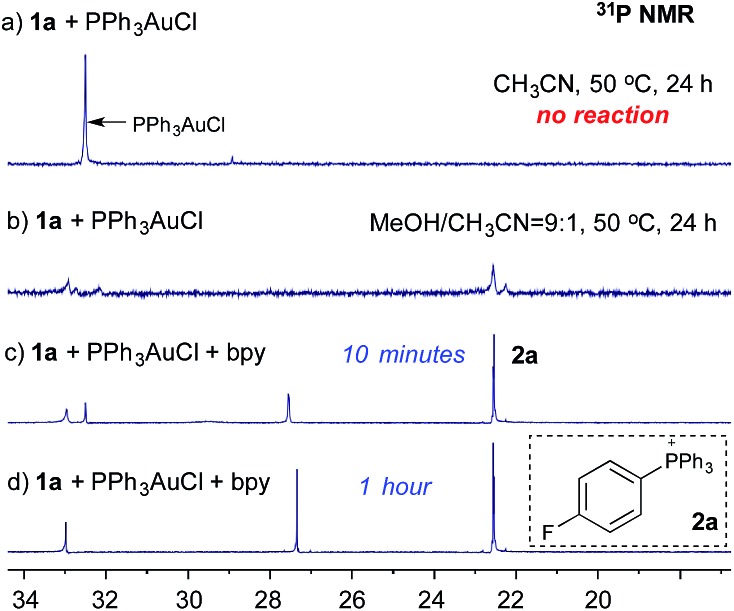
Monitoring the reaction of **1a** and PPh_3_AuCl with ^31^P NMR.

The formation of phosphonium salt **2a** strongly suggested that an Au(iii) intermediate is formed during the reaction of PPh_3_AuCl and diazonium salts with assistance from bpy. Thus, it is likely that the combination of bpy and a diazonium salt is the actual oxidant for the oxidation of PPh_3_AuCl. Notably, it has been reported in the literature that pyridine can promote diazonium activation through nucleophilic addition.^10^ Thus, a similar function of bpy is expected as a nucleophile in assisting diazonium activation, which accounts for the observed gold oxidation even without photoinitiation. ESI-MS was used to explore the reaction intermediates. As expected, treating PPh_3_AuCl/ArN_2_^+^/bpy (*m*/*z* = 745.12) gave the clear formation of a [PPh_3_Au(Ar)bpy]^+^ cation which was also supported by further collision induced dissociation (CID) studies (MS/MS, see details in the ESI†). This result confirmed the gold oxidation by diazonium salts with the assistance of a bpy ligand. Encouraged by this discovery of nucleophilic ligand assisted diazonium activation, we wondered whether it was possible to further extend this gold redox chemistry into challenging C–X bond coupling. Our hypothesis was to explore appropriate anionic nucleophiles to achieve both diazonium activation (for gold activation) and coupling (through reductive elimination) under these mild gold redox conditions with no need for additional photosensitizers (Scheme 2).^11^

**Scheme 2:**
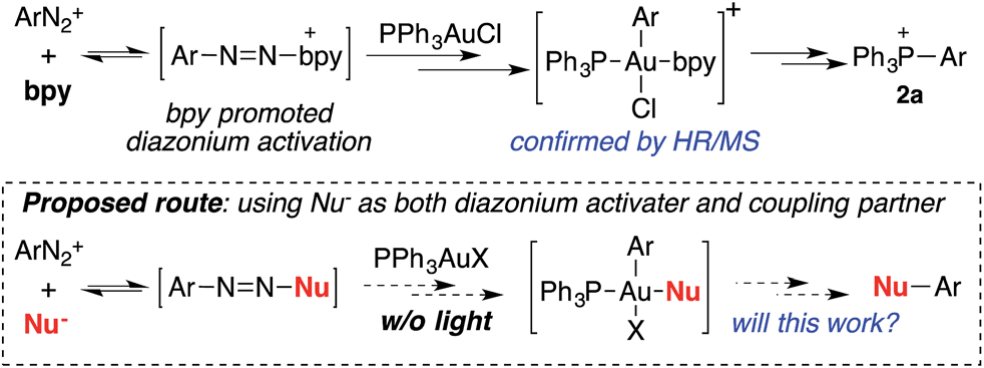
Proposed Ar–Nu coupling with gold-redox catalysis.

It is well known that the conversion of ArN_2_^+^ to ArCl or ArBr can be achieved through standard Sandmeyer conditions using a stoichiometric amount of CuX.^12^ Successful examples of catalytic Sandmeyer reactions are rare. More importantly, CuX could not promote effective C–S and C–P bond formation through a coupling mechanism. Compared with C–C bond coupling, the formation of a C–X bond from a coupling reaction is thermodynamically less favorable. Thus, there were only a few successful examples reported where this important transformation was achieved catalytically.^13^ Therefore, the proposed gold-catalyzed coupling is attractive not only due to the mechanistic novelty (no need for a strong oxidant or photo-activation), but also because of its potential synthetic applications (the formation of challenging C–X bonds under catalytic conditions).

In the NMR studies shown in Fig. 1, only a trace amount of aryl chloride was observed, although a stoichiometric amount of PPh_3_AuCl was used. One possibility is that the reductive elimination of Ar–Cl from Au(iii) is unfavorable under gold redox conditions. In fact, Toste group recently confirmed the reductive elimination rate as I > Br > Cl through careful evaluation of different Au(iii)–X bond dissociation energies.^14^ To explore the proposed catalytic C–X bond formation using gold redox chemistry, we started our investigation with the C–Br bond. To our great satisfaction, an excellent yield of aryl bromide **5a** was achieved using the gold catalyst under mild conditions (3% PPh_3_AuCl, 81% in 5 h). Results from some alternative conditions are shown in Table 1.

**Table 1:** Exploring the reaction conditions^*a*^ ^*b*^

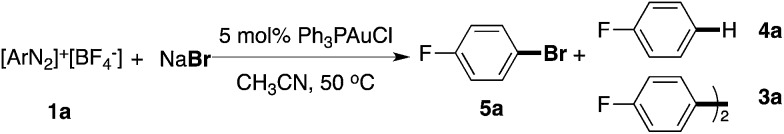						
Entry	Variations from above conditions	Time	Conv. (%)	**5a** (%)	**3a** (%)	**4a** (%)
1	None	5 h	100	83	7	<5
2	Blue LED, No Ph_3_PAuCl	12 h	50	<10	Trace	33
3	LiBr instead of NaBr	12 h	100	78	8	<5
4	Acetone instead of ACN	5 h	100	11	37	<5
5	Ph_3_PAuNTf_2_ instead of Ph_3_PAuCl	5 h	100	68	10	7
6	Ph_3_PAuNTf_2_ and 20 mol% bpy	12 h	100	63	8	15
7	3 mol% Ph_3_PAuCl	5 h	100	81	7	<5
8	1 mol% Ph_3_PAuCl	5 h	100	63	13	9
9	No light (darkness)	5 h	100	76	8	<5

^*a*^Reaction conditions: **1** (0.1 mmol), NaBr (0.4 mmol), cat. Au (5 mol%) in acetonitrile (ACN), 50 °C.

^*b*^ ^19^F NMR yield with benzotrifluoride as the internal standard.

Firstly, the bpy ligand is not required in this reaction, which suggests that Br^–^ could act as an activation factor for the diazonium salts. In fact, reacting a diazonium salt with I^–^ gave the formation of aryl iodide even without any catalyst.^15^ Less than 10% Ar–Br was observed without the gold catalyst (entry 2). Switching the solvent to acetone gave a significantly increased yield of the homo-coupling product **3a**, which suggested either a different reductive elimination reaction rate (relative to transmetallation) or an alternative radical reaction path. Lowering the catalyst loading to 1% led to a reduced yield of **5a** (entry 8, 63%) due to the increased aryl homo-coupling and diazonium decomposition (formation of ArH, **4a**). The cationic gold(i) catalyst PPh_3_AuNTf_2_ also promoted the reaction, though with lower yields (entries 5 and 6), which is similar to the performance of the Ph_3_PAuBr catalyst. Importantly, a similar reaction yield was observed while conducting the reaction under dark conditions (entry 9), confirming the reaction as nucleophile-promoted activation rather than light-promoted diazonium decomposition. Overall, to the best of our knowledge, this is the first example of a catalytic Sandmeyer reaction using only a gold catalyst (no photo-activation). With this new optimal condition, various substrates were tested. The reaction substrate scope is shown in Table 2.

**Table 2:** Catalytic Ar–Br cross coupling reaction scope^*a*^ ^*b*^

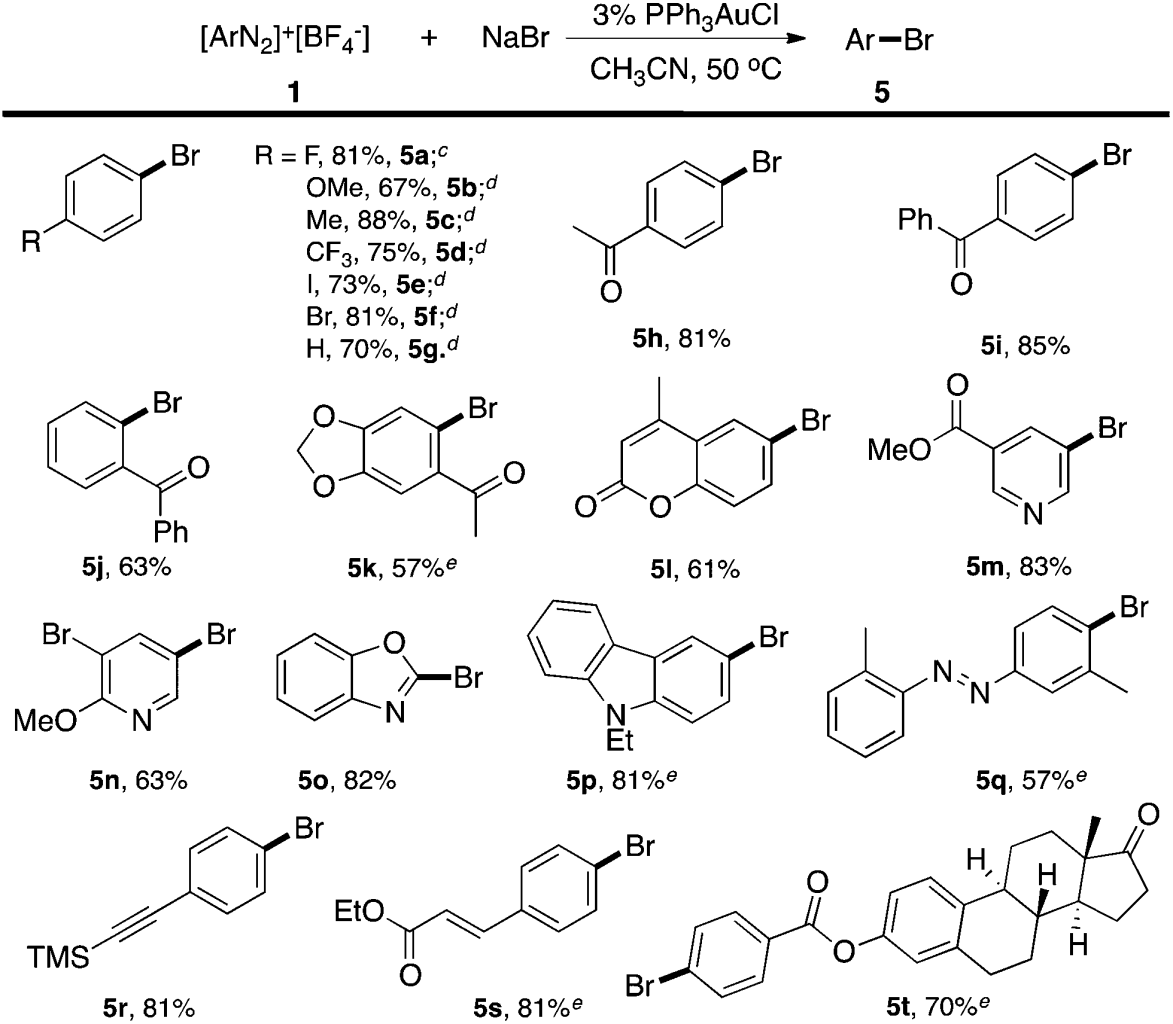

^*a*^Reaction conditions: **1** (0.2 mmol), NaBr (0.8 mmol), PPh_3_AuCl (3 mol%) in acetonitrile (ACN), 50 °C, 5 h.

^*b*^Isolated yield.

^*c*^Determined using ^19^F NMR with benzotrifluoride as the internal standard.

^*d*^Determined using GC-MS with decane as the internal standard.

^*e*^LiBr (1.0 mmol) instead of NaBr.

Excellent substrate compatibility was found. Diazonium salts with EWGs and EDGs all furnished the products in good yields (**5a–5g**). Notably, aryl iodide is also compatible in this catalytic system (**5e**), highlighting the orthogonal reactivity of the Au catalyst over Pd, Cu, and Ni (for which oxidative addition can occur). Carbonyl groups (**5h**, **5i** and **5j**), a benzodioxole (**5k**) and an azobenzene (**5q**) were well tolerated in this reaction. Hetero-aromatic diazonium salts, such as pyridines (**5m** and **5n**) and indoles (**5p**) also worked well in this reaction. Moreover, this reaction proceeded with high efficiency and selectivity for an α,β-unsaturated ester (**5s**) and *p*-acetylide aryl diazonium (**5r**) to give the corresponding products. To further evaluate the synthetic utility and generality of this reaction, we tested a coumarin derivative (**5l**) and estrone derivative (**5t**) under the reaction conditions. The desired products were achieved with good yields, highlighting the good potential of this catalytic system for complex molecular synthesis.

ESI-MS studies were performed to explore the reaction mechanism. As shown in Fig. 2, a bisbromide-aryl-gold(iii) intermediate was observed with MS under the standard reaction conditions. Through collision induced dissociation (CID) studies (MS/MS), the composition of this intermediate was confirmed (see details in the ESI†). This result provided strong evidence for the formation of an Au(iii) intermediate as proposed.

**Fig. 2:**
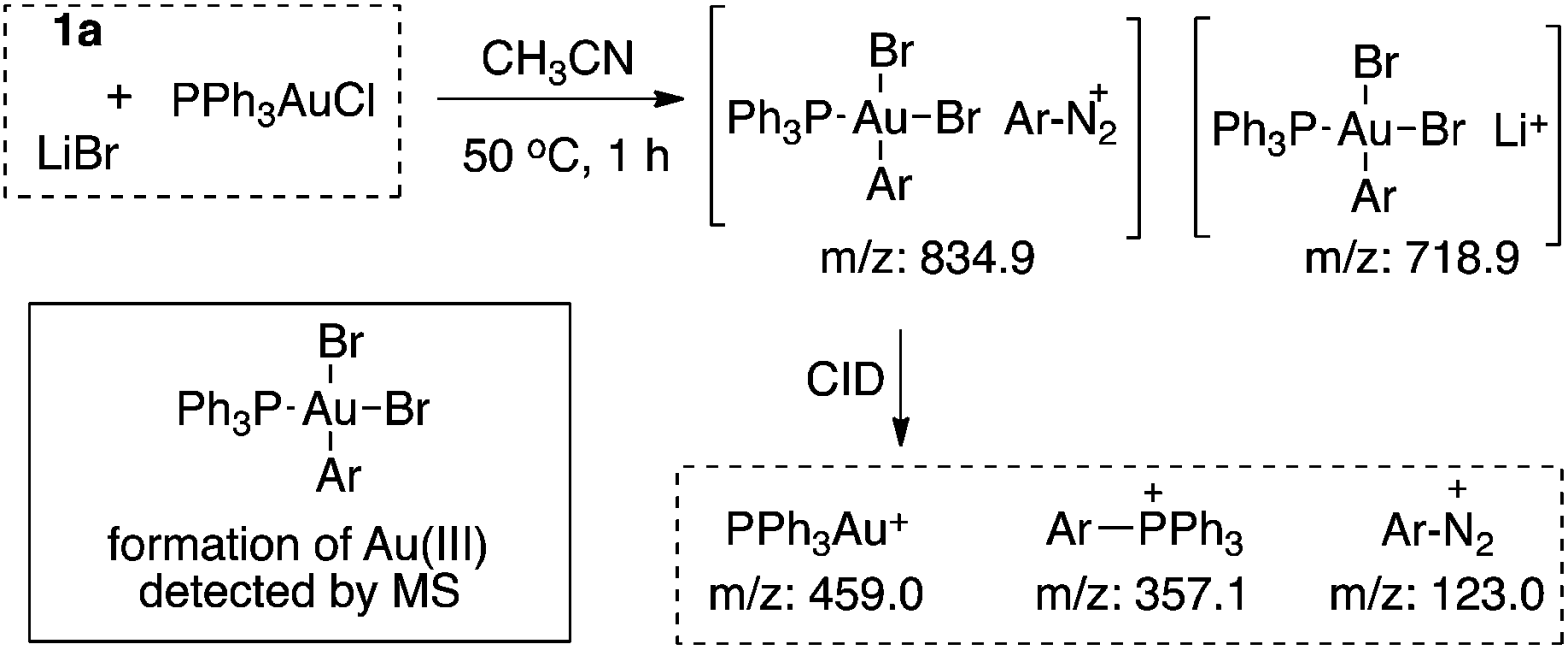
Evidence of an Au(iii) intermediate from ESI-MS.

Encouraged by the success of the gold catalyzed C–Br bond formation, we turned our attention to the synthesis of more challenging C–S and C–P bonds. Unlike the C–Br bond, which can be alternatively prepared using a stoichiometric amount of CuBr, sulfur and phosphine are invalid nucleophiles under Sandmeyer conditions due to the strong coordination of sulfur or phosphine with the Cu cation (completely quenched metal reactivity).

Thiols (RSH) are good nucleophiles in general and can react with arenediazonium salts through an S_N_Ar mechanism with the assistance of a base, especially for acidic thiophenols.^16^ However, as demonstrated above, one major side reaction of the diazonium decomposition is dediazoniation (the formation of Ar–H). This side reaction was more prevalent when using proton-containing nucleophiles (NuH). For example, as shown in Table 3, the reaction of cysteine derivative **6a** with diazonium salt **1a** gave only the dediazoniation product **4a** in 23% yield. The addition of base (2 equiv. of Na_2_CO_3_) did help the formation of the desired thioether **7a** (37% yield), however, a significant amount of the dediazoniation by-product **4a** was obtained (55%). The application of a stoichiometric amount of Cu(OAc)_2_ did not help the reaction at all.

**Table 3:** Gold catalyzed C–S bond formation^*a*^ ^*b*^

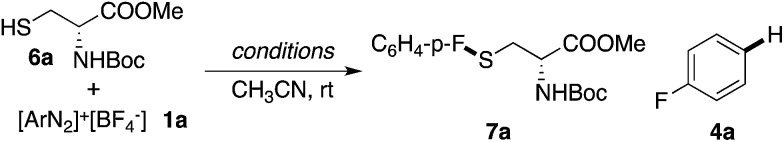					
Catalyst (mol%)	Base (equiv.)	Time	Conv. (%)	**7a** (%)	**4a** (%)
None	None	24 h	30	0	23
None	Na_2_CO_3_ (2)	10 h	100	37	55
Cu(OAc)_2_ (100)	Na_2_CO_3_ (2)	10 h	100	31	65
PPh_3_AuCl (5)	None	10 h	55	49	38
PPh_3_AuCl (5)	Na_2_CO_3_ (2)	3 h	100	87	8
PPh_3_AuCl (3)	Na_2_CO_3_ (2)	3 h	100	86	7
PPh_3_AuCl (1)	Na_2_CO_3_ (2)	7 h	100	53	30

^*a*^Reaction conditions: **1a** (0.2 mmol), **6a** (0.1 mmol), cat. (5 mol%), Na_2_CO_3_ (0.2 mmol) in acetonitrile (ACN), rt.

^*b*^ ^19^F NMR yield with benzotrifluoride as the internal standard.

Interestingly, with PPh_3_AuCl as the catalyst, the desired thioether **7a** was obtained even without a base (49% yield). These results suggest that with the help of a thiol nucleophile, PPh_3_AuCl can be an effective catalyst for diazonium decomposition, forming Au(iii) even at room temperature. With the aid of a base, this challenging C–S coupling was achieved in 86% yield with only a 3 mol% gold catalyst loading. Based on the reaction kinetics, the C–S bond formation was dramatically improved with the gold catalyst.^17^ The reaction scope is shown in Table 4.

**Table 4:** C–S cross-coupling reaction scope^*a*^ ^*b*^

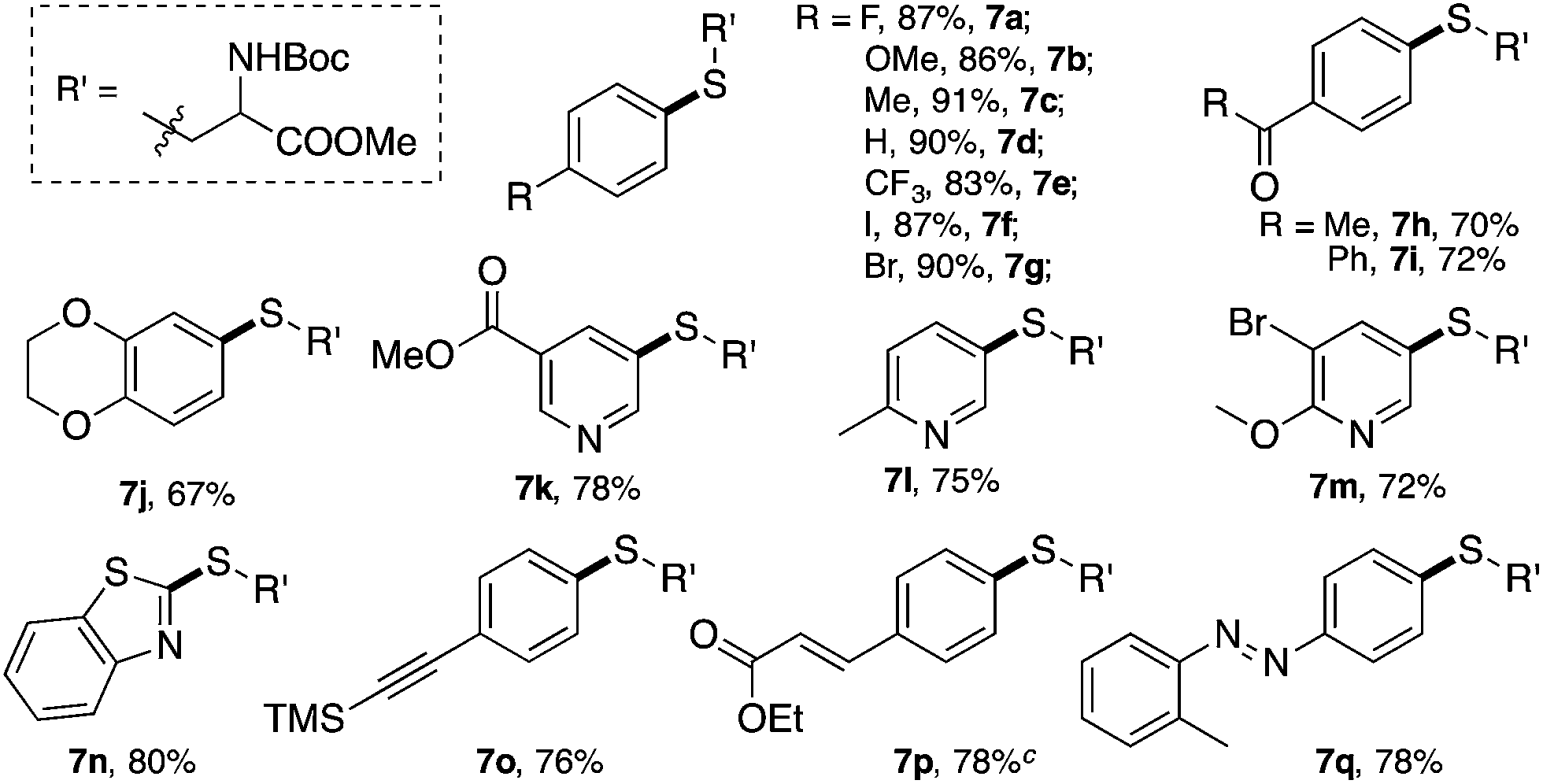

^*a*^Reaction conditions, C–S formation: **1** (0.4 mmol), **6** (0.2 mmol), PPh_3_AuCl (3 mol%), Na_2_CO_3_ (0.4 mmol) in acetonitrile (ACN), rt, 3 h.

^*b*^Isolated yield.

^*c*^Determined using ^1^H NMR with 1,3,5-trimethoxybenzene as the internal standard with *E*/*Z* selectivity of 1 : 1.

Various cysteine derivatives were successfully prepared in good yields. Both electron-rich (**7b**, **7c** and **7j**) and electron-deficient (**7a** and **7e–7i**) diazonium salts were suitable for this transformation with excellent yields. A diazonium salt with an iodide substituent was also tolerated in this reaction (**7f**), which could be a potential synthetic handle for further functionalization. Heterocycles, including various substituted pyridines (**7k–7m**) and benzothiazole (**7n**), gave the desired products efficiently using this catalytic system. An acetylide (**7o**) and α,β-unsaturated ester (**7p**) also reacted with good yields. Notably, sulfur containing molecules, as an important class of compounds for both chemical and biological research, are challenging to construct through traditional cross-coupling strategies because of the potential coordination between sulfur and transition metal catalysts.^18^ This new catalytic system thus provides an efficient strategy to achieve bioactive amino acids.

Our last attempt is to explore the possibility of C–P bond formation using gold redox catalysis. Compared with the C–S bond, C–P bond formation is more challenging as H-phosphonate is much less nucleophilic and it could also be a potential reductant for diazonium salts.^19^ Thus, the C–P bond formation with diazonium salts cannot be achieved through either S_N_Ar or Cu-promoted Sandmeyer reactions. Recently, Toste and coworkers reported the application of a photocatalyst in gold-catalyzed oxidative coupling to achieve this C–P bond formation.^20^ Based on the results discussed above, we wondered whether this nucleophile-promoted gold redox catalysis could be used to achieve this C–P bond formation.

As shown in Table 5, no desired arylphosphonate (**8a**) was obtained using base and/or copper acetate. Impressively, **8a** was formed even with solely PPh_3_AuCl, though in a low yield (25%). The addition of Na_2_CO_3_ did not improve the cross-coupling but promoted Ar–H formation. The combination of PPh_3_AuNTf_2_ and bpy in the presence of Na_2_CO_3_ (previously reported C–C bond coupling conditions) also failed to increase the yield of the desired C–P coupling product. Considering that a nucleophilic ligand is crucial in this gold redox catalysis, we turned our attention to other pyridine derivatives. Through a comprehensive screening, 3-Cl-pyridine was identified as the optimal nucleophile (see detailed screenings in the ESI†), giving the desired C–P bond coupling product **8a** in 83% isolated yield. Using PPh_3_AuNTf_2_ as the catalyst led to a lower yield of **8a** due to the increased yield of the side reactions. Notably, without a gold catalyst, diaza compound **9a** was formed as the major product at room temperature whereas no desired coupling product **8a** was detected.^22^ At 50 °C, the reaction was very messy and **8a** was not detected at all, which suggested that **8a** was not formed from the decomposition of diaza compound **9a**. The reaction substrate scope is shown in Table 6.

**Table 5:** Ligand-assisted gold-catalyzed C–P bond formation^*a*^ ^*b*^

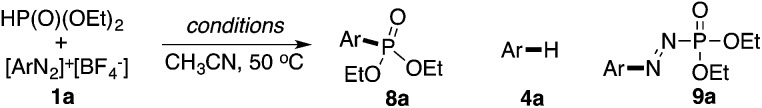						
Catalyst (mol%)	Additives (equiv.)	Time	Conv. (%)	**8a** (%)	**4a** (%)	**9a** (%)
None	None	10 h	50	0	31	0
None	Na_2_CO_3_ (2)	10 h	100	0	70	0
Cu(OAc)_2_ (100)	Na_2_CO_3_ (2)	10 h	100	0	75	0
PPh_3_AuCl (5)	None	10 h	50	25	13	0
PPh_3_AuCl (5)	Na_2_CO_3_ (2)	10 h	100	11	38	0
PPh_3_AuNTf_2_ (5)	bpy (0.2), Na_2_CO_3_ (2)	10 h	100	<5	53	11
PPh_3_AuCl (5)^*c*^	3-Cl-py (2)	3 h	100	83	7	0
PPh_3_AuNTf_2_ (5)^*c*^	3-Cl-py (2)	3 h	100	70	15	0
None^*d*^	3-Cl-py (2)	10 h	69	0	5	44
None	3-Cl-py (2)	10 h	>90	0	25	4

^*a*^Reaction conditions: **1a** (0.2 mmol), HP(O)(OEt)_2_ (0.1 mmol), cat. (5 mol%), base (0.2 mmol) in acetonitrile (ACN), 50 °C.

^*b*^ ^19^F NMR yield with benzotrifluoride as the internal standard.

^*c*^ACN : EtOH = 6 : 1.

^*d*^Room temperature.^21^

**Table 6:** Catalytic C–P cross-coupling reaction scope^*a*^ ^*b*^

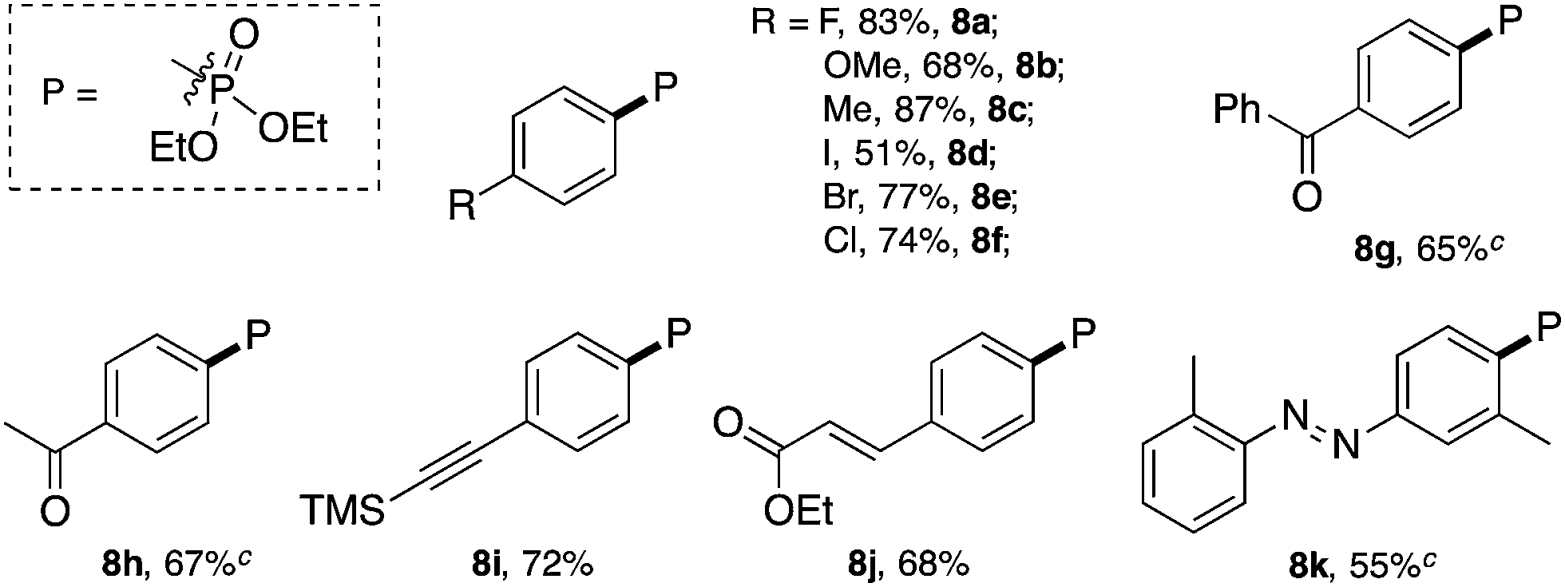

^*a*^Reaction conditions, **1** (0.4 mmol), HP(O)(OEt)_2_ (0.2 mmol), PPh_3_AuCl (5 mol%), 3-Cl-Py (0.4 mmol) in acetonitrile (ACN), 50 °C, 5 h.

^*b*^Isolated yield.

^*c*^Determined using ^1^H NMR with 1,3,5-trimethoxybenzene as the internal standard.

Similar to the C–Br and C–S coupling, a broad substrate scope is observed for the C–P bond formation reactions. Aryl phosphonates with electron rich (**8b** and **8c**) and electron deficient (**8a** and **8d–8h**) substituents could all be generated with good yields. Halogen substituent groups (**8a**, **8e** and **8f**) were all tolerated. An alkyne (**8i**), α,β-unsaturated ester (**8j**) and azobenzene (**8k**) also gave good results, suggesting the great synthetic potential of this methodology.

## Conclusions

In summary, we reported C–Br, C–S, and C–P bond formation through gold redox catalysis. We demonstrated that nucleophiles play a crucial role in the Au(i) promoted diazonium decomposition. With this strategy, various C–X couplings could be achieved with excellent yields and a broad substrate scope simply using LAuCl (no need for an external oxidant). These results not only provide a new practical strategy to achieve challenging C–X bond couplings, but also, more importantly, reveal some new mechanistic insight regarding gold redox catalysis, which will likely further enrich the pedigree of gold catalysis.

## Supplementary Material

Supplementary information
