# Supplementary material for: Nucleophile promoted gold redox catalysis with diazonium salts: C–Br, C–S and C–P bond formation through catalytic Sandmeyer coupling
Source: Chem Sci. 2016 Jun 10;7(9):6190–6. doi: 10.1039/c6sc01742h (PMC6024175; doi:10.1039/c6sc01742h)
Supplement: Supplementary file 1 [file SC-007-C6SC01742H-s001.pdf]

Electronic Supplementary Material (ESI) for Chemical Science.
This journal is © The Royal Society of Chemistry 2016

Supporting Information

Aryldiazonium Promoted Gold-Redox Catalysis: C-Br, C-P and C-S Bond Formation through Catalytic Sandmeyer Coupling

Haihui Peng,[a] Rong Cai,[b] Chang Xu,[c] Hao Chen[c] and Xiaodong Shi*,[a]

[a] *Department of Chemistry, University of South Florida, Tampa, FL 33620, USA*

[b] *C. Eugene Bennett Department of Chemistry, West Virginia University, Morgantown, WV 26506, USA*

[c] *Center for Intelligent Chemical Instrumentation, Department of Chemistry and Biochemistry, Edison Biotechnology Institute,, Ohio University, Athens, OH 45701, USA*

I. General Methods and Materials **S2**

II. General Procedures **S2-S4**

III. Stoichiometric experiment with Au complex **S5-S7**

IV. Kinetics Experiments with Au/non-metal C-S bond formation **S8-S10**

V. Exploring the Au(III) intermediate in Au(I) oxidation by ESI-MS **S11-S15**

VI. Compounds Characterization **S16-S28**

VII. NMR Spectra of New Compounds **S29-S105**

I. General Methods and Materials

All of the reactions dealing with air and/or moisture-sensitive reactions were carried out under an atmosphere of nitrogen using oven/flame-dried glassware. Unless otherwise noted, all commercial reagents and solvents were obtained from the commercial provider and used without further purification. PPh_3AuCl, PPh_3AuNTf_2 were synthesized according to literature report. ^1H NMR, ^{13}C NMR, ^{31}P NMR, and ^{19}F NMR spectra were recorded on Agilent 400 MHz or Varian 600 MHz spectrometers. Chemical shifts were reported relative to internal tetramethylsilane (δ 0.00 ppm) or $CDCl_3$ (δ 7.26 ppm) for ^1H and $CDCl_3$ (δ 77.0 ppm) for ^{13}C. Flash column chromatography was performed on 230-430 mesh silica gel.

II. General Procedures

A. General procedure of conditions for Au(I) catalyzed C-X bond foramtion

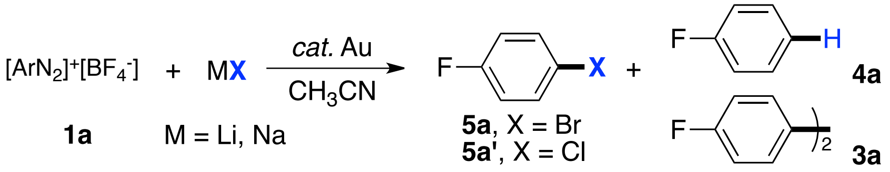

In a dried glass tube, **1a** (0.1 mmol), [Au] (0.005 mmol, 5 mol %) and MX (0.3 mmol, 3 equiv) were dissolved in CH_3CN (0.3 mL). The reaction mixture was stirred at 50 °C for 5-12 h. After the reaction completed, the reaction was filtrate through a pad of silica gel. After evacuation of the solvents, the NMR yields were obtained by ^{19}F NMR analysis of the crude mixture with the internal standard of benzotrifluoride. The results were summarized in Table S1.

Table S1. Screening of conditions

entry	cat.Au(%)	MX	Solvent	Time/h	convn (%)[a]	yield (%)[a,b]		
						**5a**	**3a**	**4a**
1	None	LiCl	CH_3CN	50 °C, 12 h	50	<10	Trace	33
2	5% Ph_3PAuCl	LiCl	CH_3CN	50 °C, 12 h	70	**5a'**, <5	Trace	38
3	5% $Ph_3PAuNTf_2$	LiCl	CH_3CN	50 °C, 12 h	77	**5a'**, <5	Trace	43
4	5% $Ph_3PAuNCl$	NaBr	CH_3CN	50 °C, 12 h	100	**5a**, 51	11	23
5	5% $Ph_3PAuNTf_2$	NaBr	CH_3CN	50 °C, 12 h	100	**5a**, 58	9	18
6	5% $Ph_3PAuNTf_2$	LiBr	CH_3CN	50 °C, 12 h	100	**5a**, 68	6	15
7	5% $Ph_3PAuNTf_2$	LiBr	CH_3CN	20% bpy, 50 °C, 5 h	100	**5a**, 63	8	15
8	5% Ph_3PAuCl	LiBr	CH_3CN	50 °C, 5 h	100	**5a**, 83	7	Trace
9	3% Ph_3PAuCl	LiBr	CH_3CN	50 °C, 5 h	100	**5a**, 81	7	<5
10	1% Ph_3PAuCl	LiBr	CH_3CN	50 °C, 5 h	100	**5a**, 63	13	9
11	5% Ph_3PAuCl	LiBr	Acetone	50 °C, 5 h	100	**5a**, 11	37	Trace

[a] Reaction conditions: **1a** (0.1 mmol), Au (5 mol%), MX (0.3 mmol), additives and solvent (0.33M); [b] Determined by ^{19}F NMR using benzotrifluoride as internal standard; [c] The major byproduct is biaryl; [d] Yield of biaryl:37%.

B. General procedure of conditions for Au(I) catalyzed C-P bond formation

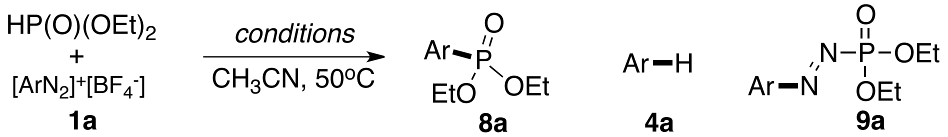

In a dried glass tube, **1a** (0.2 mmol, 2 equiv), [Au] (0.005 mmol, 5 mol %), $HP(O)(OEt)_2$ (0.1 mmol, 1 equiv) and additives (2 equiv) were dissolved in CH_3CN (0.3 mL). The reaction mixture was stirred at 50 °C for 5-12 h. After the reaction completed, the reaction was filtrate through a pad of silica gel. After evacuation of the solvents, the NMR yields were obtained by ^{19}F NMR analysis of the crude mixture with the internal standard of benzotrifluoride. The results were summarized in Table S2.

Table S2. Screening of conditions

entry	cat.Au(%)	Additive(2 equiv)	Solvent	Time/h	convn (%)[a]	yield (%)[a,b]		
						**8a**	**4a**	**9a**
1	None	None	CH_3CN	10 h	50	0	31	0
2	None	Na_2CO_3	CH_3CN	10 h	100	0	70	0
3	1 eq $Cu(OAc)_2$	Na_2CO_3	CH_3CN	10 h	100	0	75	0
4	5% PPh_3AuCl	None	CH_3CN	10 h	50	25	13	0
5	5% PPh_3AuCl	Na_2CO_3	CH_3CN	10 h	100	11	38	0
6	5% PPh_3AuNTf_2	20% bpy, Na_2CO_3	CH_3CN	10 h	100	<5	53	11
7	5% PPh_3AuNTf_2	3-Cl-py	CH_3CN	5h	100	67	16	0
8	5% PPh_3AuCl[c]	3-Cl-py	CH_3CN	5 h	100	73	15	0
9	None [d]	3-Cl-py	CH_3CN	10 h	69	0	5	44
10	None	3-Cl-py	CH_3CN	10h	>90	0	25	4
11	5% PPh_3AuCl	Py	CH_3CN	2 h	100	9	36	0
12	5% PPh_3AuCl	2,6-Lutidine	CH_3CN	3 h	100	26	26	0
13	5% PPh_3AuCl	DMAP	CH_3CN	0.5 h	100	0	71	0
14	5% Ph_3PAuCl	3-Cl-Py	$CH_3CN/EtOH = 6:1$	3 h	100	83	7	0
15	3% PPh_3AuCl	3-Cl-py	$CH_3CN/EtOH = 6:1$	5 h	100	70	13	0
16	1% PPh_3AuCl	3-Cl-py	$CH_3CN/EtOH = 6:1$	7 h	100	51	18	0

[a] Reaction conditions: **1a** (0.2 mmol), Au (5 mol%), $HP(O)(OEt)_2$ (0.1 mmol), additives and solvent (0.33M); [b] Determined by ^{19}F NMR using benzotrifluoride as internal standard; [c] The major byproduct is biaryl, yield of biaryl:37%; [d] room temperature.

C. General procedure for Au(I) catalyzed C-Br bond formation

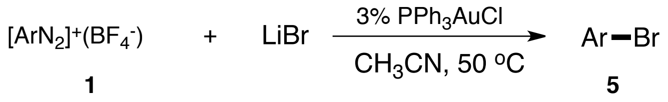

In a dried glass tube, **1** (0.2 mmol), PPh_3AuCl (0.010 mmol, 5 mol %) and LiBr (0.6 mmol, 3 equiv) were dissolved in CH_3CN (0.5 mL). The reaction mixture was stirred at 50 °C for 5 h. After the reaction completed, the reaction mixture was directly put on the column to obtain the product.

D. General procedure for Au(I) catalyzed C-S bond formation

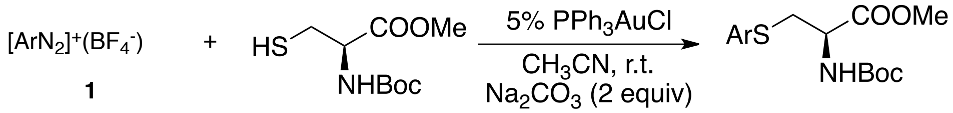

In a dried glass tube, **1** (0.4 mmol, 2 equiv), PPh_3AuCl (0.010 mmol, 5 mol %), Na_2CO_3 (2 equiv) and **4** (0.2 mmol, 1 equiv) were dissolved in CH_3CN (0.5 mL). The reaction mixture was stirred at room temperature for 3 h. After the reaction completed, the reaction mixture was directly put on the column to obtain the product.

E. General procedure for Au(I) catalyzed C-P bond formation

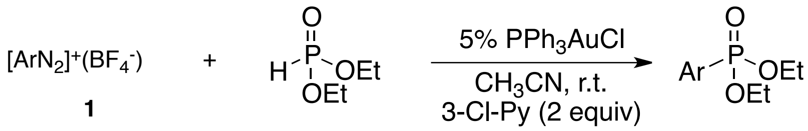

In a dried glass tube, **1** (0.4 mmol, 2 equiv), PPh_3AuCl (0.010 mmol, 5 mol %), diethyl phophite (0.2 mmol, 1 equiv) and 3-Cl-Py (0.4 mmol, 1 equiv) were dissolved in CH_3CN (0.5 mL). The reaction mixture was stirred at 50 °C for 8 h. After the reaction completed, the reaction mixture was directly put on the column to obtain the product.

III. Stoichiometric experiment with PPh_3Au complex

A. Stoichiometric experiments of diazonium with PPh_3AuCl

In a dried glass tube, **1a** (0.036 mmol, 1.1 equiv), Nu^- (1 equiv) and PPh_3AuCl (0.033 mmol, 1 equiv) were dissolved in CD_3CN (0.3 mL). The reaction mixture was stirred at 50 °C for 10h. The results were summarized in **Figure S1**. By using bpy as nucleophile, the reaction kinetics was also monitored with ^{31}P NMR, the results were summarized in Figure **S2**.

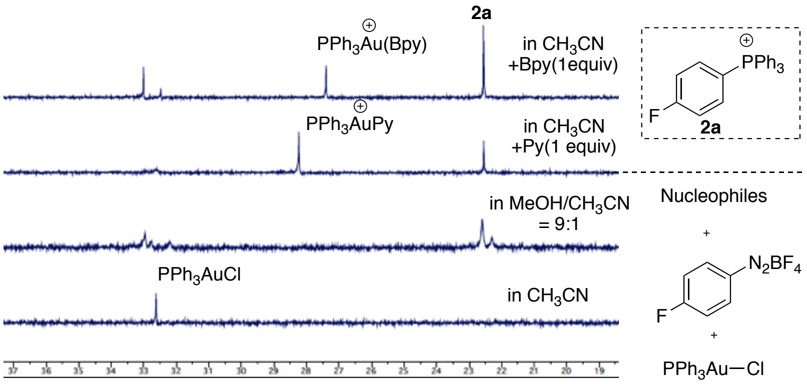

Figure S1. ^{31}P NMR of PPh_3AuCl with diazonium **1a** in the presence of nucleophiles

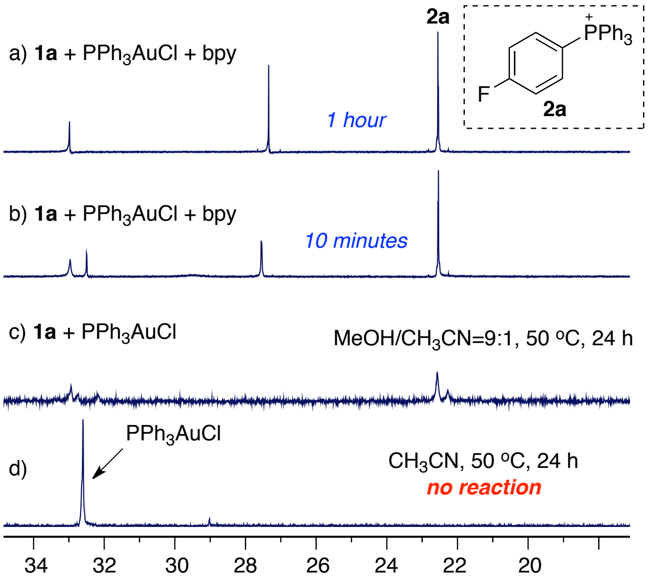

Figure S2. ^{31}P NMR of PPh_3AuCl with diazonium **1a** in the presence of bpy

B. Stoichiometric experiments of diazonium with PPh_3AuNTf_2

In a dried glass tube, **1a** (0.05 mmol, 1.5 equiv) and PPh_3AuNTf_2 (0.033 mmol, 1 equiv) were dissolved in CD_3CN (0.3 mL). The reaction mixture was stirred at 50 ^{o}C for 10 h. The reaction was monitored by ^{31}P NMR and ^{19}F NMR. The results were summarized in **Figure S3** and **Figure S4**.

C. Stoichiometric experiments of diazonium with PPh_3AuCl

In a dried glass tube, **1a** (0.05 mmol, 1.5 equiv) and PPh_3AuCl (0.033 mmol, 1 equiv) were dissolved in CD_3CN (0.3 mL). The reaction mixture was stirred at 50 ^{o}C for 10 h. The reaction was monitored by ^{31}P NMR and ^{19}F NMR. The results were summarized in **Figure S3** and **Figure S4**.

D. Stoichiometric experiments of diazonium with PPh_3AuNTf_2 and 1 equiv LiCl

In a dried glass tube, **1a** (0.05 mmol, 1.5 equiv), PPh_3AuNTf_2 (0.033 mmol, 1 equiv) and LiCl (0.033 mmol, 1 equiv) were dissolved in CD_3CN (0.3 mL). The reaction mixture was stirred at 50 ^{o}C for 10 h. The reaction was monitored by ^{31}P NMR and ^{19}F NMR. The results were summarized in **Figure S3** and **Figure S4**.

E. Stoichiometric experiments of diazonium with PPh_3AuNTf_2 and 10 equiv LiCl

In a dried glass tube, **1a** (0.05 mmol, 1.5 equiv), PPh_3AuNTf_2 (0.033 mmol, 1 equiv) and LiCl (0.33 mmol, 10 equiv) were dissolved in CD_3CN (0.3 mL). The reaction mixture was stirred at 50 ^{o}C for 10 h. The reaction was monitored by ^{31}P NMR and ^{19}F NMR. The results were summarized in **Figure S3** and **Figure S4**.

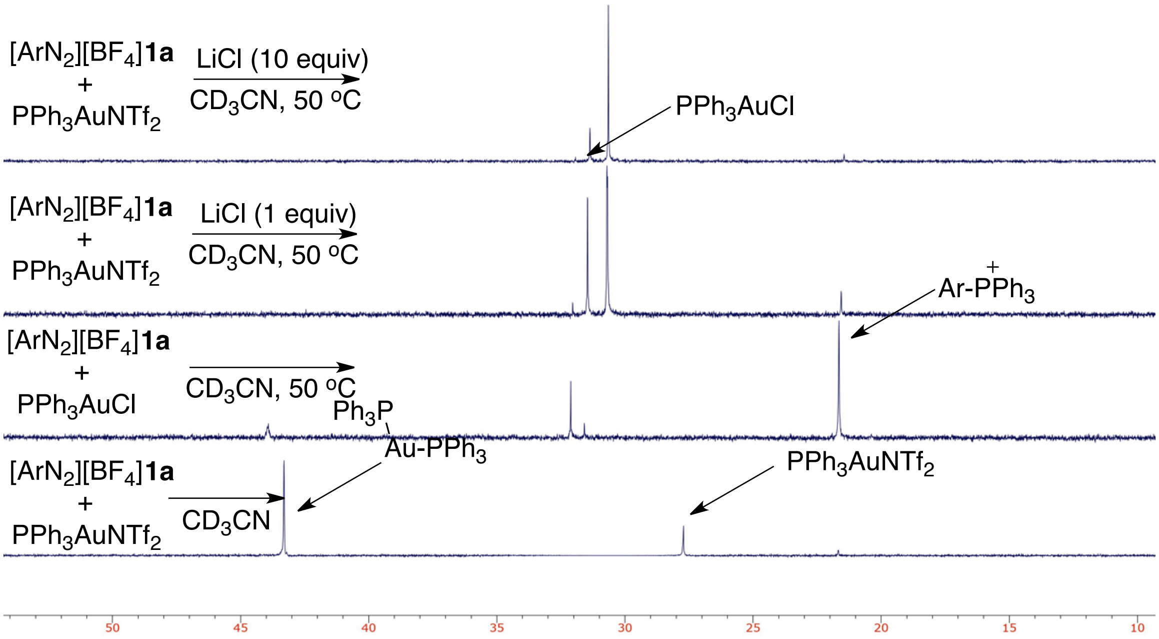

Figure S3. ^{31}P NMR analysis of stoichiometric experiments

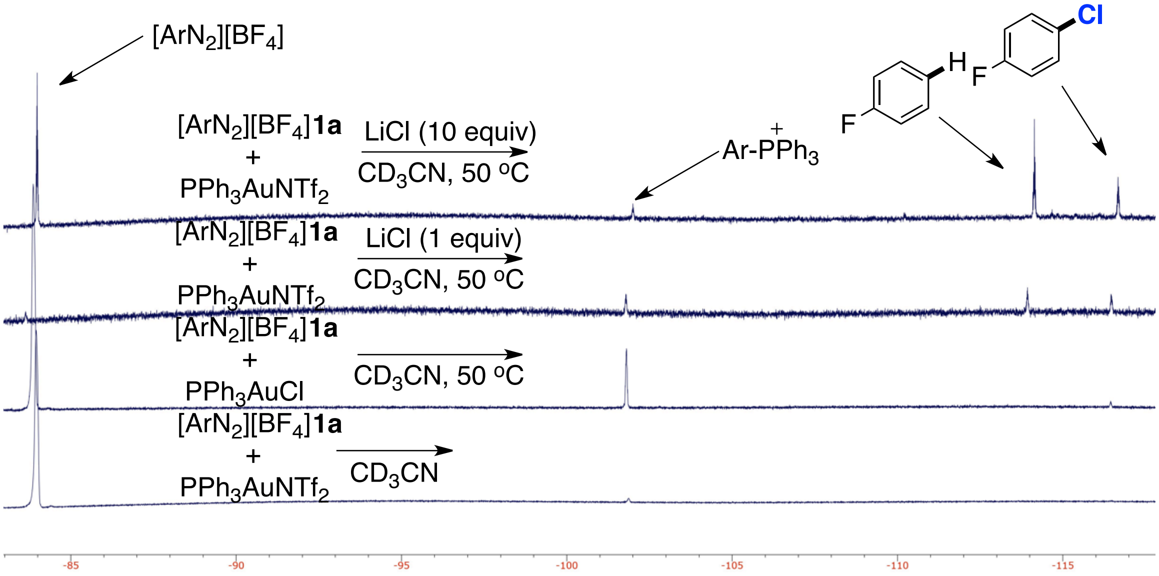

Figure S4. ^{19}F NMR analysis of stoichiometric experiments

IV. Kinetics Experiments for C-S bond formation

A. Kinetics Experiments for Au-catalyzed C-S bond formation

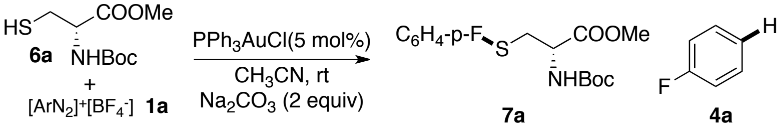

In a dried glass tube, **1a** (0.4 mmol, 2 equiv), PPh_3AuCl (0.010 mmol, 5 mol %), Na_2CO_3 (0.4 mmol, 2 equiv) and **4** (0.2 mmol, 1 equiv) were dissolved in CD_3CN (0.5 mL). The reaction mixture was stirred at room temperature for 3 h. The reaction was monitored by ^{19}F NMR analysis with the internal standard of benzotrifluoride with different reaction time. The results were summarized in **Figure S5**.

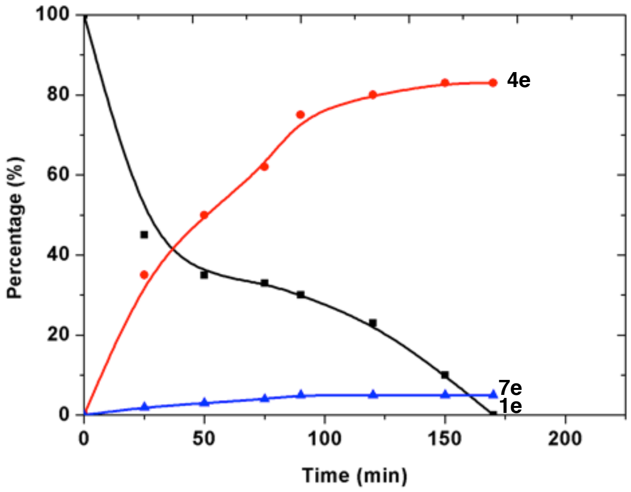

Figure S5. PPh_3AuCl catalyzed C-S bond formation

B. Kinetics Experiments for C-S bond formation without gold catalyst

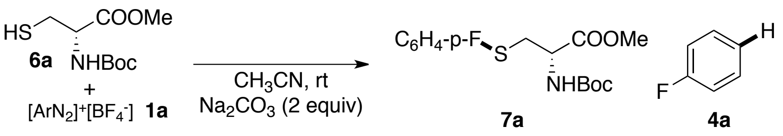

In a dried glass tube, **1a** (0.4 mmol, 2 equiv), Na_2CO_3 (0.4 mmol, 2 equiv) and **4** (0.2 mmol, 1 equiv) were dissolved in CD_3CN (0.5 mL). The reaction mixture was stirred at room temperature for 3 h. The reaction was monitored by ^{19}F NMR analysis with the internal standard of benzotrifluoride with different reaction time. The results were summarized in **Figure S6**.

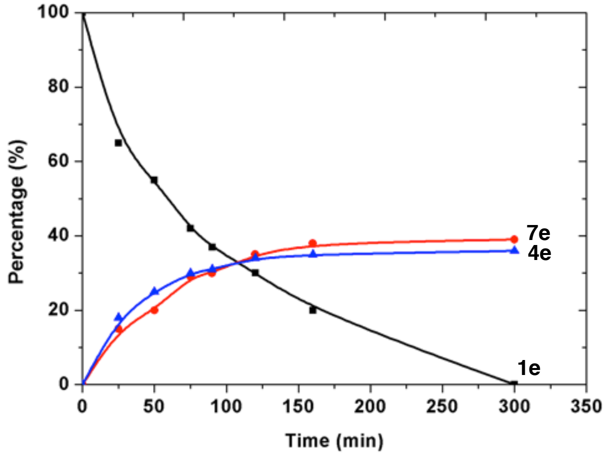

Figure S6. Na_2CO_3 promoted C-S bond formation

C. Kinetics Experiments for C-S bond formation without base and catalyst

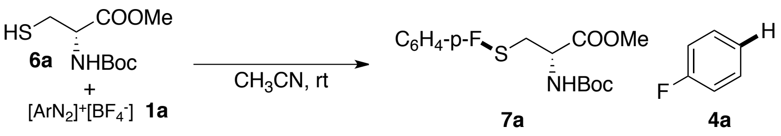

In a dried glass tube, **1a** (0.4 mmol, 2 equiv) and **4** (0.2 mmol, 1 equiv) were dissolved in CD_3CN (0.5 mL). The reaction mixture was stirred at room temperature for 3 h. The reaction was monitored by ^{19}F NMR analysis with the internal standard of benzotrifluoride with different reaction time. The results were summarized in **Figure S7**.

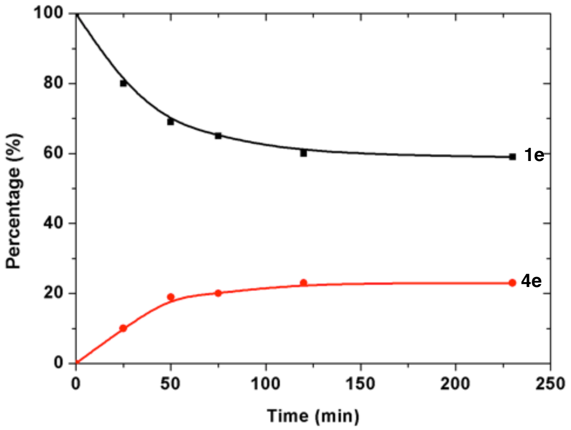

Figure S7. No metal or base promoted reaction

V. Exploring the Au(III) intermediate in Au(I) oxidation by electrospray ionization mass spectrometry (ESI-MS).

ESI-MS spectra were collected using a Waters Xevo QTof mass spectrometer (Milford, MA, USA) in the positive ion mode. The samples were infused and sprayed at a flow rate of 10 μL/min with an applied high voltage of 5 kV.

20 mM PPh_3AuCl was reacted with NaBr at a 1:3 ratio in CH_3CN and was stirred at room temperature for 12 h. The solution was further stirred at 50 ^{o}C for 1 h on the next day. Then 20 mM of aryldiazonium **2a** was added to the reaction mixture and stirred for 1 h. The reaction solution was diluted to 500 μM using CH_3CN and subsequently analyzed using ESI-MS. The acquired MS data is shown in Figure **S8**. Besides the aruldiazonium ion [Ar-N_2^+] (*m/z* 123) seen in the spectrum, a Au(III) complex ion [$Ph_3PAuAr(Br)_2$ + Ar-N_2^+] is also detected at *m/z* 835 (Figure **S8**).

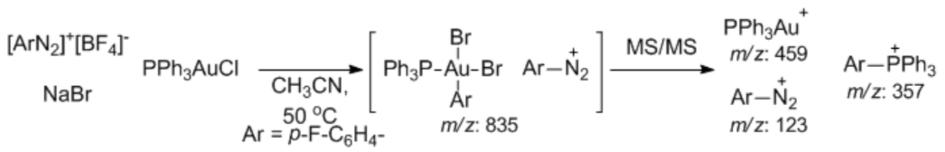

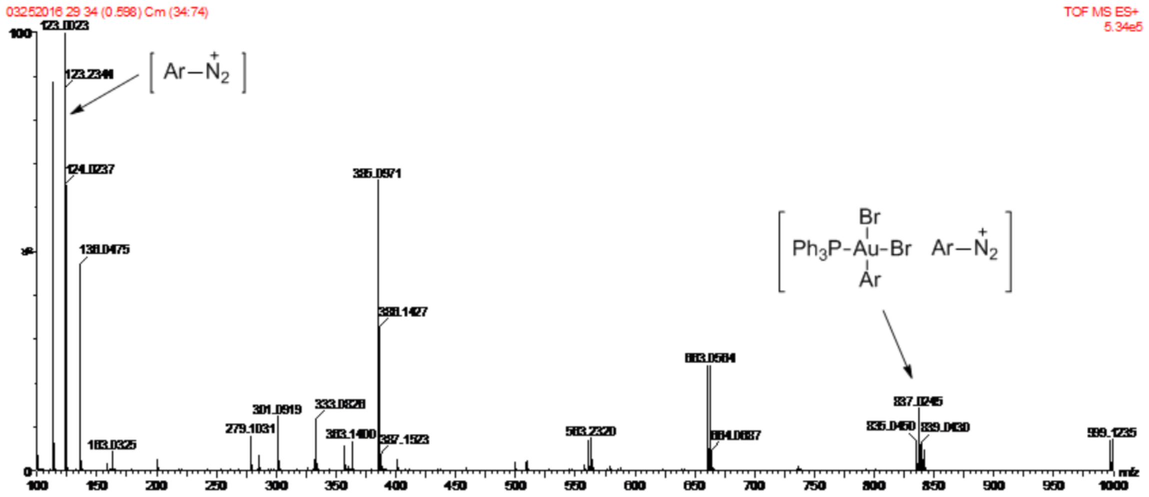

Figure S8. ESI-MS spectrum of the reaction mixture with NaBr.

Tandem MS analysis (MS/MS) was used to characterize the structures of assigned ions. Upon collision induced dissociation (CID), *m/z* 835 gave rise to fragment ions [Ph_3PAu^+] (*m/z* 459), [Ar-PPh_3] (*m/z* 357), [Ar-N_2^+] (*m/z* 123), consistent with the assigned Au(III) ion structure for *m/z* 835. (Figure **S9**).

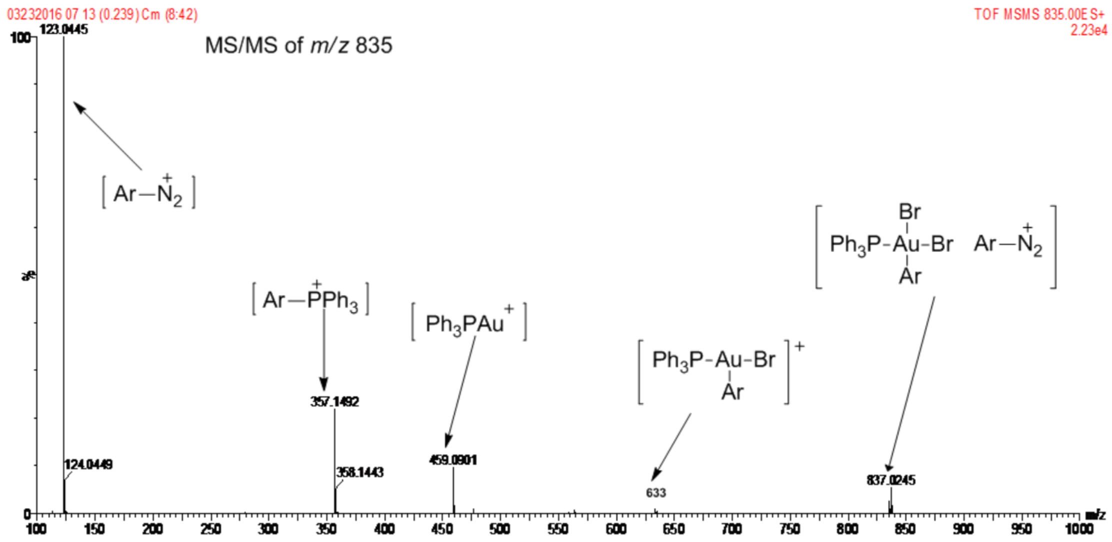

Figure S9. CID MS-MS spectrum of *m/z* 835

The reaction was also examined using LiBr to replace NaBr. In the experiment, 20 mM PPh_3AuCl was reacted with LiBr in ACN at a 1:3 ratio and was stirred at room temperature for 12 h. The solution was stirred at 50 ^oC for 1 h on the next day. Then 20 mM of aryldiazonium **2a** was added to the reaction mixture and stirred for 1 hr. The reaction solution was diluted to 500 µM using CH_3CN and subsequently analyzed using ESI-MS. The acquired MS data is shown in Figure **S10**. Beside [Ph_3PAuBr^+] (*m/z* 545), [Ar-PPh_3^+] (*m/z* 357), [Ar-N_2^+] (*m/z* 123) seen in the spectrum, two solid (III) complex ions, [$Ph_3PAuAr(Br)_2$+ Li^+] (*m/z* 719), [$Ph_3PAuAr(Br)_2$+ ArN_2^+] (*m/z* 835) are also detected.

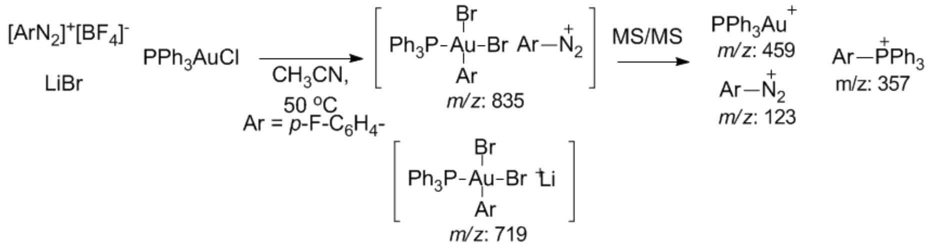

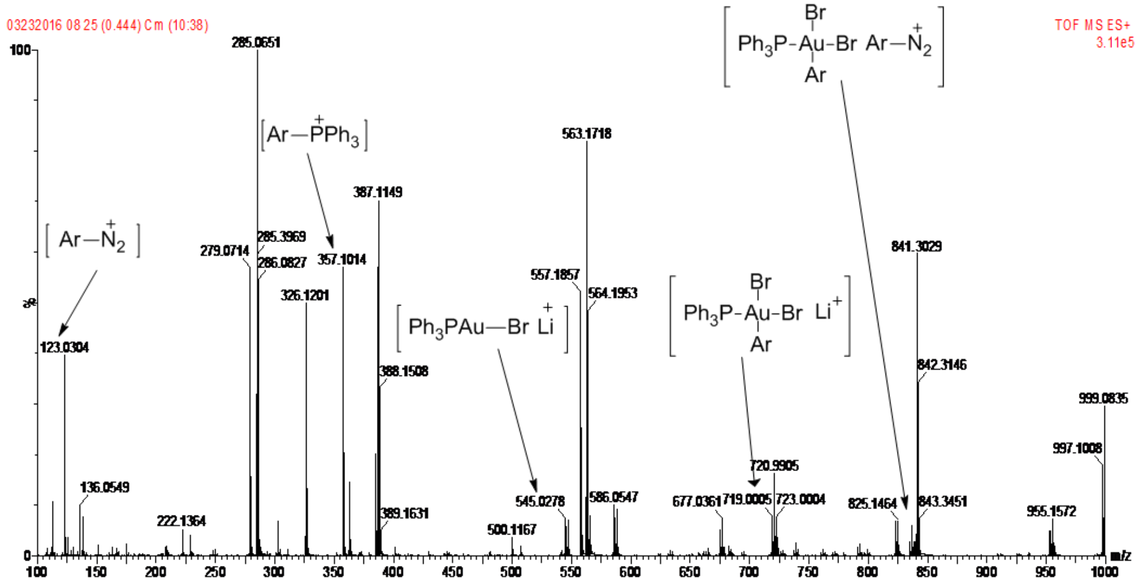

Figure S10. ESI-MS spectrum of the reaction mixture with LiBr

4 mM PPh_3AuCl was reacted with equal concentration of aryldiazonium and 2,2'-bipyridyl (bpy) at a 1:1:1 ratio in CH_3CN and was stirred at room temperature for 30 min. The reaction solution was diluted to 50 µM using CH_3CN and subsequently analyzed using ESI-MS and the sample injected flow rate was 5 µL/min. In addition to [$Ar-N_2^+$] (*m/z* 123), [$bpy+H^+$] (*m/z* 157), [$bpy+Ar-N_2^+$] (*m/z* 279), [$bpy+Ph_3PAu^+$] (*m/z* 615), and [$Cl^-+2(Ph_3PAu^+)$] (*m/z* 953) observed in the acquired MS spectrum (**Figure S-X)**, an Au(III) complex ion $[Ph_3PAuAr(Cl)(bpy)]^+$ was also detected at *m/z* 745.

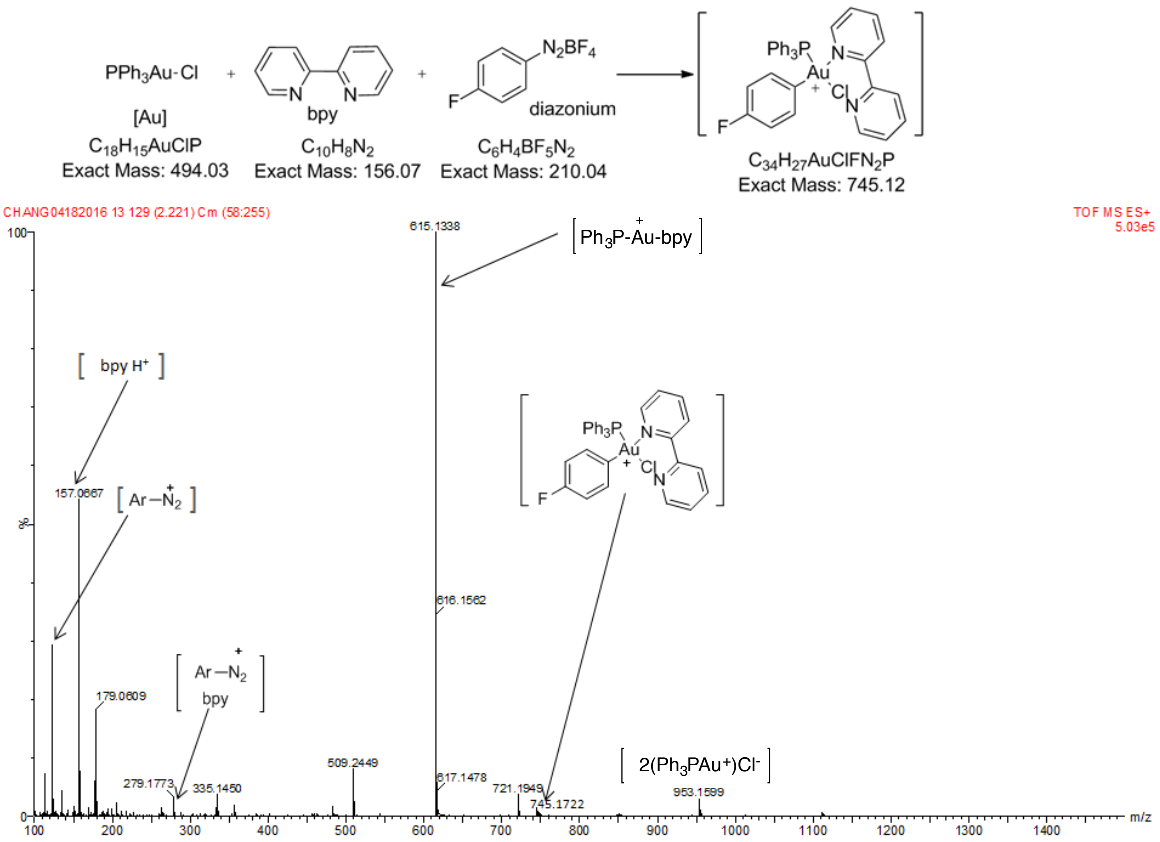

Figure S-X. ESI-MS spectrum of the reaction mixture with bpy

Tandem MS analysis (MS/MS) was used to characterize the structures of assigned ions. Upon collision induced dissociation (CID), *m/z* 745 gave rise to fragment ions [Ar-PPh_3^+] (*m/z* 357) and [Ar-AuCl(bpy)]$^+$ (*m/z* 483), consistent with the assigned Au(III) ion structure for *m/z* 745. (Figure **S-Y**).

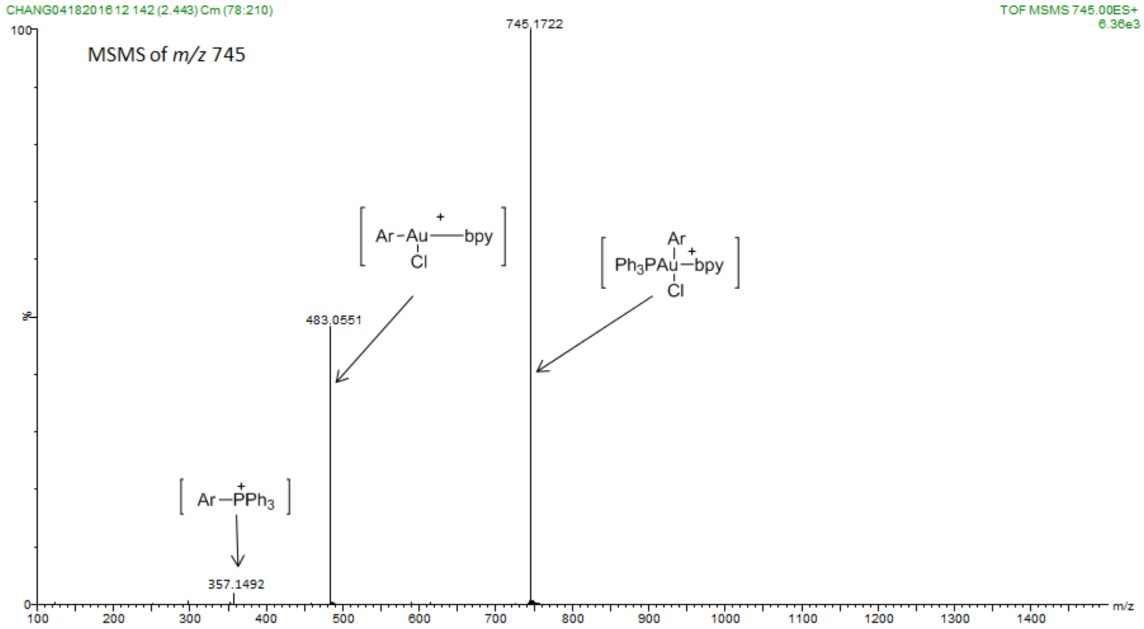

Figure S12. CID MS-MS spectrum of *m/z* 745

V. Compounds Characterization

Compounds **5a**, **5b**, **5c**, **5d**, **5e**, **5f**, and **5g** are commercially available and volatile compounds. **5h**,[1] **5i**,[2] **5j**,[3] **5k**,[4] **5m**,[5] **5n**,[6] **5o**,[7] **5p**,[8] **5r**,[9] 5s,[10] **8a**, **8c**,[11] **8d**, **8e**,[12] **8g**,[13] and **8h**[14]were reported in literature.

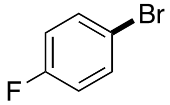

5a

CAS: 460-00-4, GC-MS: 174.0, 95.1, 87.1, 75.1, 68.1, 50.1.

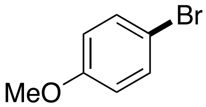

5b

CAS: 104-92-7, GC-MS:187.8, 170.8, 142.9, 118.9, 92.0, 77.0, 63.0.

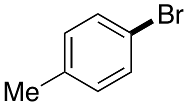

5c

CAS: 106-38-7, GC-MS: 169.9, 91.0, 65.0.

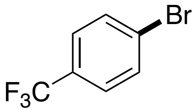

5d

CAS: 402-43-7, GC-MS: 223.8, 204.8, 173.8, 144.9, 125.0, 95.0, 75.0.

---

[1]Rühling, A.; Galla, H.–J.; Glorius, F. *Chem. Eur. J.* **2015**, *21*, 12291.
[2]Gonzalez-de-Castro, A.; Xiao, J. *J. Am. Chem. Soc.*, **2015**, *137,* 8206.
[3]Skillinghaug, B.; Sköld, C.; Rydfjord, J.; Svensson, F.; Behrends, M.; Sävmarker, J.; Sjöberg, J. R.; Larhed, M. *J. Org. Chem.*, **2014**, *79,*12018.
[4]Thomé, I.; Besson, C.; Kleine, T.; Bolm, C. *Angew. Chem. Int. Ed.* **2013**, *52,*7509.
[5]Thompson, W. J.; Gaudino, J. *J. Org. Chem.*, **1984**, *49,*5237.
[6]Hibi, S.; Ueno, K.; Nagato, S.; Kawano, K.; Ito, K.; Norimine, Y.; Takenaka, O.; Hanada, T.; Yonaga, M. *J. Med. Chem.*, **2012**, *55,* 10584.
[7]Arora, A.; Teegardin, K.; Weaver, J. D. *Org. Lett.*, **2015**, *17,* 3722.
[8]Uemura, Y.; Mori, S.; Hara, K.; Koumura, N. *Chem Lett.* **2011**, *40*, 872
[9]Uptmoor, A. C.; Freudenberg, J.; Schwäbel, S. T.; Paulus, F.; Rominger, F.; Hinkel, F.; Bunz, U. H. F. *Angew. Chem. Int. Ed.* **2015**, *54*,14673.
[10] Tani, M.; Sakaguchi, S.; Ishii, Y. J. Org. Chem., 2004, 69, 1221.
[11] He, Y.; Wu, H.; Toste, F. D. *Chem. Sci.*, **2015**, *6*, 1194.
[12] Ballester, J.; Gatignol, J.; Schmidt, G.; Alayrac, C.; Gaumont, A.-C.; Taillefer, M. *ChemCatChem*, **2014**, *6*, 1549.
[13] Amatore, C.; Gareil, M.; Octuran, M. A.; Pinson, J.; Saveant, J. M.; Thiebault, A. *J. Org. Chem*, **1986**, *51*, 3757.
[14] Zhuang, R.; Xu, J.; Cai, Z.; Tang, G.; Fang, M.; Zhao, Y. *Org. Lett.*, **2011**, *13,* 2110.

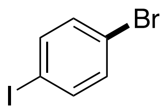

5e

CAS: 589-87-7, GC-MS: 281.6, 154.8, 140.9, 126.8, 75.0.

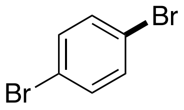

5f

CAS: 106-37-6, GC-MS: 235.7, 154.8, 117.9, 75.0.

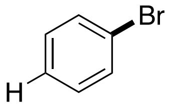

5g

CAS: 108-86-1, GC-MS: 155.8, 77.0, 51.0.

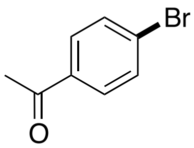

5h

^{1}H NMR (400 MHz, $CDCl_3$): δ 7.82 (dt, *J* = 1.6, 8.4 Hz, 2H), 7.61 (dt, *J* = 1.6, 8.8 Hz, 2H), 2.61 (s, 3H).
^{13}C NMR (100 MHz, $CDCl_3$): δ 197.0, 135.8, 131.9, 129.8, 128.3, 26.5.

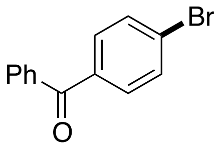

5i

^{1}H NMR (400 MHz, $CDCl_3$): δ 7.77 (d, *J* = 7.2 Hz, 2H), 7.67 (dt, *J* = 2.0, 8.8 Hz, 2H), 7.61 (m, 3H), 7.49 (t, J = 7.6 Hz, 2H).
^{13}C NMR (100 MHz, $CDCl_3$): δ 195.6, 137.1, 136.3, 132.6, 131.6, 131.5, 129.9, 128.4, 127.5.

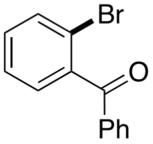

5j

^{1}H NMR (400 MHz, $CDCl_3$): δ 7.81 (dd, *J* = 1.2, 8.4 Hz, 2H), 7.63 (m, 2H), 7.45 (m, 3H), 7.35 (m, 2H).
^{13}C NMR (100 MHz, $CDCl_3$): δ 195.9, 140.7, 136.1, 133.8, 133.2, 131.2, 130.3, 129.0, 128.7, 127.2, 119.5.

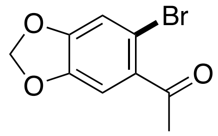

5k

^{1}H NMR (400 MHz, $CDCl_3$): δ 7.04 (s, 1H), 7.03 (s, *1*H), 6.04 (s, 2H), 2.61 (s, 3H).
^{13}C NMR (100 MHz, $CDCl_3$): δ 199.4, 150.3, 147.3, 134.2, 113.8, 109.8, 102.4, 33.8, 30.2.

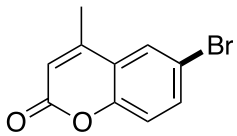

5l

^{1}H NMR (400 MHz, $CDCl_3$): δ 7.45 (m, 3H), 6.29 (q, *J* = 1.6 Hz, 1H), 2.38 (q, *J* = 1.6 Hz, 3H).
^{13}C NMR (100 MHz, $CDCl_3$): δ 159.9, 153.7, 151.8, 127.5, 125.6, 125.5, 120.1, 118.9, 115.2, 18.5.
HRMS: m/z (ESI) Calculated for $[M+H]^+$ 238.9708, Found 238.9688.

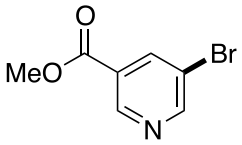

5m

^{1}H NMR (400 MHz, $CDCl_3$): δ 9.13 (d, *J* = 2.0 Hz, 1H), 8.84 (d, *J* = 2.0 Hz, 1H), 8.43 (d, *J* = 2.0 Hz, 1H), 3.97 (s, 3H).
^{13}C NMR (100 MHz, $CDCl_3$): δ 164.5, 154.5, 148.8, 139.5, 127.3, 120.6, 52.7.

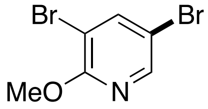

5n

^{1}H NMR (400 MHz, DMSO-d_6): δ 8.30 (d, *J* = 2.4 Hz, 1H), 8.27 (d, *J* = 2.4 Hz, 1H), 3.88 (s, 3H).

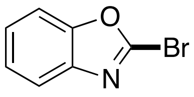

5o

^{1}H NMR (500 MHz, $CDCl_3$): δ 7.99 (d, *J* = 7.0 Hz, 1H), 7.81 (dd, *J* = 1.5, 8.5 Hz, 1H), 7.47 (dt, *J* = 1.0, 8.5 Hz, 1H), 7.42 (dt, *J* = 1.5, 8.0 Hz, 1H).
^{13}C NMR (125 MHz, $CDCl_3$): δ 152.3, 138.9, 137.3, 126.6, 125.7, 122.8, 120.9.

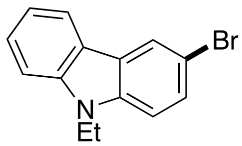

5p

^{1}H NMR (500 MHz, $CDCl_3$): δ 8.20 (d, *J* = 2.0 Hz, 1H), 8.04 (d, *J* = 3.0 Hz, 1H), 7.53 (dd, *J* = 2.0, 9.0 Hz, 1H), 7.47 (ddd, *J* = 1.0, 7.0 Hz, 1H), 7.40 (d, *J* = 8.0 Hz, 1H), 7.26 (m, 2H), 4.34 (q, J = 7.5 Hz, 2H), 1.4 (t, J = 7.5 Hz, 3H).
^{13}C NMR (125 MHz, $CDCl_3$): δ 140.2, 138.5, 128.2, 126.3, 124.7, 123.1, 121.9, 120.6, 119.2, 111.5, 109.9, 108.7, 37.7, 13.7.

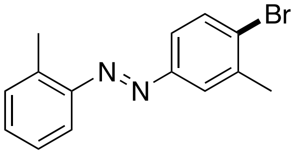

5q

^{1}H NMR (400 MHz, $CDCl_3$): δ 7.55 (s, 1H), 7.59 (m, 3H), 7.31 (m, 2H), 7.23 (m, 1H), 2.69 (s, 3H), 2.46 (s, 3H).
^{13}C NMR (100 MHz, $CDCl_3$): δ 152.0, 150.6, 138.8, 138.3, 133.0, 131.4, 131.2, 127.6, 126.5, 125.3, 121.4, 115.4, 23.1, 17.6.
HRMS: m/z (ESI) Calculated for $[M+NH]^+$ 289.0340, Found 289.0335.

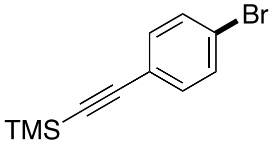

5r

^{1}H NMR (500 MHz, $CDCl_3$): δ 7.52 (d, *J* = 8.0 Hz, 2H), 7.41 (d, *J* = 8.0 Hz, 2H), 0.33 (s, 9H).
^{13}C NMR (125 MHz, $CDCl_3$): δ 133.4, 131.5, 122.7, 122.1, 103.8, 95.6, 29.7, -0.1.

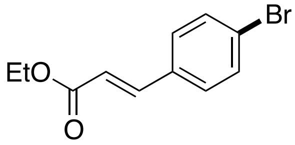

5s

^{1}H NMR (500 MHz, $CDCl_3$): δ 8.20 (d, *J* = 2.0 Hz, 1H), 8.04 (d, *J* = 3.0 Hz, 1H), 7.61 (d, *J* = 16.0 Hz, 1H), 7.52 (d, *J* = 8.0 Hz, 2H), 7.38 (d, *J* = 8.0 Hz, 2H), 6.42 (d, *J* = 16.0 Hz, 1H), 4.26 (q, *J* = 7.5 Hz, 2H), 1.33 (t, *J* = 7.5 Hz, 3H).
^{13}C NMR (125 MHz, $CDCl_3$): δ 166.7, 143.2, 133.4, 132.1, 129.4, 124.4, 119.0, 60.6, 14.3.

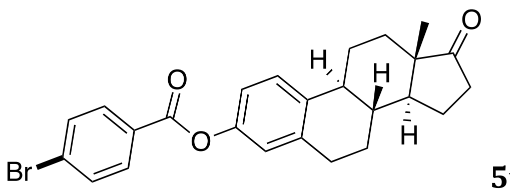

5t

^{1}H NMR (400 MHz, CDCl$_3$): δ 7.94 (dd, *J* = 2.0, 6.4 Hz, 2H), 7.53 (dd, *J* = 2.0, 6.4 Hz, 2H), 7.23 (d, *J* = 8.4 Hz, 1H), 6.88 (dd, *J* = 2.8, 8.4 Hz, 1H), 6.84 (d, *J* = 2.8 Hz, 1H), 2.83 (dd, *J* = 4.4 Hz, 2H), 2.42 (d, *J* = 8.4 Hz, 1H), 2.32 (m, 2H), 2.20 (m, 1H), 1.97 (m, 4H), 1.41 (m, 6H), 0.83 (s, 3H).

^{13}C NMR (100 MHz, CDCl$_3$): δ 220.5, 164.5, 148.5, 138.0, 137.5, 131.7, 131.5, 131.4, 128.5, 126.4, 121.4, 118.6, 50.4, 47.9, 44.2, 38.0, 35.9, 31.5, 29.4, 26.3, 25.7, 21.6, 13.8.

HRMS: m/z (ESI) Calculated for [M+H]$^+$ 453.1065, Found 453.1061.

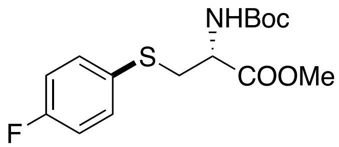

7a

^{1}H NMR (400 MHz, CDCl$_3$): δ 7.39 (dd, *J* = 5.6, 9.2 Hz, 2H), 6.96 (t, *J* = 8.8 Hz, 2H), 5.35 (d, *J* = 7.6 Hz, 1H), 4.50 (m, 1H), 3.53 (s, 3H), 3.27 (m, 2H), 1.38 (s, 9H).

^{13}C NMR (100 MHz, CDCl$_3$): δ 170.9, 162.1 (d, *J* = 246.0 Hz), 154.8, 133.9 (dd, *J* = 8.0, 13.2 Hz), 129.6, 116.0 (d, *J* = 21.7 Hz), 80.0, 53.2, 52.2 (d, *J* = 12.2 Hz), 38.1, 28.1 (d, *J* = 6.8 Hz).

HRMS: m/z (ESI) Calculated for [M+Na]$^+$ 352.0995, Found 352.0983.

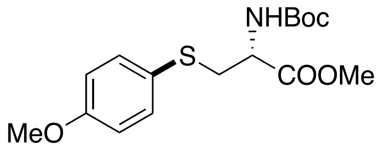

7b

^{1}H NMR (400 MHz, CDCl$_3$): δ 7.39 (dt, *J* = 2.0, 7.2 Hz, 2H), 6.83 (dt, *J* = 2.0, 7.2 Hz, 2H), 5.35 (d, *J* = 6.0 Hz, 1H), 4.50 (m, 1H), 3.78 (s, 3H), 3.54 (s, 3H), 3.25 (d, *J* = 4.0 Hz, 2H), 1.42 (s, 9H).

^{13}C NMR (125 MHz, CDCl$_3$): δ 171.1, 159.4, 154.9, 134.4, 124.7, 114.6, 79.9, 55.2, 53.1, 52.2, 38.7, 28.2.

HRMS: m/z (ESI) Calculated for [M+Na]$^+$ 364.1195, Found 364.1185.

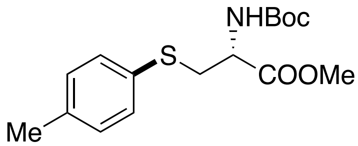

7c

^{1}H NMR (500 MHz, CDCl$_3$): δ 7.31 (d, *J* = 8.0 Hz, 2H), 7.09 (d, *J* = 8.0, Hz, 2H), 5.37 (d, *J* = 7.0 Hz, 1H), 4.53 (m, 1H), 3.54 (s, 3H), 3.31 (d, *J* = 5.0 Hz, 2H), 2.31 (s, 3H), 1.41 (s, 9H).

^{13}C NMR (125 MHz, $CDCl_3$): δ 171.0, 154.9, 137.1, 131.6, 130.8, 129.7, 79.9, 53.2, 52.2, 37.7, 28.1, 20.9.
HRMS: m/z (ESI) Calculated for $[M+Na]^+$ 348.1245, Found 348.1258.

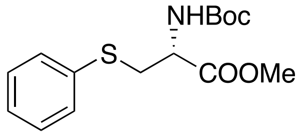

7d

^{1}H NMR (500 MHz, $CDCl_3$): δ 7.41 (d, J = 8.0 Hz, 2H), 7.28 (t, J = 8.0 Hz, 2H), 7.21 (t, J = 7.5 Hz, 1H), 5.39 (d, J = 7.0 Hz, 1H), 4.56 (m, 1H), 3.53 (s, 3H), 3.37 (m, 2H), 1.42 (s, 9H).
^{13}C NMR (125 MHz, $CDCl_3$): δ 171.0, 155.0, 134.7, 131.0, 129.0, 127.0, 80.1, 53.2, 52.3, 37.2, 28.2.
HRMS: m/z (ESI) Calculated for $[M+Na]^+$ 334.1089, Found 334.1097.

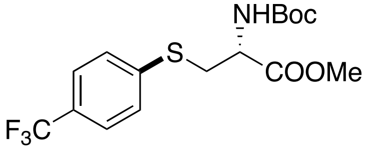

7e

^{1}H NMR (400 MHz, $CDCl_3$): δ 7.52 (d, J = 8.4 Hz, 2H), 7.46 (d, J = 8.4 Hz, 2H), 5.34 (d, J = 6.4 Hz, 1H), 4.56 (m, 1H), 3.59 (s, 3H), 3.35 (m, 2H), 1.41 (s, 9H).
^{13}C NMR (125 MHz, $CDCl_3$): δ 170.7, 154.9, 140.5, 129.3, 128.5 (q, J = 26.2 Hz), 125.7, (d, J = 2.7 Hz), 124.0 (q, J = 216.3 Hz), 80.3, 53.3, 52.5, 36.0, 28.2.
HRMS: m/z (ESI) Calculated for $[M+Na]^+$ 402.0963, Found 402.0970.

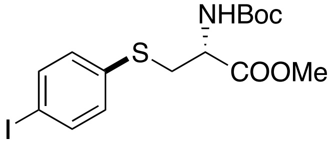

7f

^{1}H NMR (400 MHz, $CDCl_3$): δ 7.60 (dd, J = 2.0, 6.8 Hz, 2H), 7.14 (dt, J = 1.6, 6.8 Hz, 2H), 5.29 (d, J = 8.0 Hz, 1H), 4.57 (m, 1H), 3.60 (s, 3H), 3.37 (m, 2H), 1.42 (s, 9H).
^{13}C NMR (125 MHz, $CDCl_3$): δ 170.8, 154.8, 138.0, 132.5, 92.1, 80.2, 53.3, 52.5, 37.0, 28.2.
HRMS: m/z (ESI) Calculated for $[M+Na]^+$ 460.0055, Found 460.0063.

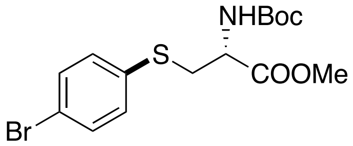

7g

^{1}H NMR (500 MHz, $CDCl_3$): δ 7.40 (dt, *J* = 2.5, 9.0 Hz, 2H), 7.27 (dt, *J* = 2.0, 8.5 Hz, 2H), 5.35 (d, *J* = 7.5 Hz, 1H), 4.56 (m, 1H), 3.59 (s, 3H), 3.35 (m, 2H), 1.41 (s, 9H).
^{13}C NMR (125 MHz, $CDCl_3$): δ 170.8, 154.8, 134.0, 132.4, 132.0, 120.9, 80.1, 53.2, 52.4, 37.1, 28.2.
HRMS: m/z (ESI) Calculated for $[M+Na]^+$ 412.0194, Found 12.0189.

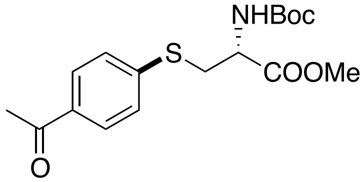

7h

^{1}H NMR (500 MHz, $CDCl_3$): δ 7.87 (d, *J* = 8.5 Hz, 2H), 7.40 (d, *J* = 8.5 Hz, 2H), 5.32 (d, *J* = 7.5 Hz, 1H), 4.57 (m, 1H), 3.56 (s, 3H), 3.37 (m, 2H), 2.57 (s, 3H), 1.41 (s, 9H).
^{13}C NMR (125 MHz, $CDCl_3$): δ 197.0, 170.7, 154.9, 142.4, 134.7, 128.8, 128.1, 80.3, 53.2, 52.6, 35.4, 28.2, 26.5.
HRMS: m/z (ESI) Calculated for $[M+Na]^+$ 376.1195, Found 376.1198.

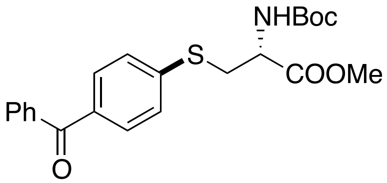

7i

^{1}H NMR (400 MHz, $CDCl_3$): δ 7.76 (d, *J* = 7.6 Hz, 2H), 7.73 (d, *J* = 8.4 Hz, 2H), 7.59 (t, *J* = 7.6 Hz, 1H), 7.49 (d, *J* = 7.6 Hz, 2H), 7.43 (d, *J* = 8.4 Hz, 2H), 5.41 (d, *J* = 6.8 Hz, 1H), 4.65 (m, 1H), 3.66 (s, 3H), 3.47 (m, 2H), 1.43 (s, 9H).
^{13}C NMR (125 MHz, $CDCl_3$): δ 195.6, 170.8, 154.9, 141.6, 137.5, 135.0, 132.4, 130.6, 129.8, 128.3, 127.9, 80.3, 53.2, 52.5, 35.4, 28.2.
HRMS: m/z (ESI) Calculated for $[M+Na]^+$ 438.1351, Found 438.1350.

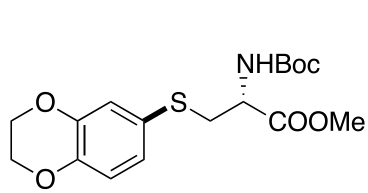

7j

^{1}H NMR (400 MHz, $CDCl_3$): δ 6.97 (d, *J* = 2.0 Hz, 1H), 6.92 (dd, *J* = 2.0, 6.4 Hz, 1H), 6.78 (d, *J* = 6.8 Hz, 1H), 5.34 (d, *J* = 5.6 Hz, 1H), 4.51 (m, 1H), 4.23 (s, 4H), 3.61 (s, 3H), 3.26 (m, 2H), 1.42 (s, 9H).
^{13}C NMR (125 MHz, $CDCl_3$): δ 171.1, 154.9, 143.6, 143.4, 125.8, 121.4, 117.8, 79.9, 64.3, 64.2, 53.2, 52.3, 38.4, 28.2.
HRMS: m/z (ESI) Calculated for $[M+Na]^+$ 392.1144, Found 392.1130.

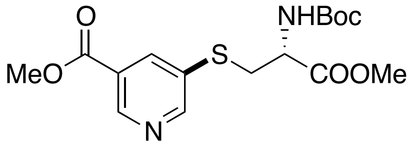

7k

^{1}H NMR (500 MHz, $CDCl_3$): δ 9.05 (s, 1H), 8.76 (d, *J* = 1.5 Hz, 1H), 8.31 (s, 1H), 5.39 (d, *J* = 7.5 Hz, 1H), 4.60 (m, 1H), 3.96 (s, 3H), 3.64 (s, 3H), 3.46 (m, 2H), 1.41 (s, 9H).
^{13}C NMR (125 MHz, $CDCl_3$): δ 170.6, 165.1, 154.6, 148.7, 138.6, 132.7, 126.1, 80.4, 53.2, 52.6, 36.9, 28.2.
HRMS: m/z (ESI) Calculated for $[M+Na]^+$ 393.1096, Found 393.1074.

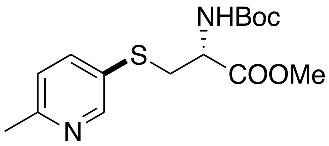

7l

^{1}H NMR (400 MHz, $CDCl_3$): δ 8.53 (s, 1H), 7.65 (dd, *J* = 1.6, 7.6 Hz, 1H), 7.10 (d, *J* = 8.4 Hz, 1H), 5.38 (d, *J* = 6.8 Hz, 1H), 4.53 (m, 1H), 3.60 (s, 3H), 3.33 (m, 2H), 2.53 (s, 3H), 1.41 (s, 9H).
^{13}C NMR (125 MHz, $CDCl_3$): δ 170.6, 157.4, 154.8, 151.8, 139.8, 128.2, 123.4, 80.1, 53.2, 37.8, 28.2, 23.9.
HRMS: m/z (ESI) Calculated for $[M+Na]^+$ 349.1198, Found 349.1187.

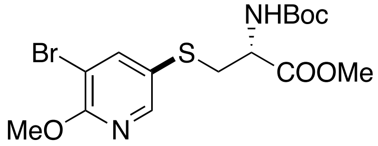

7m

^{1}H NMR (400 MHz, $CDCl_3$): δ 8.17 (dd, *J* = 1.6, 7.6 Hz, 1H), 7.10 (d, *J* = 8.4 Hz, 1H), 5.38 (d, *J* = 6.8 Hz, 1H), 4.53 (m, 1H), 3.60 (s, 3H), 3.33 (m, 2H), 2.53 (s, 3H), 1.41 (s, 9H).
^{13}C NMR (125 MHz, $CDCl_3$): δ 170.8, 159.8, 154.8, 149.6, 145.9, 123.9, 106.9, 80.3, 54.7, 53.3, 52.5, 38.9, 28.2.
HRMS: m/z (ESI) Calculated for $[M+Na]^+$ 443.0252, Found 443.0250.

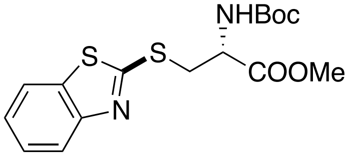

7n

^{1}H NMR (500 MHz, $CDCl_3$): δ 7.87 (d, *J* = 8.0 Hz, 1H), 7.75 (d, *J* = 8.0 Hz, 1H), 7.42 (t, *J* = 7.5 Hz, 1H), 7.31 (t, *J* = 7.5 Hz, 1H), 6.19 (d, *J* = 7.5 Hz, 1H), 4.75 (dd, *J* = 5.5, 12.0 Hz, 1H), 3.82 (d, J = 5.0 Hz, 2H), 3.73 (s, 3H), 1.41 (s, 9H).
^{13}C NMR (125 MHz, $CDCl_3$): δ 170.9, 152.7, 135.5, 127.1, 126.2, 124.6, 121.6, 121.0, 111.8, 80.0, 53.9, 52.7, 35.4, 28.2.
HRMS: m/z (ESI) Calculated for $[M+Na]^+$ 391.0762, Found 391.0743.

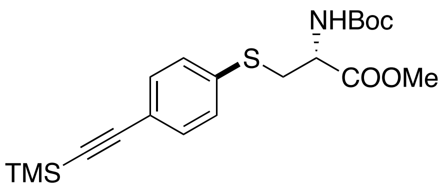

7o

^{1}H NMR (500 MHz, $CDCl_3$): δ 7.35 (d, *J* = 8.5 Hz, 2H), 7.29 (d, *J* = 8.0 Hz, 2H), 5.32 (d, *J* = 7.5 Hz, 1H), 4.57 (m, 1H), 3.56 (s, 3H), 3.37 (m, 2H), 1.41 (s, 9H), 0.23 (s, 9H).
^{13}C NMR (125 MHz, $CDCl_3$): δ 170.8, 154.9, 135.8, 132.3, 129.8, 121.5, 104.4, 95.1, 80.2, 53.2, 52.4, 36.6, 28.2, -0.1.
HRMS: m/z (ESI) Calculated for $[M+Na]^+$ 430.1484, Found 430.1481.

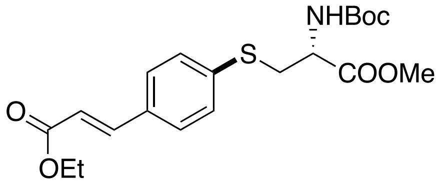

7p

^{1}H NMR (500 MHz, $CDCl_3$): δ 7.68 (d, *J* = 16.5 Hz, 1H), 7.58 (s, 4H), 6.47 (d, *J* = 16.5 Hz, 1H), 5.53 (d, *J* = 7.5 Hz, 1H), 4.76 (m, 1H), 4.27 (q, *J* = 7.0 Hz, 2H), 3.96 (m, 2H), 3.73 (s, 3H), 1.42 (s, 9H), 1.34 (t, *J* = 7.5 Hz, 3H).
^{13}C NMR (125 MHz, $CDCl_3$): δ 170.9, 166.6, 154.9, 152.1, 143.2, 135.8, 128.8, 122.0, 119.4, 80.1, 60.6, 53.2, 52.6, 36.7, 28.2, 14.2.
HRMS: m/z (ESI) Calculated for $[M+Na]^+$ 432.1457, Found 432.1416.

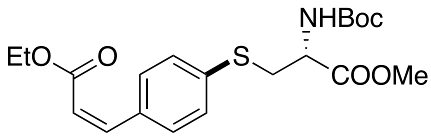

(*Z*)-7q

^{1}H NMR (500 MHz, $CDCl_3$): δ 7.62 (d, *J* = 15.5 Hz, 1H), 7.43 (d, *J* = 8.5 Hz, 2H), 7.37 (d, *J* = 8.0 Hz, 2H), 6.40 (d, *J* = 15.5 Hz, 1H), 5.38 (d, *J* = 7.5 Hz, 1H), 4.60 (m, 1H), 4.26 (q, *J* = 7.0 Hz, 2H), 3.60 (s, 3H), 3.41 (m, 2H), 1.41 (s, 9H), 1.34 (t, *J* = 7.0 Hz, 3H).
^{13}C NMR (125 MHz, $CDCl_3$): δ 170.7, 166.8, 154.8, 152.1, 143.5, 132.6, 129.8, 128.4, 118.1, 80.0, 60.5, 53.2, 52.4, 36.2, 28.2, 14.2.
HRMS: m/z (ESI) Calculated for $[M+Na]^+$ 432.1457, Found 432.1416.

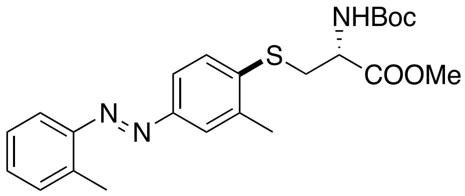

(*E*)-7q

^{1}H NMR (400 MHz, $CDCl_3$): δ 7.73 (s, 1H), 7.72 (d, *J* = 8.0 Hz, 1H), 7.60 (d, *J* = 8.4 Hz, 1H), 7.46 (d, *J* = 8.0 Hz, 1H), 7.33 (m, 2H), 7.24 (m, 1H), 5.38 (d, *J* = 7.6 Hz, 1H), 4.65 (m, 1H), 3.63 (s, 3H), 3.45 (m, 2H), 2.71 (s, 3H), 2.47 (s, 3H), 1.43 (s, 9H).
^{13}C NMR (100 MHz, $CDCl_3$): δ 170.9, 154.9, 151.2, 150.7, 138.7, 138.0, 131.2, 130.8, 129.2, 129.1, 126.3, 124.4, 121.0, 115.3, 80.2, 53.2, 52.5, 35.8, 29.6, 28.2, 20.6, 17.4.
HRMS: m/z (ESI) Calculated for $[M+Na]^+$ 466.1776, Found 446.1752.

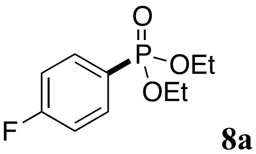

8a

^{1}H NMR (400 MHz, $CDCl_3$): δ 7.83 (ddd, *J* = 5.6, 8.4, 13.6 Hz, 2H), 7.15 (ddd, *J* = 3.2, 9.2 Hz, 2H), 4.12 (m, 4H), 1.33 (t, *J* = 7.2 Hz, 6H).
^{13}C NMR (100 MHz, $CDCl_3$): δ 165.3 (d, *J* = 199.3 Hz), 134.3 (t, *J* = 9.1 Hz), 124.4 (d, *J* = 150.7 Hz), 115.8 (dd, *J* = 12.9, 16.6 Hz), 62.1 (d, *J* = 3.8 Hz), 16.2 (d, *J* = 4.9 Hz).

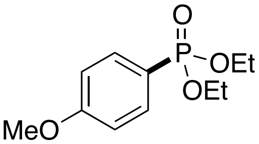

8b

^{1}H NMR (400 MHz, $CDCl_3$): δ 7.75 (dd, *J* = 8.8, 12.4 Hz, 2H), 6.97 (dd, *J* = 3.6, 8.8 Hz, 2H), 4.09 (m, 4H), 3.85 (s, 3H), 1.31 (t, *J* = 6.8 Hz, 6H).
^{13}C NMR (125 MHz, $CDCl_3$): δ 162.8, 133.8 (d, *J* = 11.4 Hz), 119.5 (d, *J* = 193.6 Hz), 114.0 (d, *J* = 15.6 Hz), 61.9 (d, *J* = 5.2 Hz), 53.3, 16.3 (d, *J* = 6.6 Hz).
HRMS: m/z (ESI) Calculated for $[M+H]^+$ 245.0943, Found 245.0931.

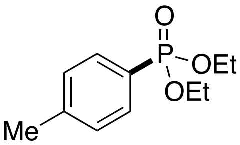

8c

^{1}H NMR (400 MHz, $CDCl_3$): δ 7.70 (dd, *J* = 8.0, 13.0 Hz, 2H), 7.27 (dd, *J* = 3.5, 8.0 Hz, 2H), 4.10 (m, 4H), 2.40 (s, 3H), 1.31 (t, *J* = 7.0 Hz, 6H).
^{13}C NMR (100 MHz, $CDCl_3$): δ 142.9 (d, *J* = 2.3 Hz), 131.8 (t, *J* = 7.9 Hz), 129.2 (d, *J* = 12.1 Hz), 124.9 (d, *J* = 155.0 Hz), 61.9 (d, *J* = 4.2 Hz), 21.6, 16.3 (d, *J* = 5.3 Hz).

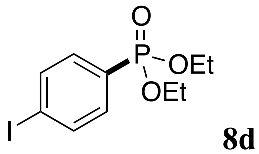

8d

^{1}H NMR (400 MHz, $CDCl_3$): δ 7.83 (dd, *J* = 3.6, 8.4 Hz, 2H), 7.52 (dd, *J* = 8.0, 12.8 Hz, 2H), 4.11 (m, 4H), 1.32 (t, *J* = 7.2 Hz, 6H).
^{13}C NMR (125 MHz, $CDCl_3$): δ 137.7 (d, *J* = 15.2 Hz), 133.1 (d, *J* = 10.4 Hz), 128.0 (d, *J* = 188.8 Hz), 100.1 (d, *J* = 3.9 Hz), 62.3 (d, *J* = 5.3 Hz), 16.3 (d, *J* = 6.2 Hz).

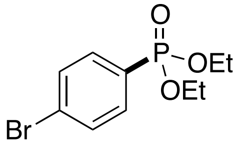

8e

^{1}H NMR (400 MHz, $CDCl_3$): δ 765 (m, 4H), 4.12 (m, 4H), 1.32 (t, *J* = 7.2 Hz, 6H).
^{13}C NMR (100 MHz, $CDCl_3$): δ 133.2 (d, *J* = 8.3 Hz), 131.7 (d, *J* = 12.1 Hz), 127.5 (d, *J* = 3.1 Hz), 127.4 (d, *J* = 151.9 Hz), 62.2 (d, *J* = 4.2 Hz), 16.2 (d, *J* = 5.4 Hz).

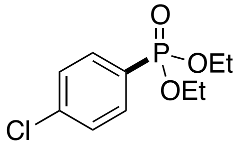

8f

^{1}H NMR (400 MHz, $CDCl_3$): δ 7.75 (dd, *J* = 8.4, 13.2 Hz, 2H), 7.45 (dd, *J* = 3.6, 8.4 Hz, 2H), 4.11 (m, 4H), 1.32 (t, *J* = 7.2 Hz, 6H).
^{13}C NMR (100 MHz, $CDCl_3$): δ 138.9 (d, *J* = 3.0 Hz), 133.1 (d, *J* = 8.7 Hz), 128.8 (d, *J* = 12.5 Hz), 126.9 (d, *J* = 151.8 Hz), 62.2 (d, *J* = 4.2 Hz), 16.2 (d, *J* = 5.4 Hz).
HRMS: m/z (ESI) Calculated for $[M+H]^+$ 249.0447, Found 249.0439.

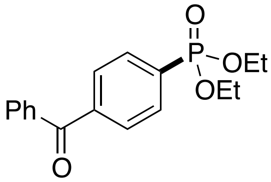

8g

^{1}H NMR (500 MHz, $CDCl_3$): δ 7.94 (dd, *J* = 8.0, 12.8 Hz, 1H), 7.85 (m, 1H), 7.81 (m, 3H), 7.63 (m, 2H), 7.51 (m ,2H), 4.15 (m, 4H), 1.36 (t, *J* = 7.2 Hz, 6H).

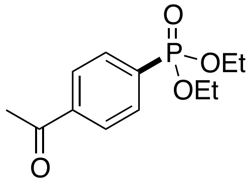

8h

^{1}H NMR (400 MHz, $CDCl_3$): δ 7.75 (dd, *J* = 8.4, 13.2 Hz, 2H), 7.45 (dd, *J* = 3.6, 8.4 Hz, 2H), 4.11 (m, 4H), 1.32 (t, *J* = 7.2 Hz, 6H).
^{13}C NMR (125 MHz, $CDCl_3$): δ 138.9 (d, *J* = 3.0 Hz), 133.1 (d, *J* = 8.7 Hz), 128.8 (d, *J* = 12.5 Hz), 126.9 (d, *J* = 151.8 Hz), 62.2 (d, *J* = 4.2 Hz), 16.2 (d, *J* = 5.4 Hz).

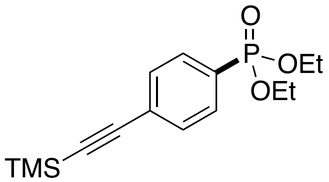

8i

^{1}H NMR (500 MHz, $CDCl_3$): δ 7.74 (dd, J = 8.5, 13.5 Hz, 2H), 7.54 (dd, J = 3.5, 8.5 Hz, 2H), 4.10 (m, 4H), 1.32 (t, J = 7.2 Hz, 6H), 0.25 (s, 9H).
^{13}C NMR (125 MHz, $CDCl_3$): δ 131.8 (d, J = 14.6 Hz), 131.5 (d, J = 9.9 Hz), 128.2 (d, J = 188.3 Hz), 127.3 (d, J = 3.3 Hz), 103.8, 97.4, 62.2 (d, J = 5.3 Hz), 16.3 (d, J = 6.7 Hz), -0.2.
HRMS: m/z (ESI) Calculated for $[M+H]^+$ 311.1232, Found 311.1220.

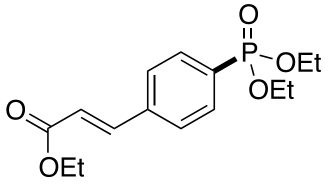

8j

^{1}H NMR (400 MHz, $CDCl_3$): δ 7.83 (dd, J = 8.0, 12.8 Hz, 2H), 7.78 (d, J = 16.0 Hz, 1H), 7.60 (dd, J = 3.6, 8.0 Hz, 2H), 6.51 (d, J = 16.0 Hz, 1H), 4.28 (q, J = 7.2 Hz, 2H), 4.13 (m, 4H), 1.35 (t, J = 7.2 Hz, 3H), 1.33 (t, J = 7.2 Hz, 6H).
^{13}C NMR (125 MHz, $CDCl_3$): δ 166.5, 143.1, 138.2 (d, J = 3.3 Hz), 132.3 (d, J = 10.0 Hz), 127.8 (d, J = 15.2 Hz), 120.7, 130.1 (d, J = 187.9 Hz), 62.3 (d, J = 5.6 Hz), 60.8, 16.3 (d, J = 6.6 Hz), 14.3.
HRMS: m/z (ESI) Calculated for $[M+H]^+$ 313.1205, Found 313.1200.

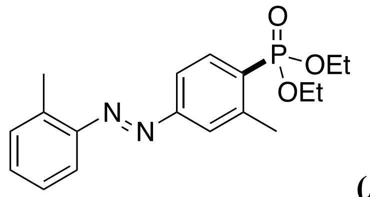

(*E*)-8k

^{1}H NMR (500 MHz, $CDCl_3$): δ 8.07 (dd, J = 8.5, 13.5 Hz, 1H), 7.76 (m, 2H), 7.76 (d, J = 8.0 Hz, 1H), 7.38 (m, 2H), 7.27 (dt, J = 2.0, 8.0 Hz, 1H), 4.16 (m, 4H), 2.74 (s, 3H), 2.69 (s, 3H), 1.36 (t, J = 6.5 Hz, 6H).
^{13}C NMR (125 MHz, $CDCl_3$): δ 155.0 (d, J = 4.3 Hz), 150.7, 143.1, 138.7, 135.0 (d, J = 13.6 Hz), 130.2 (d, J = 188.6 Hz), 131.4, 126.4, 125.4 (d, J = 19.5 Hz), 119.4 (d, J = 19.0 Hz), 115.4, 62.0 (d, J = 6.6 Hz), 21.3(d, J = 4.2 Hz), 17.6, 16.3 (d, J = 7.8 Hz).
HRMS: m/z (ESI) Calculated for $[M+H]^+$ 347.1525, Found 347.1510.

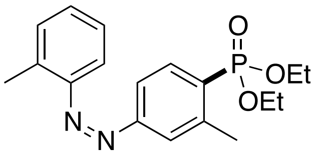

(*Z*)-8k

^{1}H NMR (500 MHz, $CDCl_3$): δ 7.72 (dd, *J* = 8.0, 13.5 Hz, 1H), 7.18 (d, *J* = 8.0 Hz, 1H), 7.06 (t, *J* = 7.5 Hz, 1H), 6.91 (t, *J* = 8.0 Hz, 1H), 6.83 (dd, *J* = 1.5, 5.0 Hz, 1H), 6.54 (dt, *J* = 2.5, 7.5 Hz, 1H), 6.19 (d, *J* = 8.0 Hz, 1H), 4.09 (m, 4H), 2.49 (s, 3H), 2.33 (s, 3H), 1.30 (t, *J* = 6.5 Hz, 6H).

^{13}C NMR (125 MHz, $CDCl_3$): δ 156.5 (d, *J* = 4.3 Hz), 152.8, 143.2, 141.4, 134.3 (d, *J* = 13.6 Hz), 131.1 (d, *J* = 188.6 Hz), 128.2, 127.7, 125.9, 123.1 (d, *J* = 19.0 Hz), 117.1, 115.8 (d, *J* = 19.5 Hz), 62.0 (d, *J* = 6.6 Hz), 21.2(d, *J* = 4.2 Hz), 17.5, 16.2 (d, *J* = 7.8 Hz).

HRMS: m/z (ESI) Calculated for $[M+H]^+$ 347.1525, Found 347.1510.

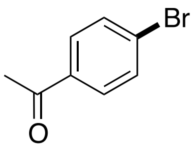
Br
O

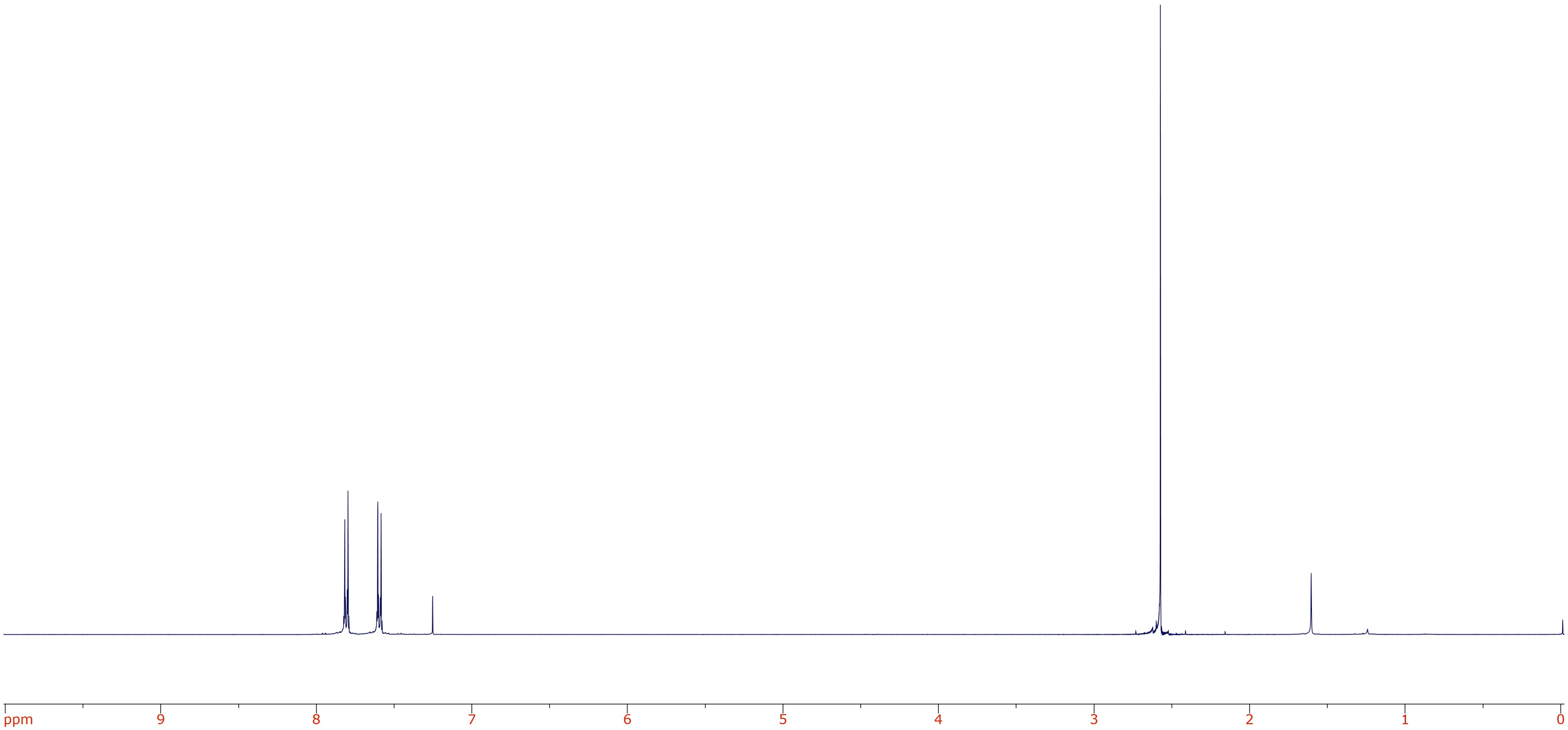
ppm
9
8
7
6
5
4
3
2
1
0

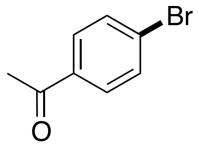
Br
O

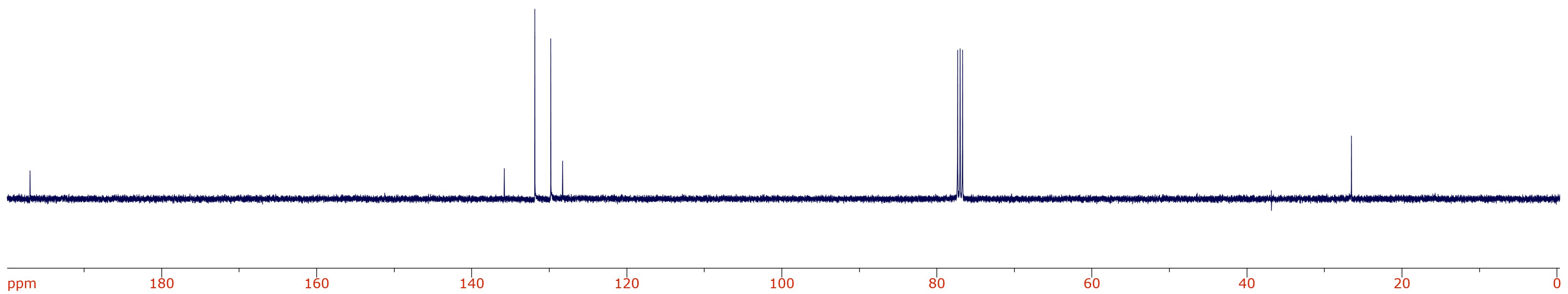
ppm
180
160
140
120
100
80
60
40
20
0

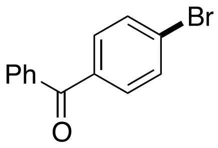
Ph
O
Br

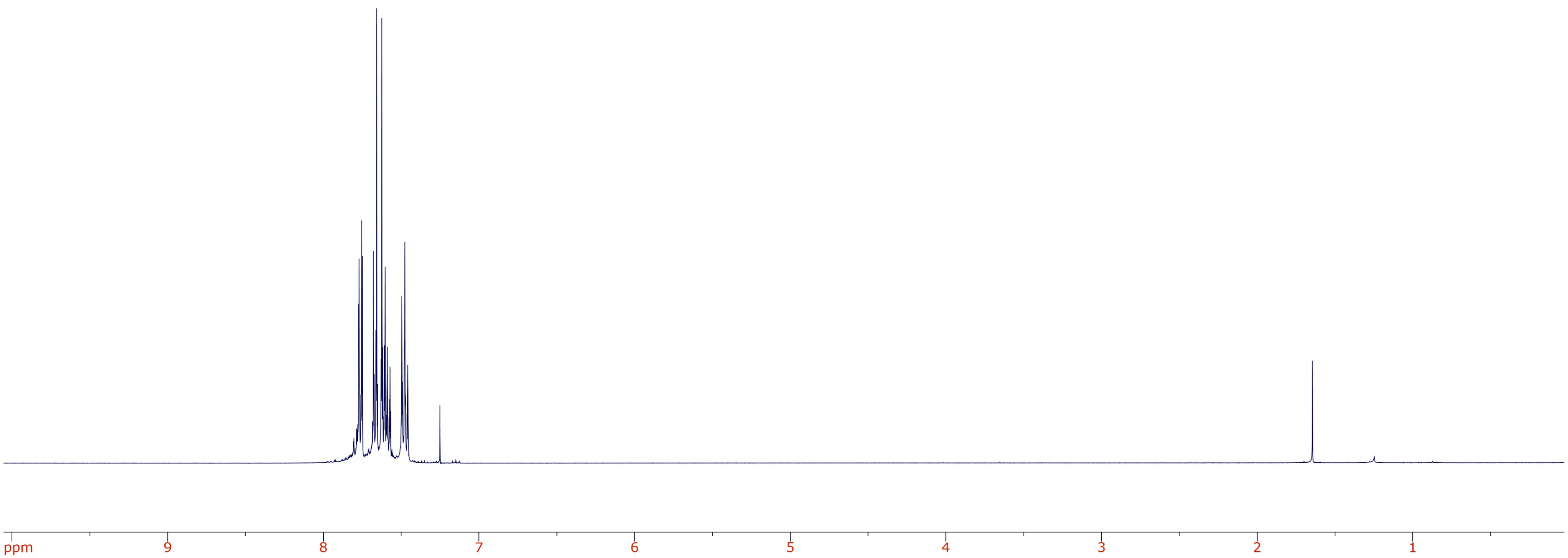
ppm
9
8
7
6
5
4
3
2
1

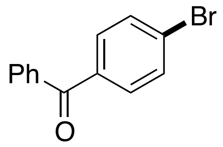
Br
Ph
O

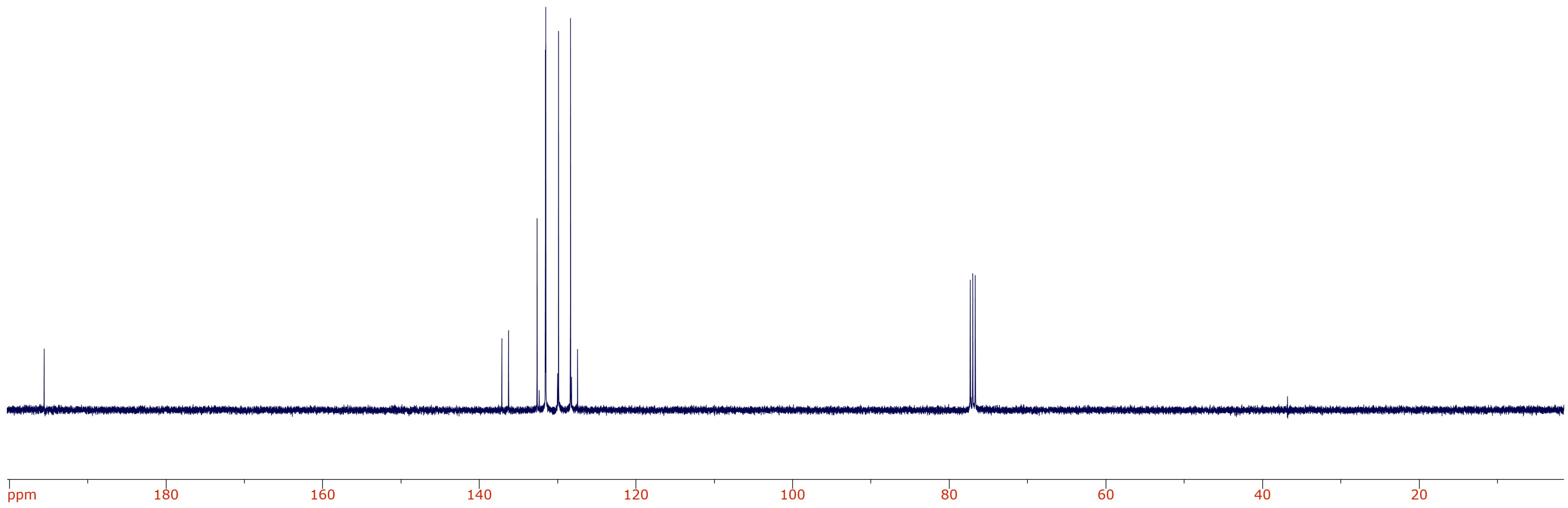
ppm
180
160
140
120
100
80
60
40
20

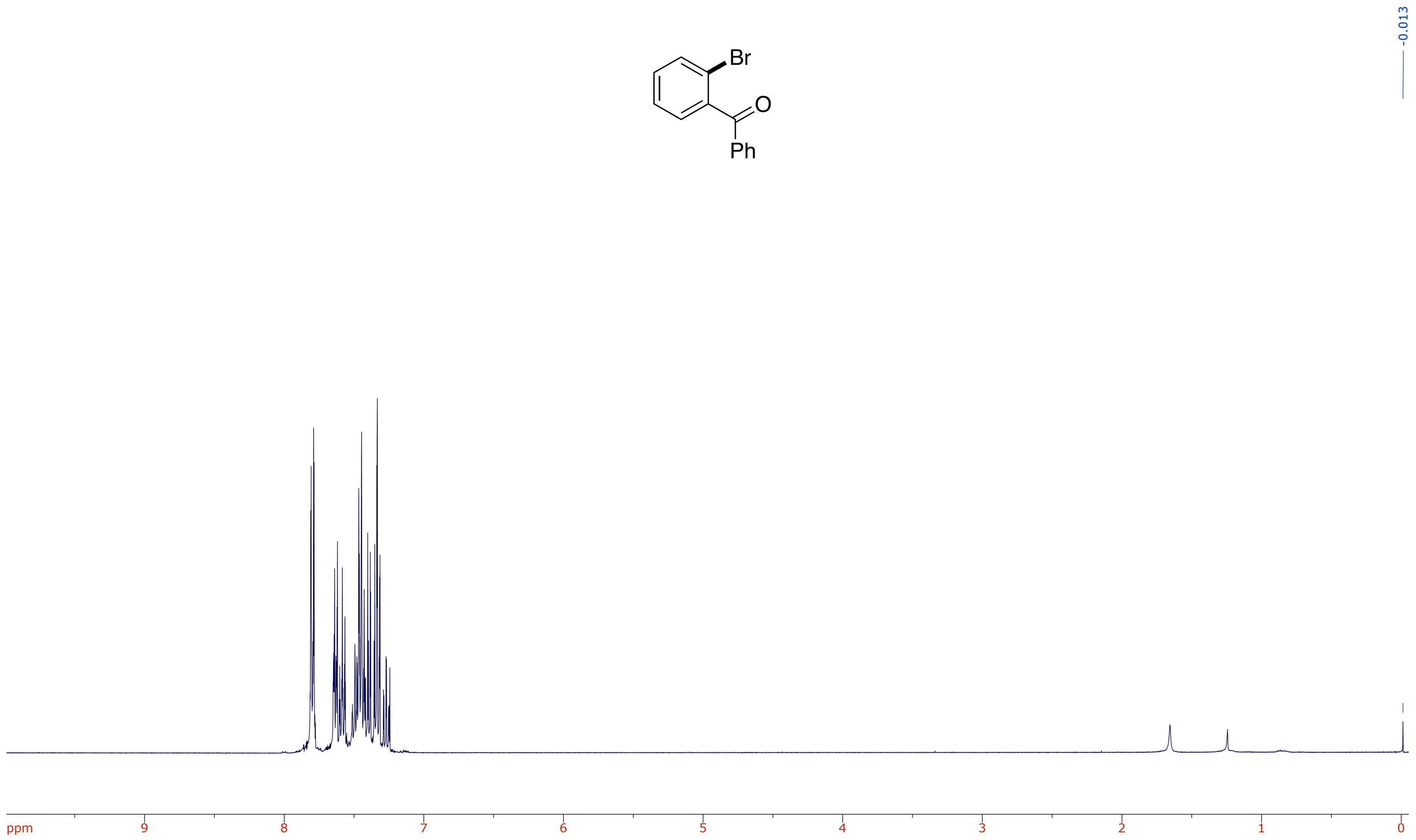
Br
O
Ph
-0.013
ppm
9
8
7
6
5
4
3
2
1
0

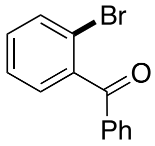

Br
O
Ph

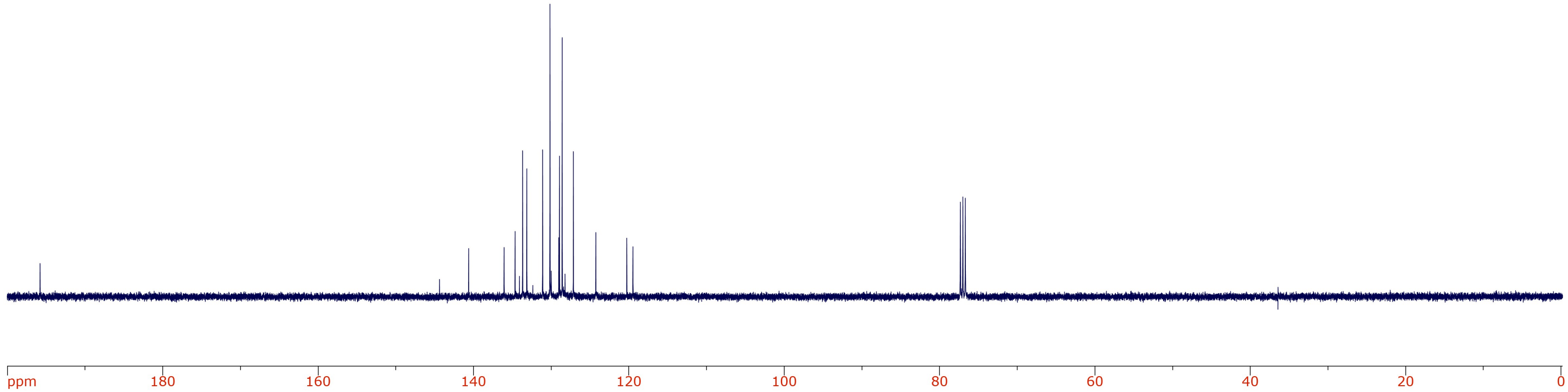

ppm
180
160
140
120
100
80
60
40
20
0

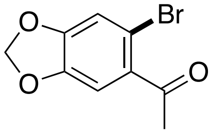
Br
O
O
O

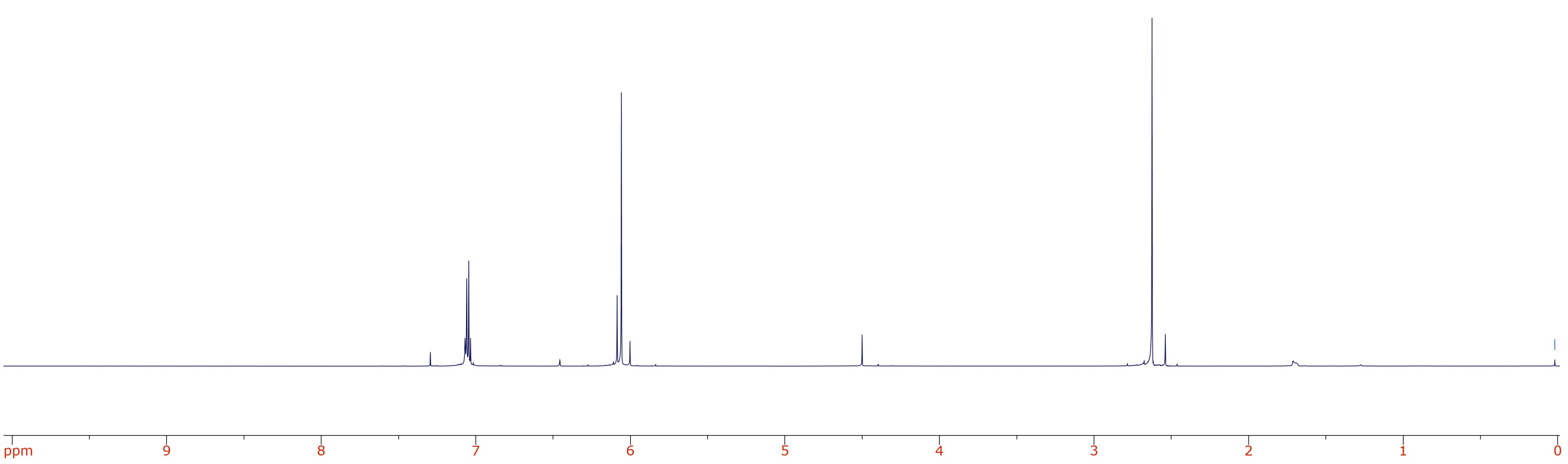
0.019
ppm
9
8
7
6
5
4
3
2
1
0

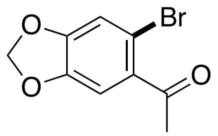
O
O
Br
O

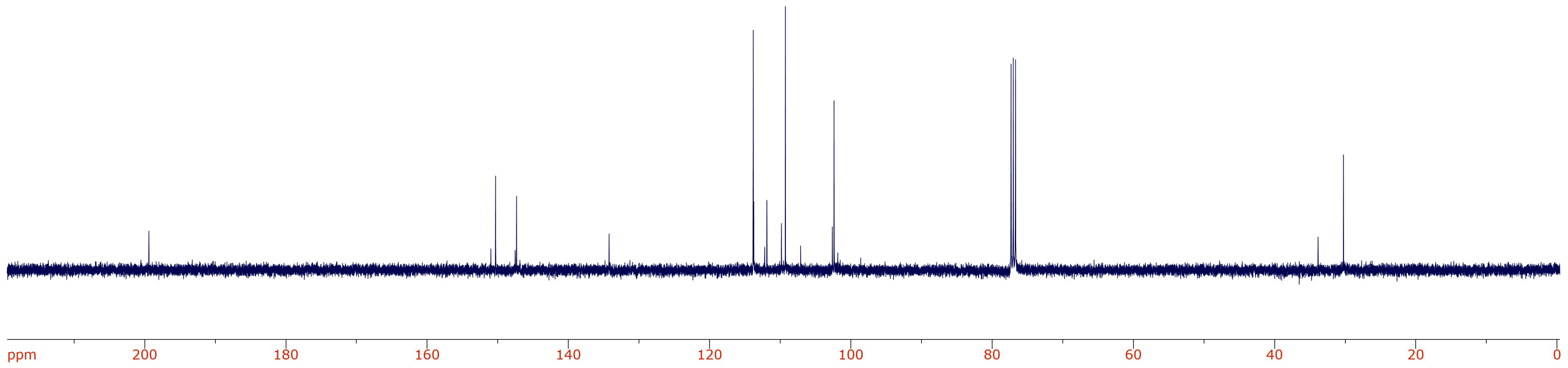
ppm
200
180
160
140
120
100
80
60
40
20
0

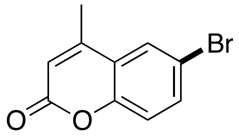
Br
O
O

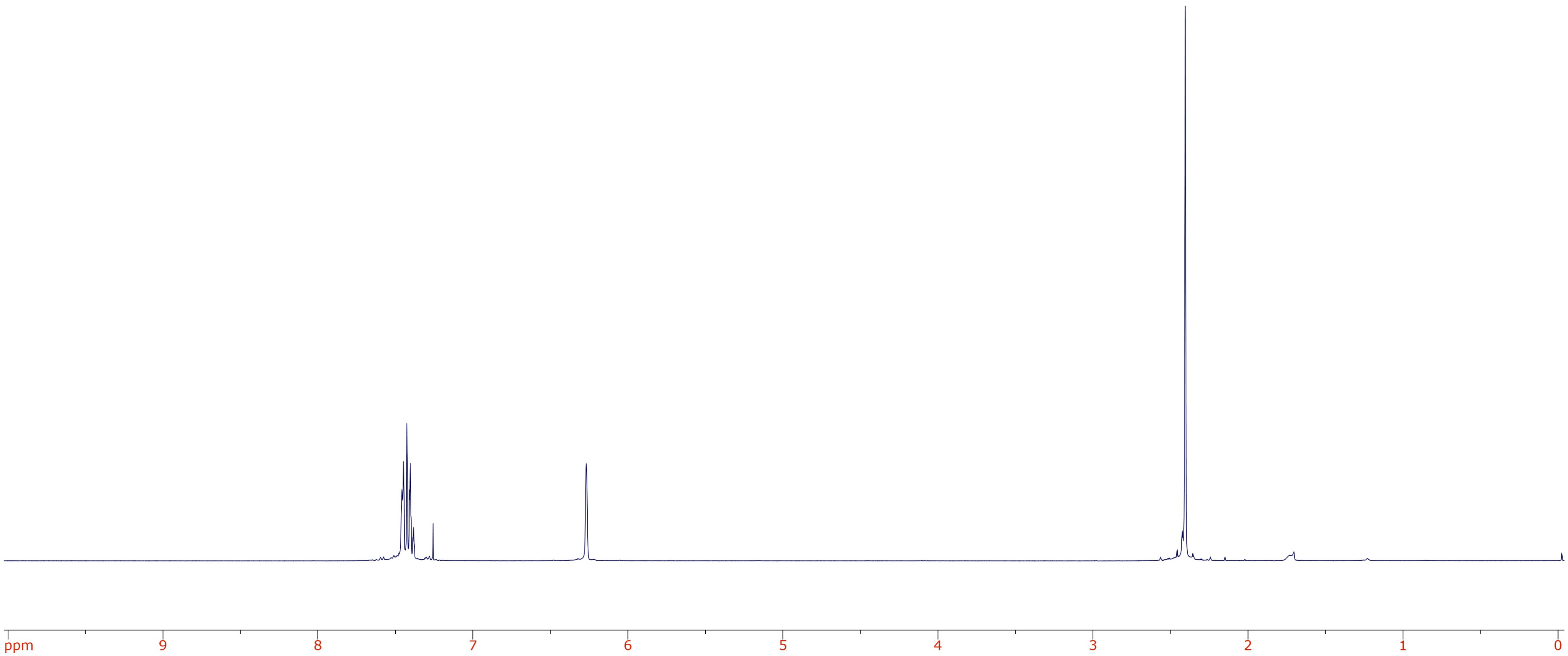
ppm
9
8
7
6
5
4
3
2
1
0

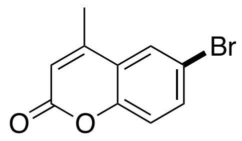
Br
O
O

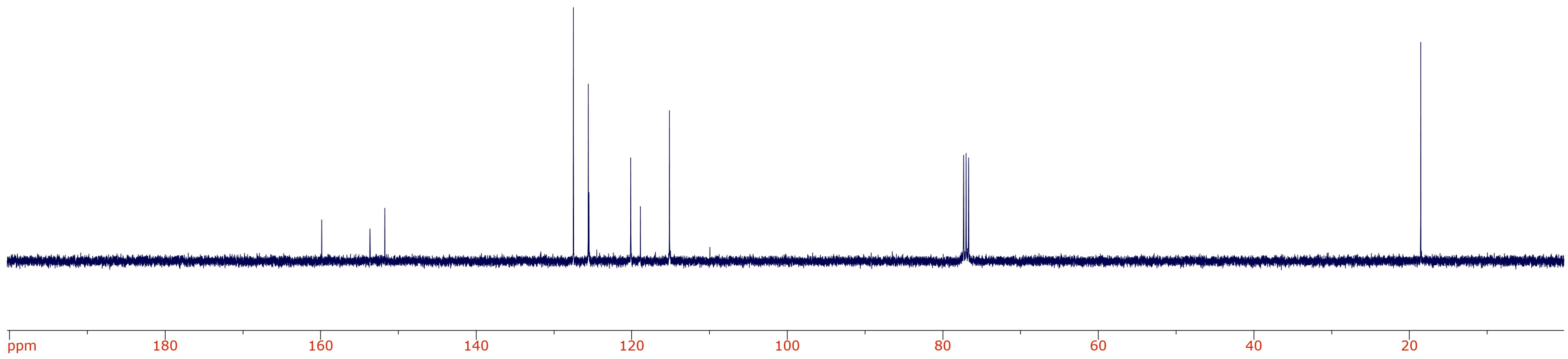
ppm
180
160
140
120
100
80
60
40
20

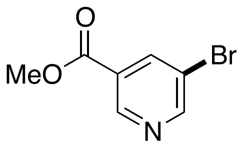
O
MeO
Br
N

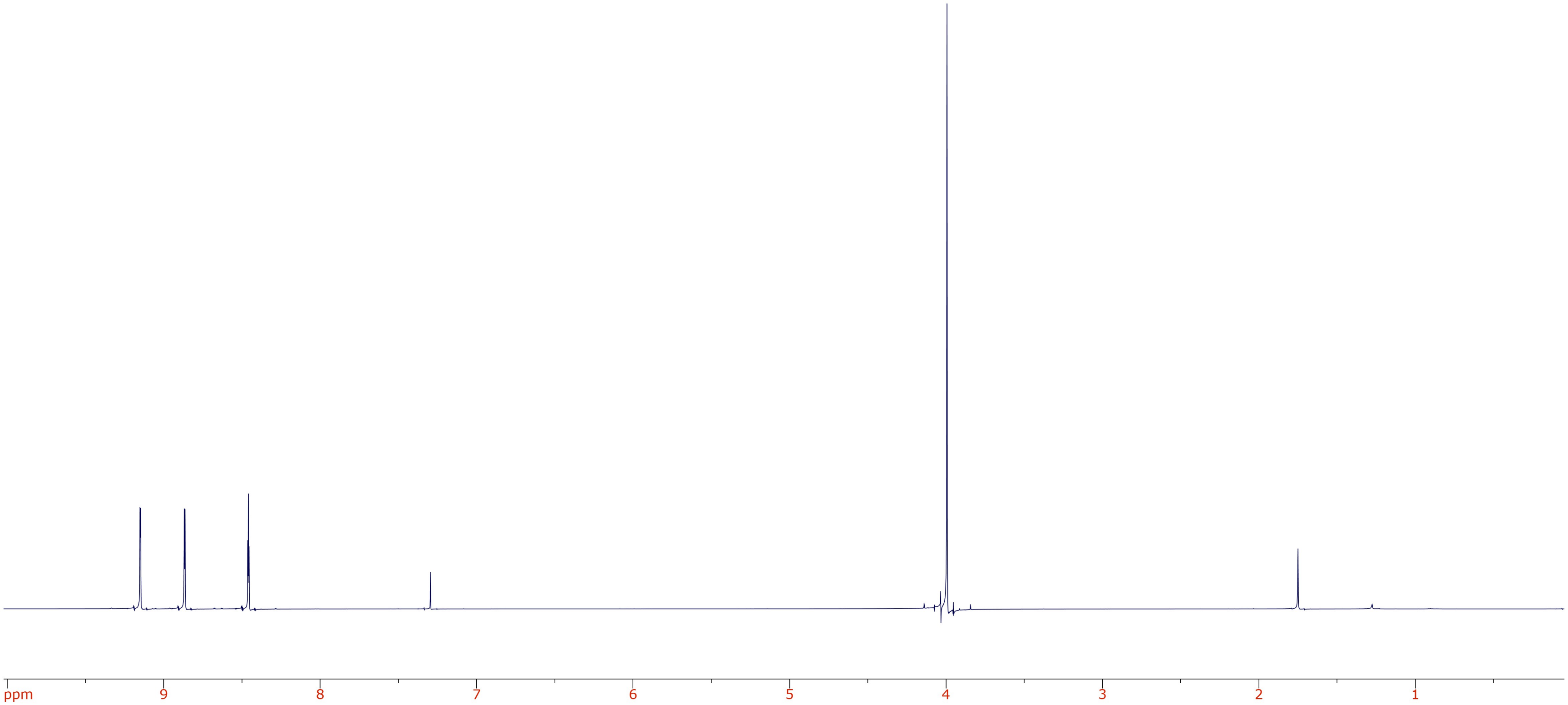
ppm
9
8
7
6
5
4
3
2
1

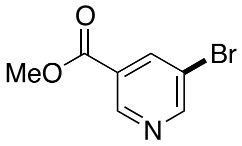

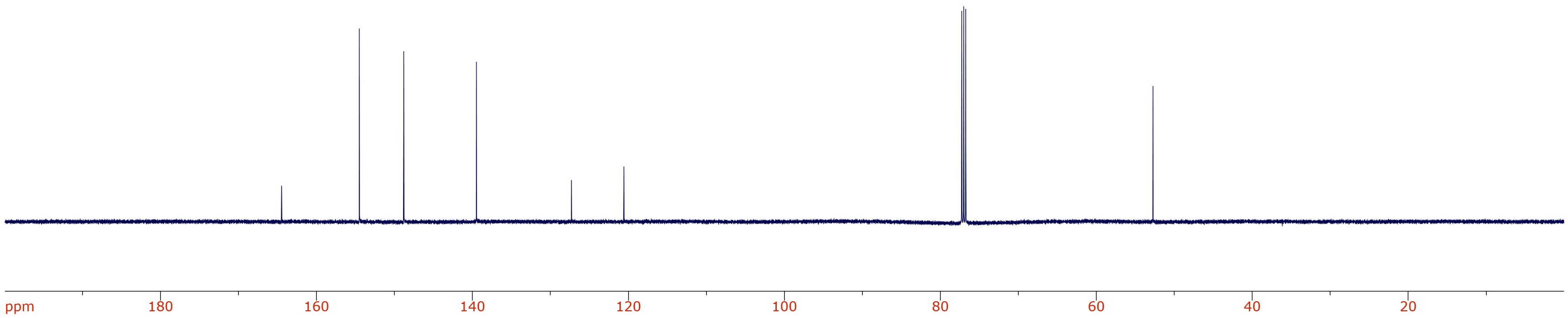

ppm
180
160
140
120
100
80
60
40
20

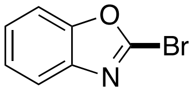
O
N
Br

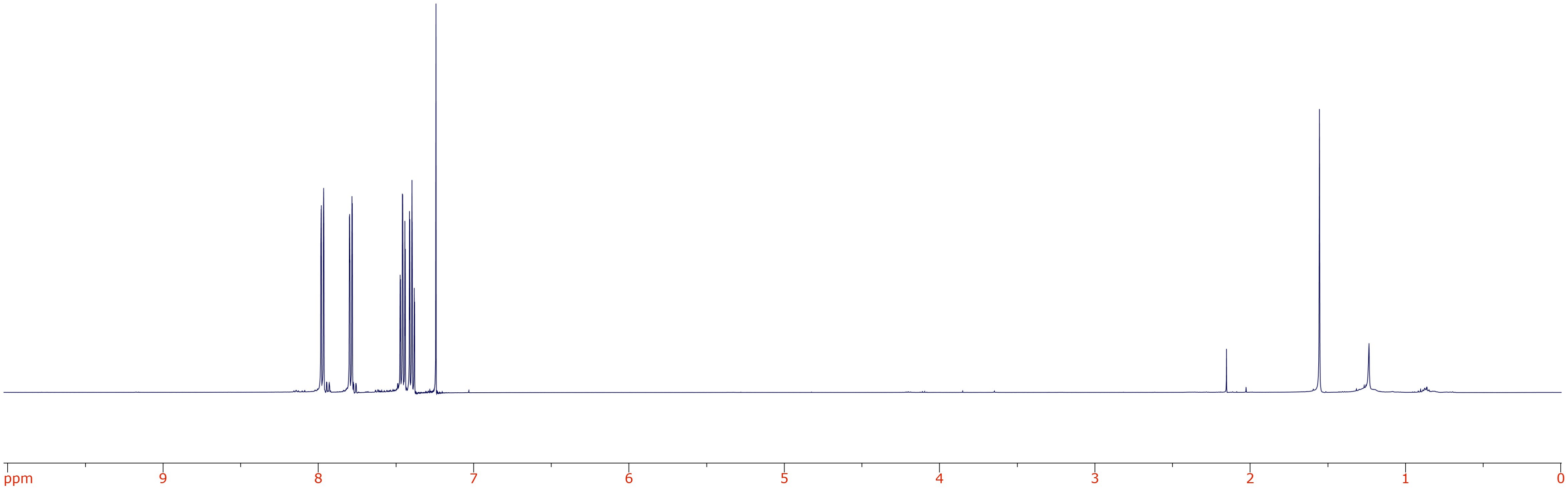
ppm
9
8
7
6
5
4
3
2
1
0

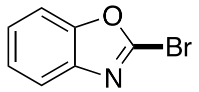

O
Br
N

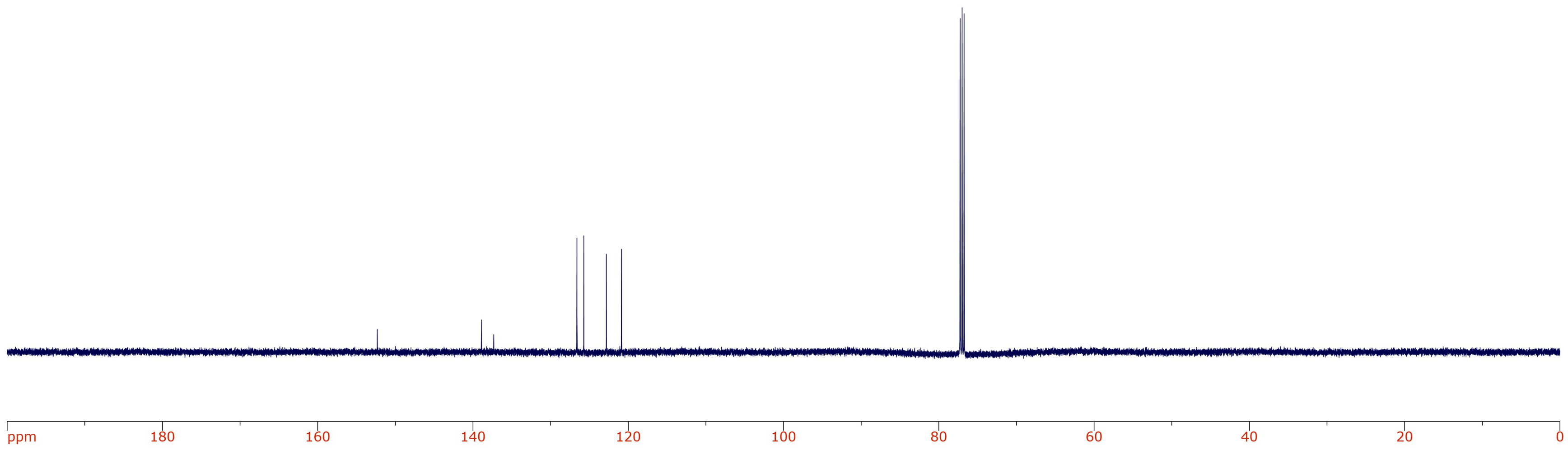

ppm
180
160
140
120
100
80
60
40
20
0

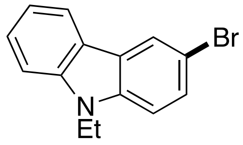
Br
N
Et

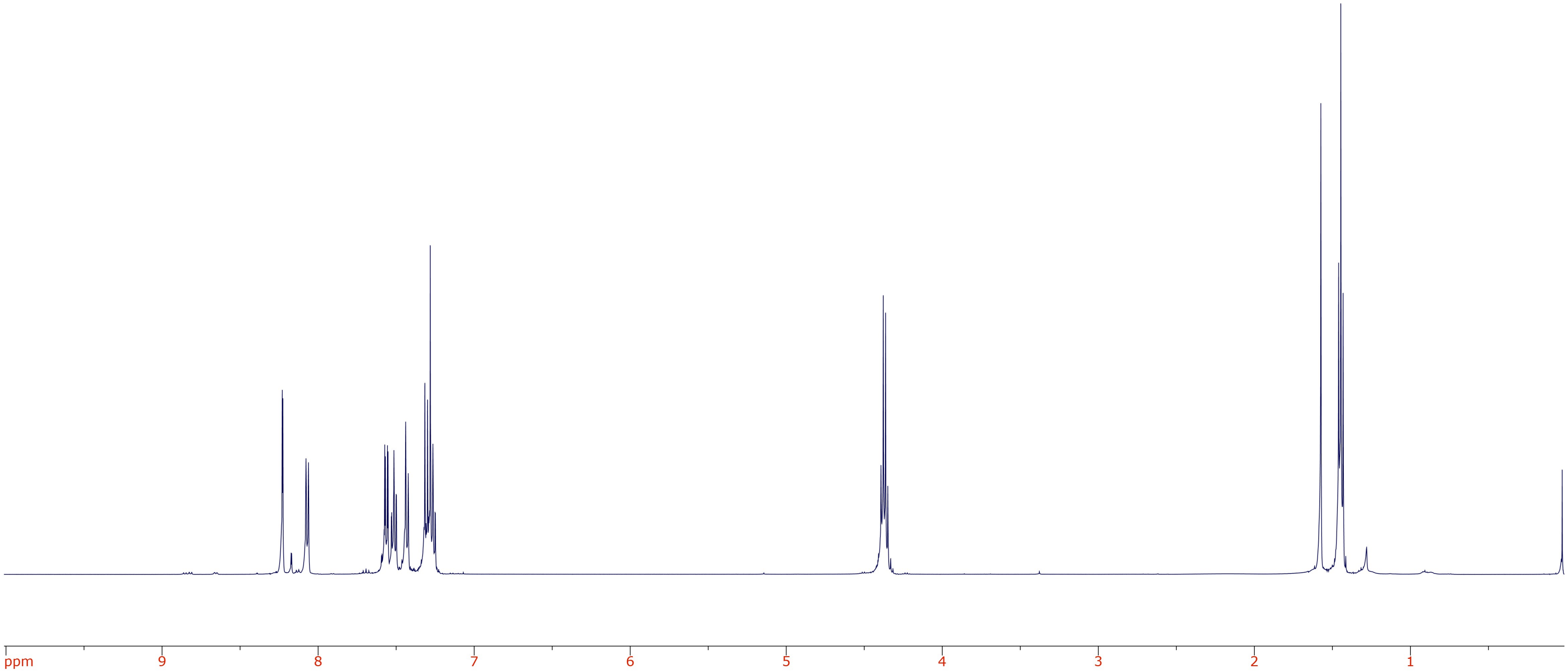
ppm
9
8
7
6
5
4
3
2
1

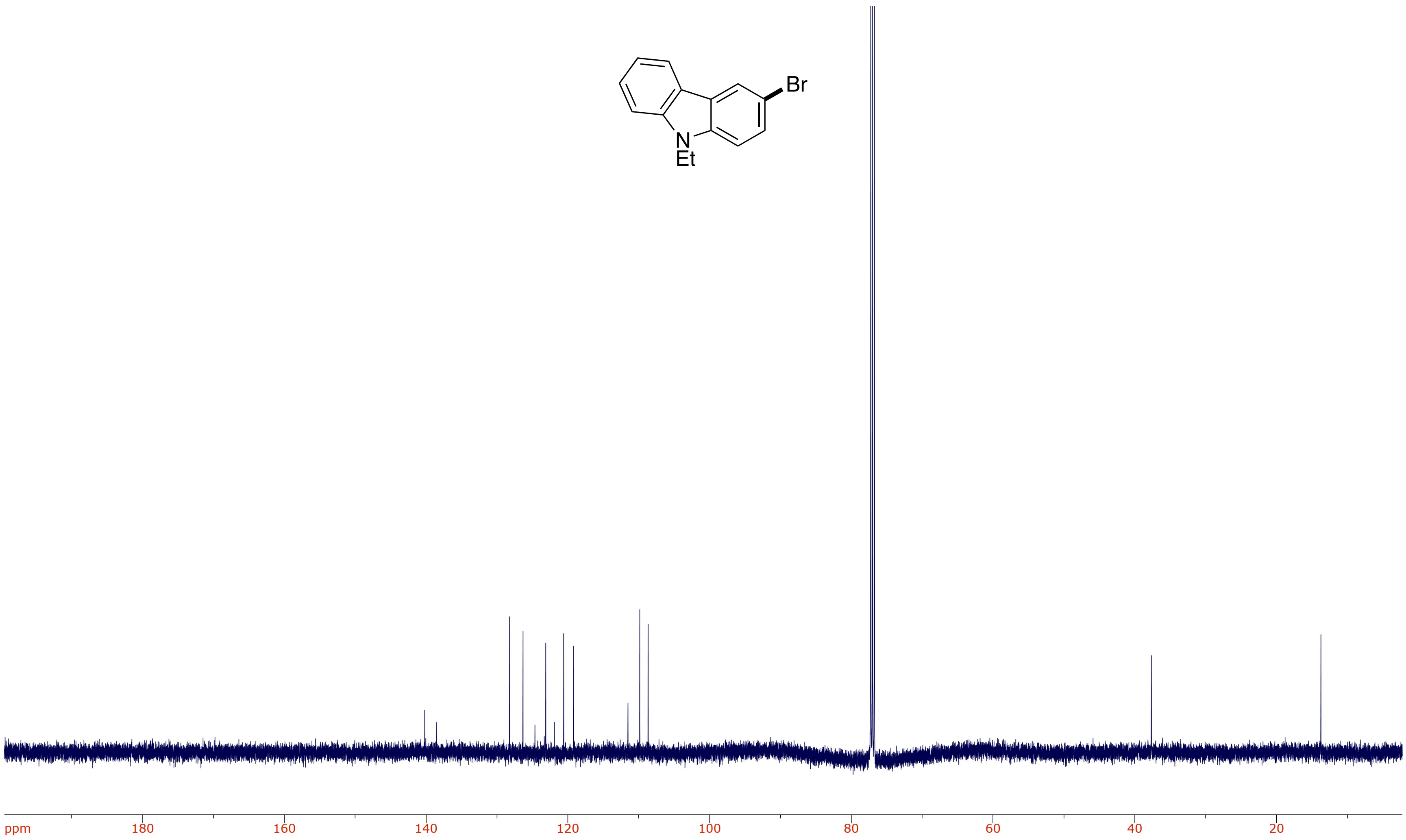
Br
N
Et
ppm
180
160
140
120
100
80
60
40
20

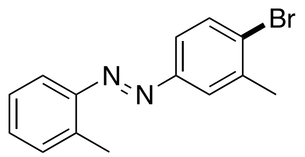
Br
N
N

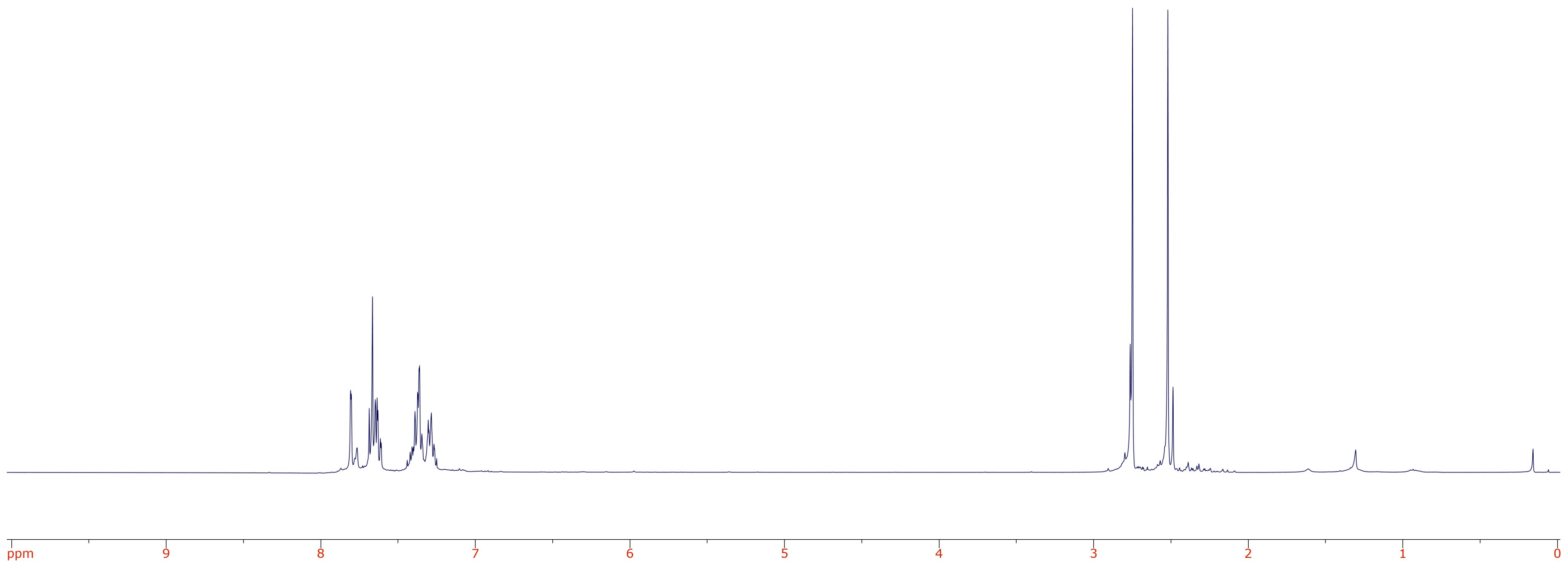
ppm
9
8
7
6
5
4
3
2
1
0

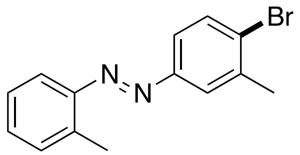
Br
N
N

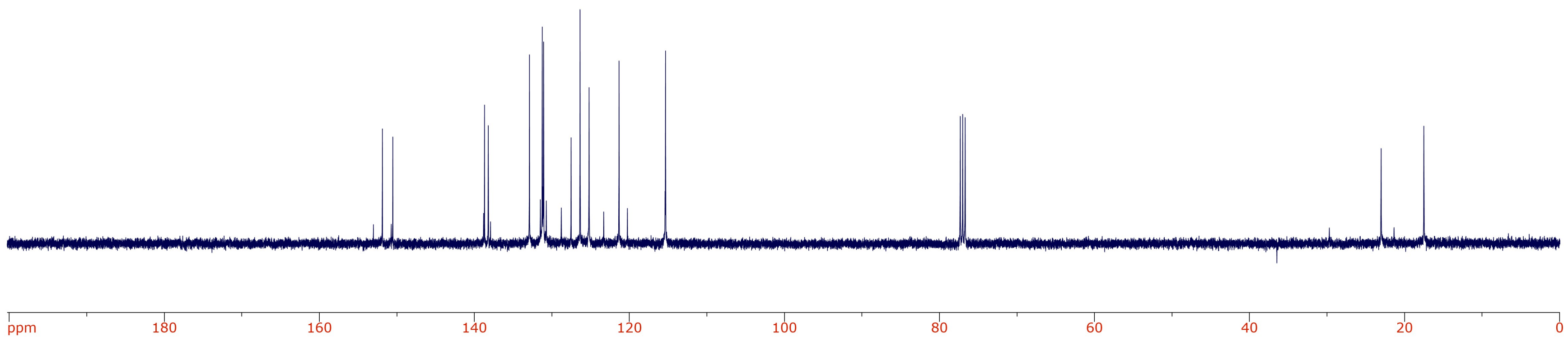
ppm
180
160
140
120
100
80
60
40
20
0

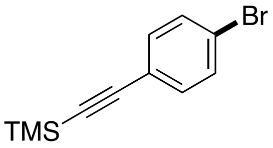
Br
TMS

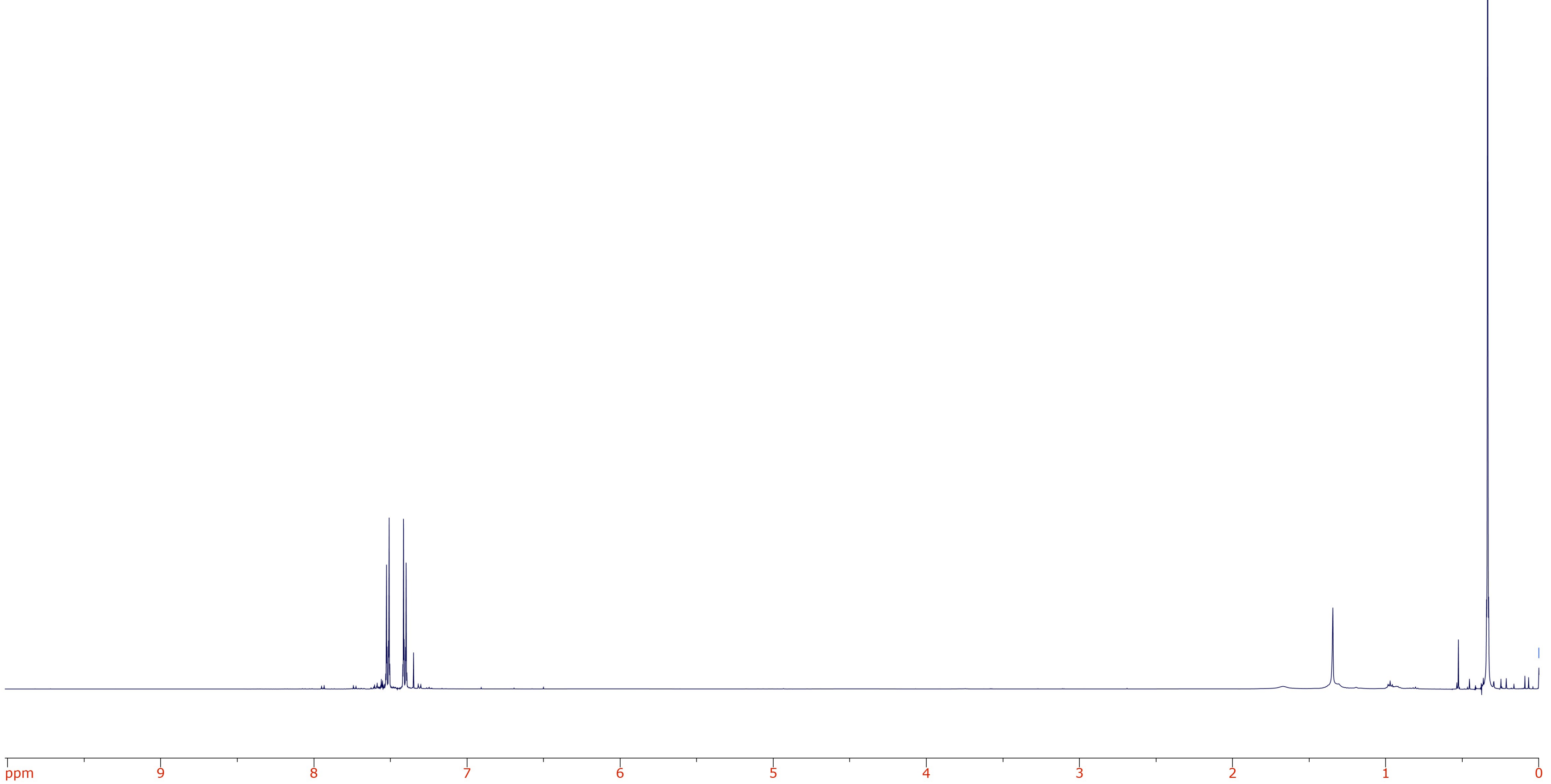
0.000
ppm
9
8
7
6
5
4
3
2
1
0

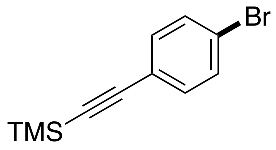
Br
TMS

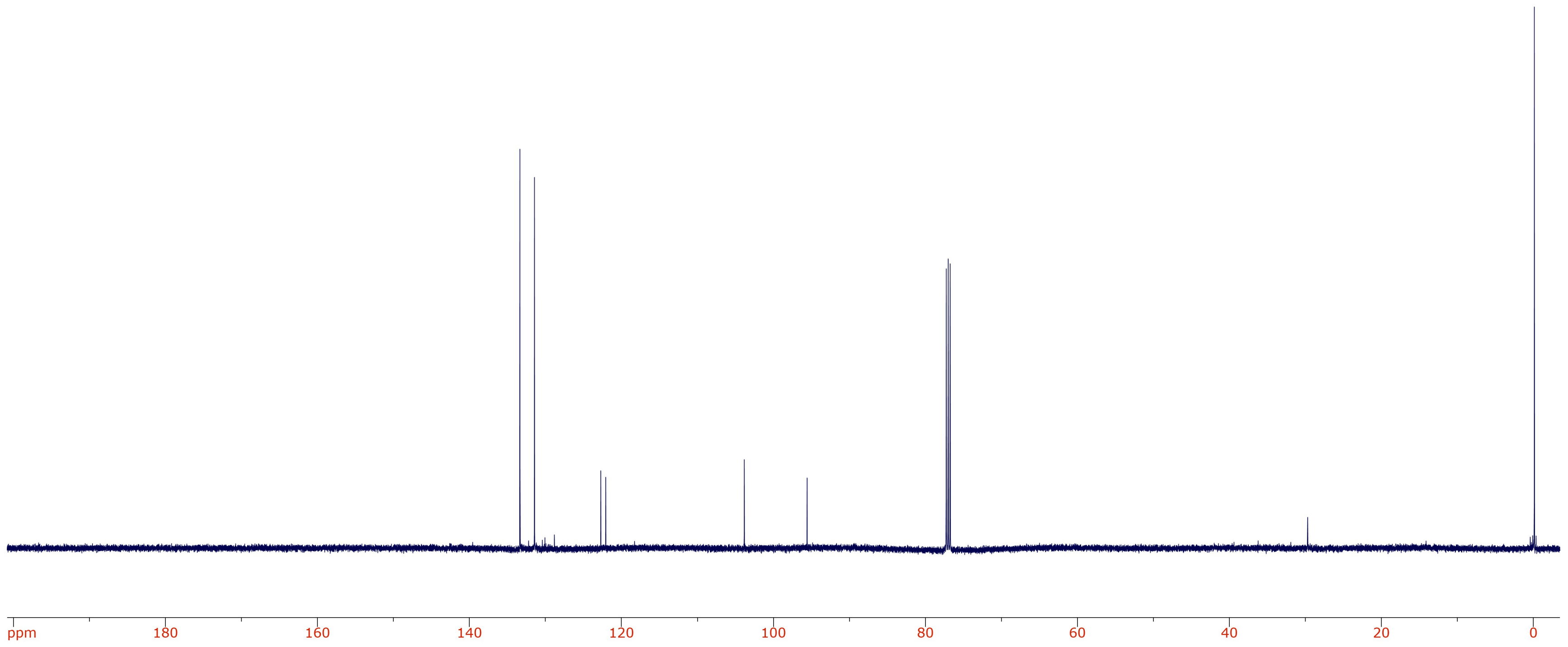
ppm
180
160
140
120
100
80
60
40
20
0

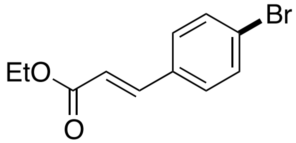

EtO
O
Br

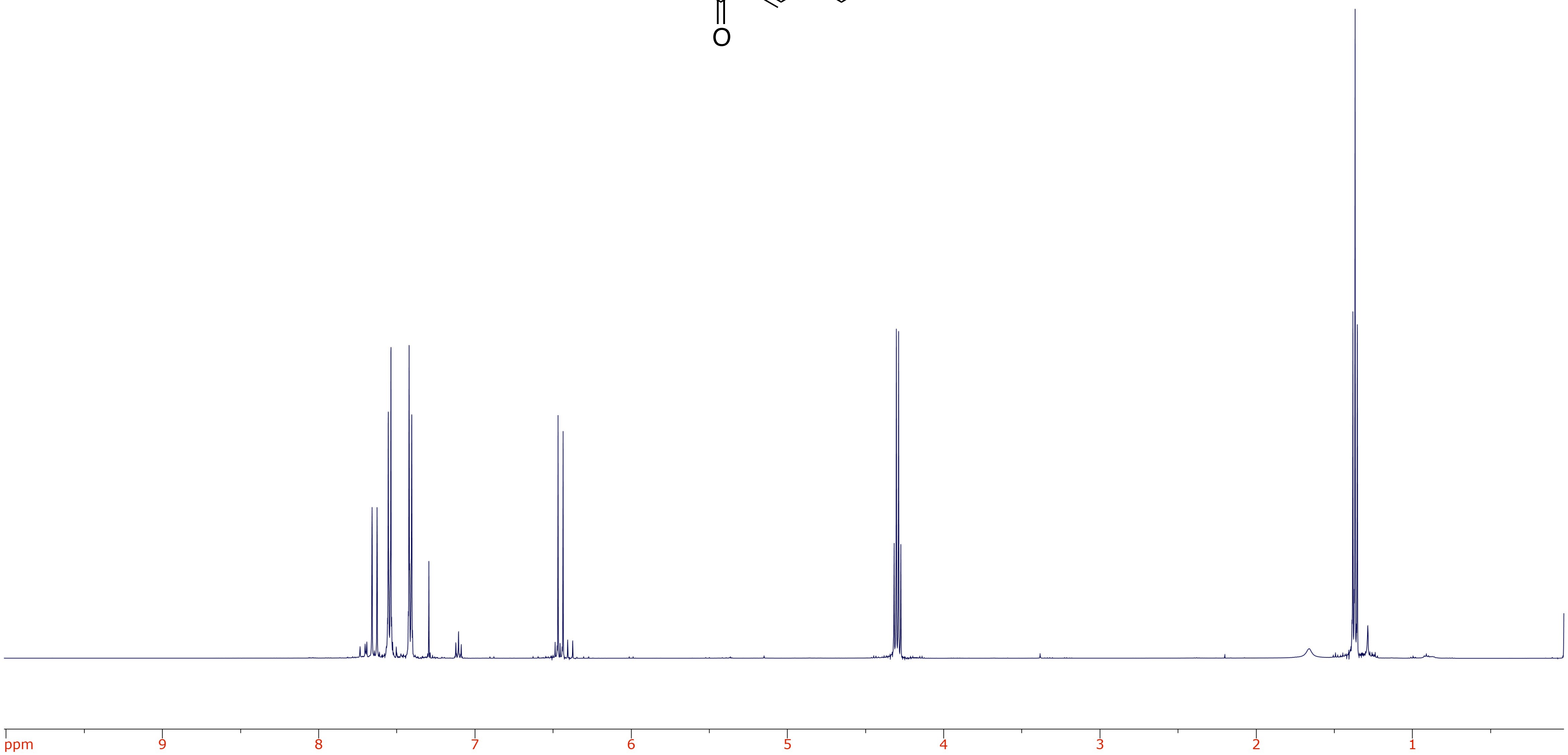

ppm
9
8
7
6
5
4
3
2
1

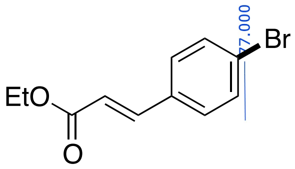
EtO
O
Br
77.000

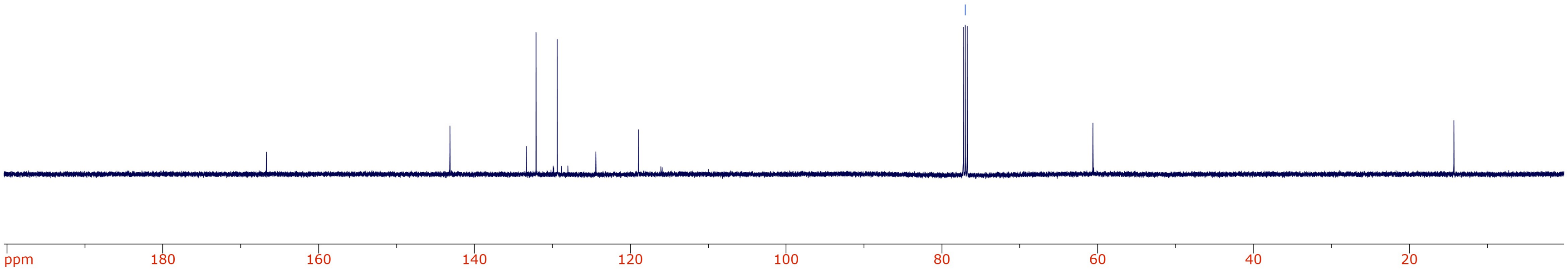
ppm
180
160
140
120
100
80
60
40
20

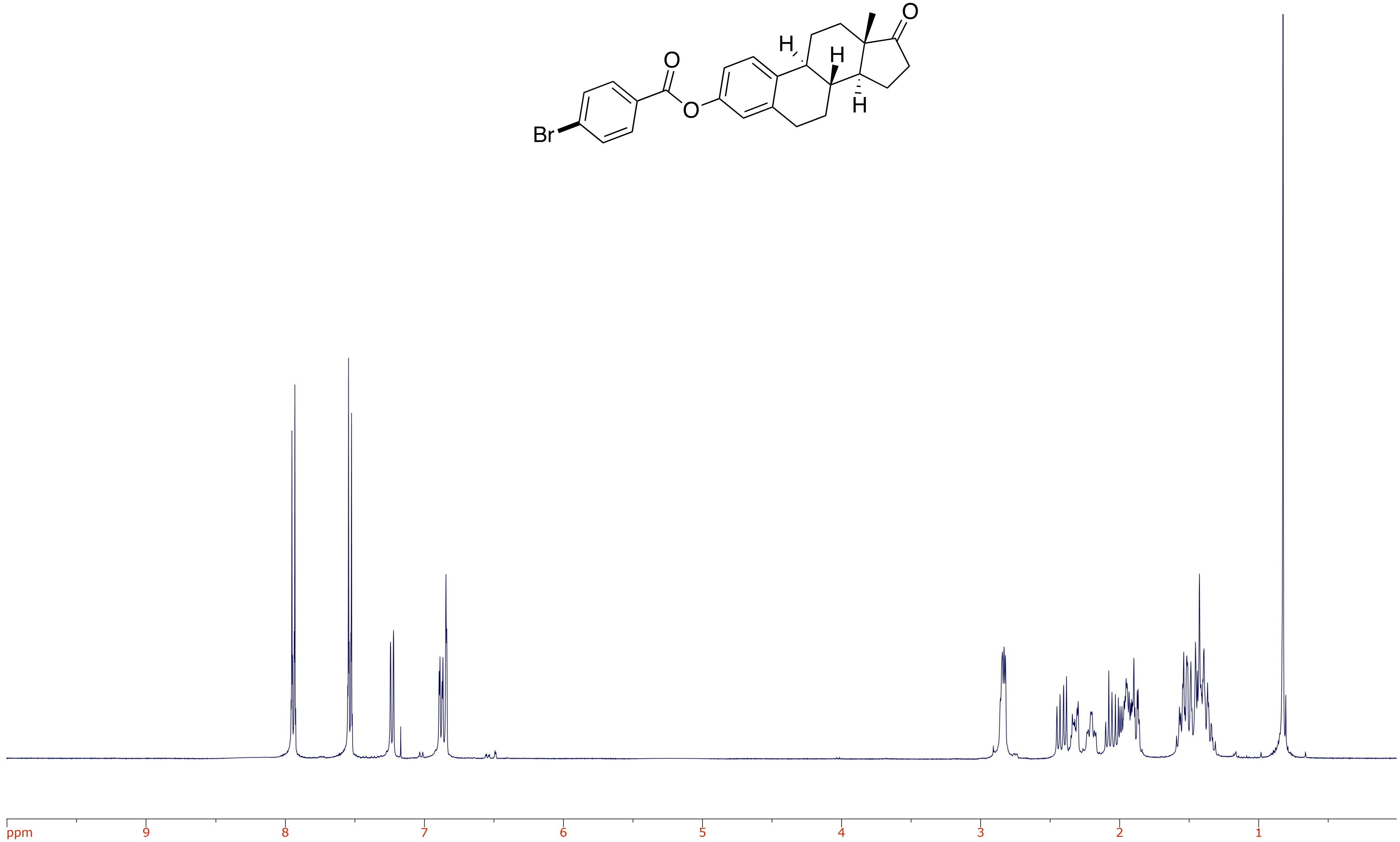

Br
O
O
H
H
H
O
ppm
9
8
7
6
5
4
3
2
1

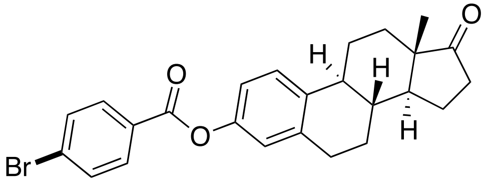
Br
O
O
H
H
H
O

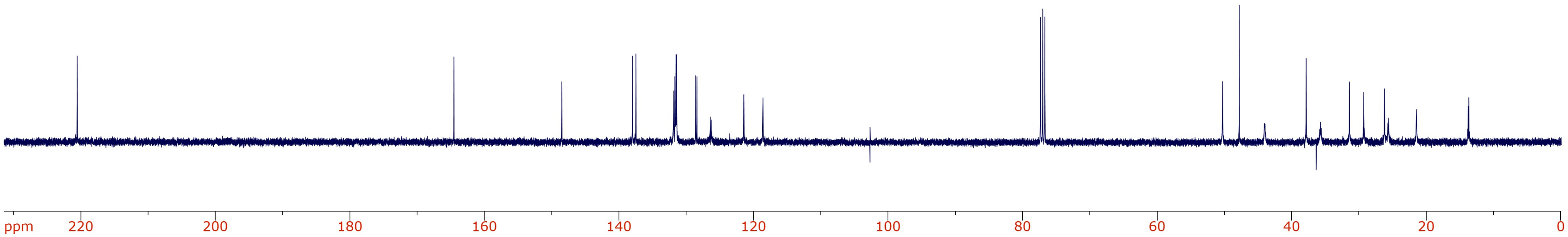
ppm
220
200
180
160
140
120
100
80
60
40
20
0

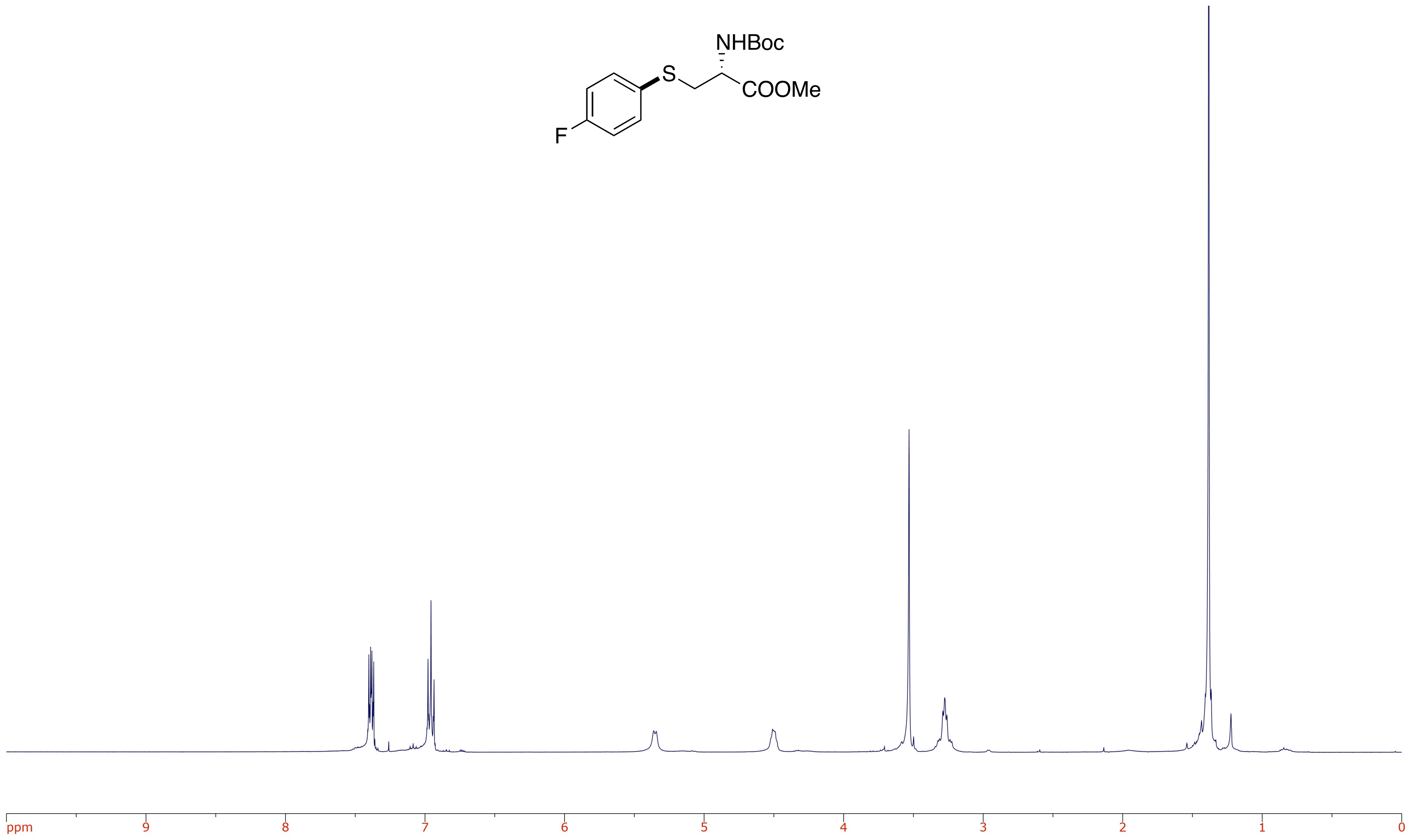
NHBoc
S
COOMe
F
ppm
9
8
7
6
5
4
3
2
1
0

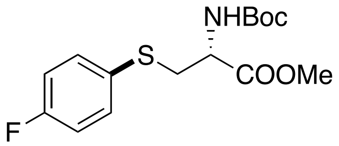
NHBoc
S
COOMe
F

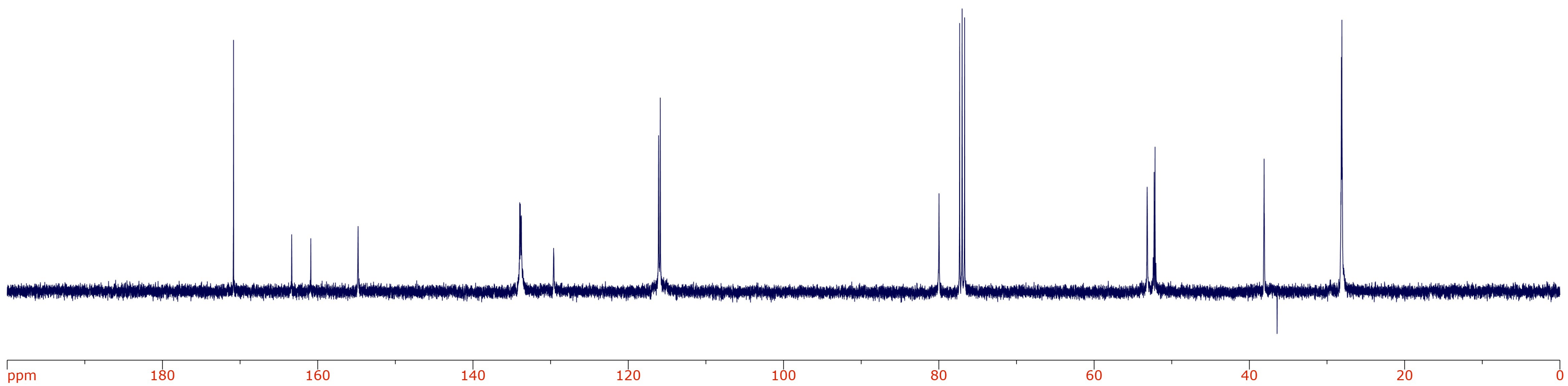
ppm
180
160
140
120
100
80
60
40
20
0

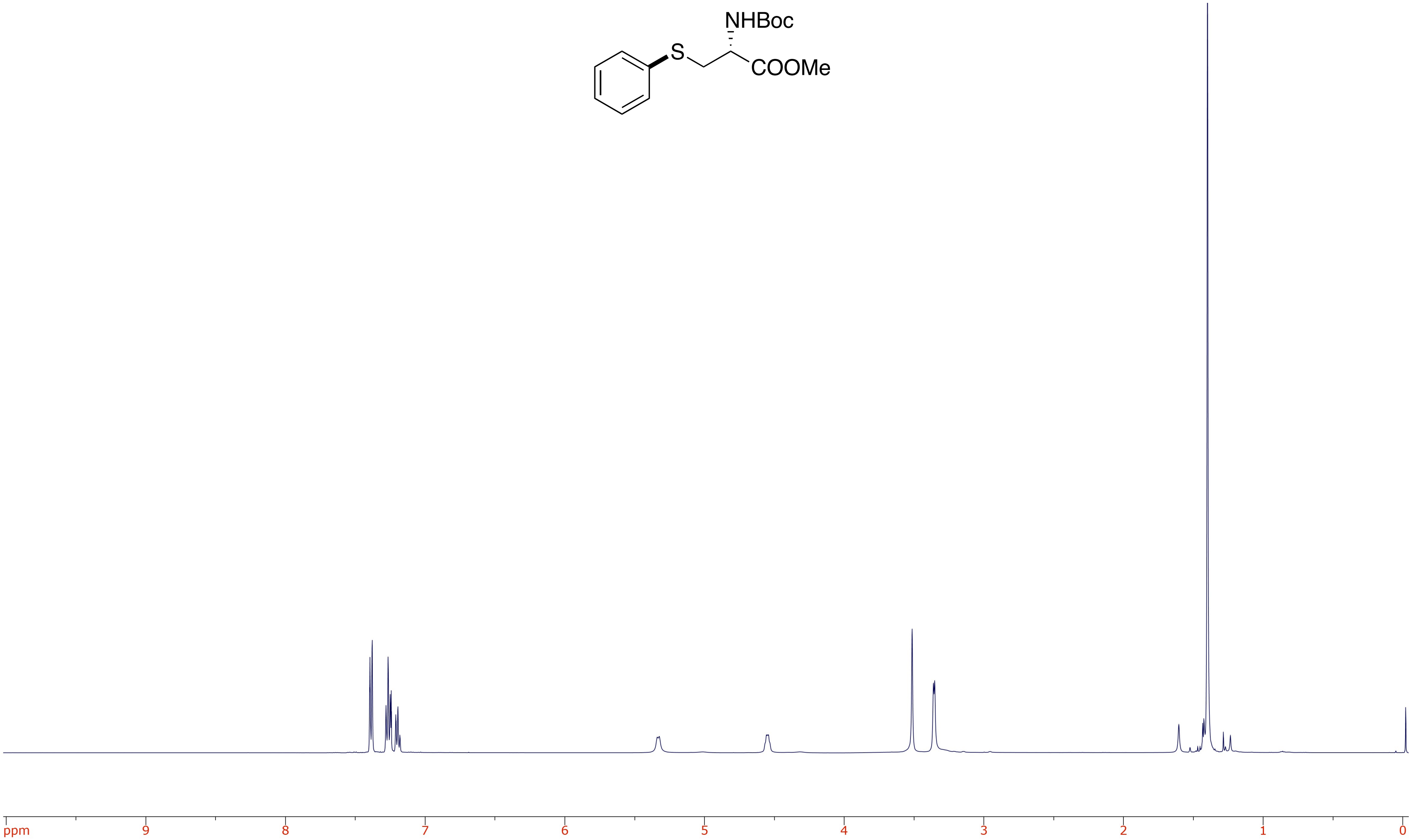
NHBoc
S
COOMe
ppm
9
8
7
6
5
4
3
2
1
0

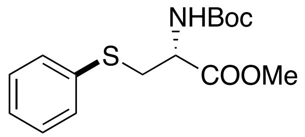
NHBoc
S
COOMe

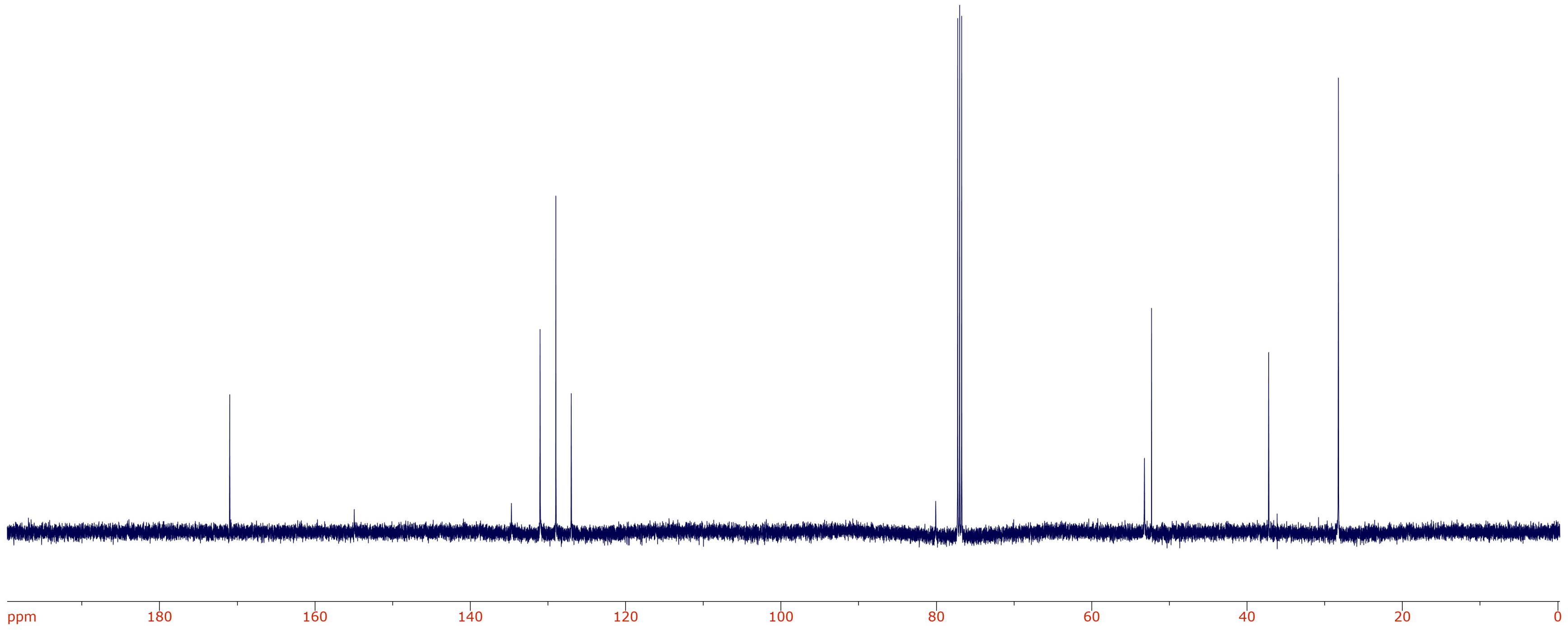
ppm
180
160
140
120
100
80
60
40
20
0

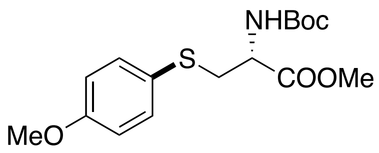
NHBoc
S
COOMe
MeO

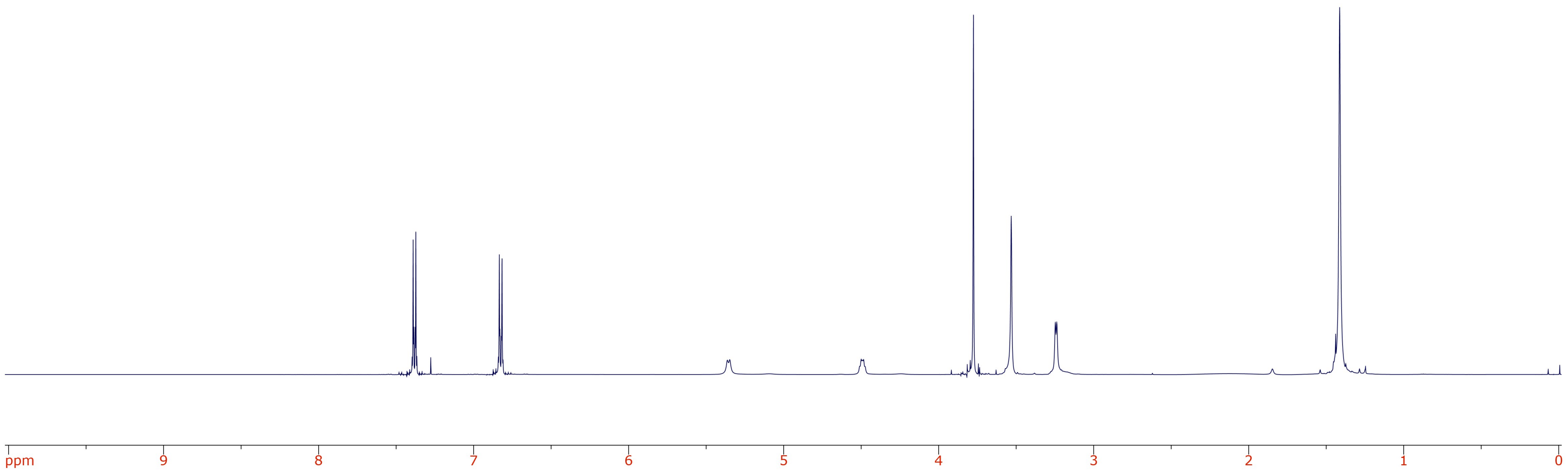
ppm
9
8
7
6
5
4
3
2
1
0

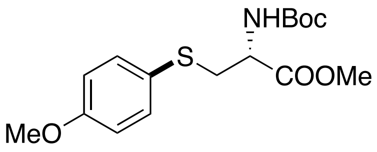
NHBoc
S
COOMe
MeO

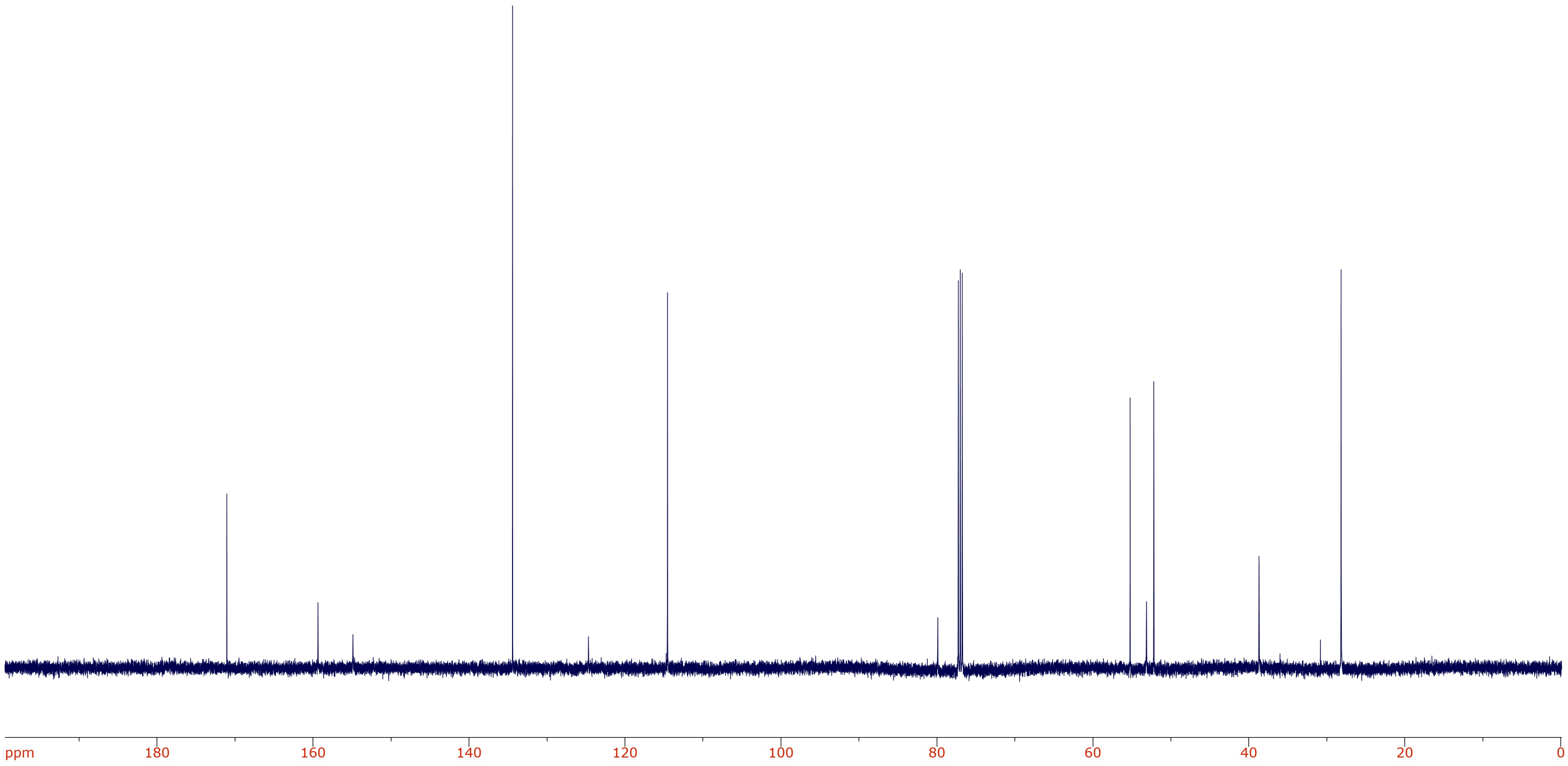
ppm
180
160
140
120
100
80
60
40
20
0

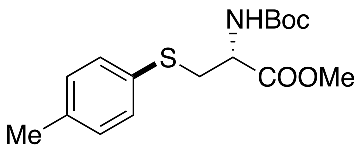
NHBoc
S
COOMe
Me

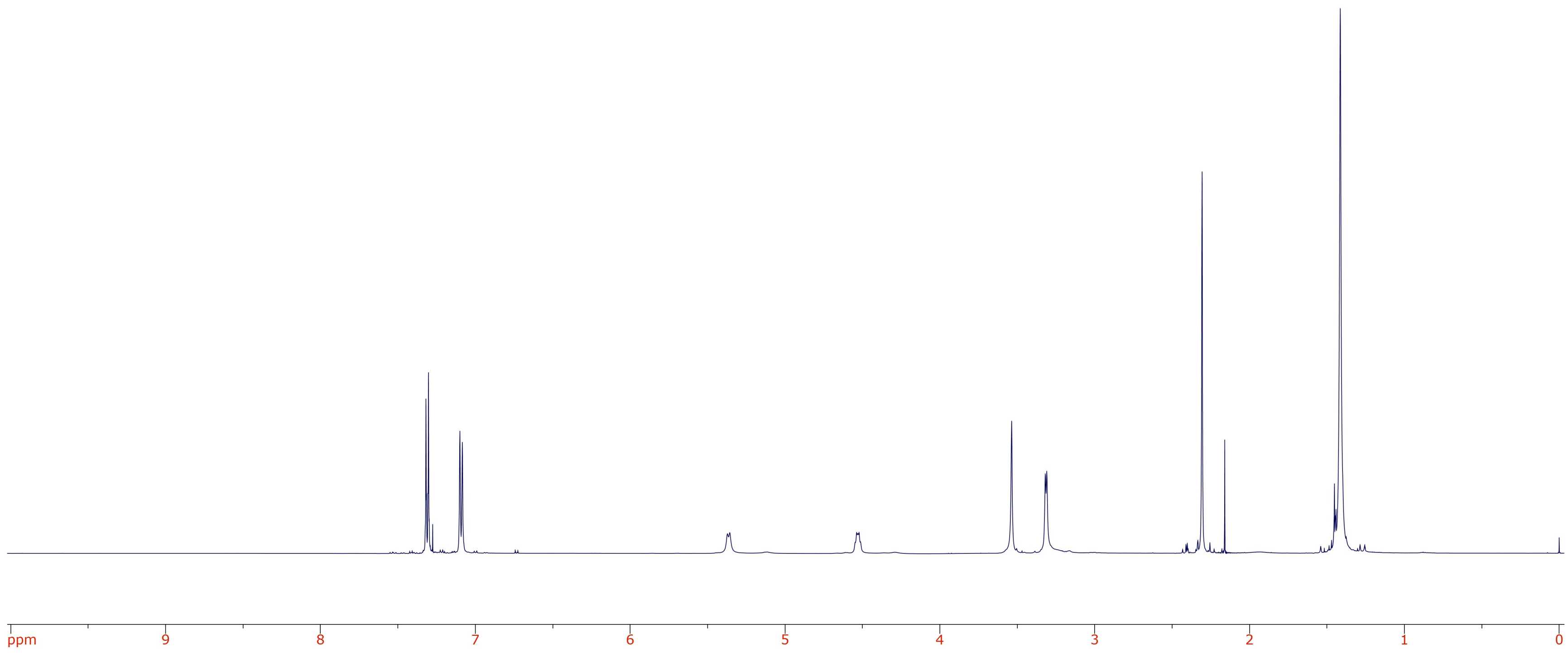
ppm
9
8
7
6
5
4
3
2
1
0

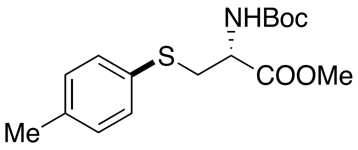
NHBoc
S
COOMe
Me

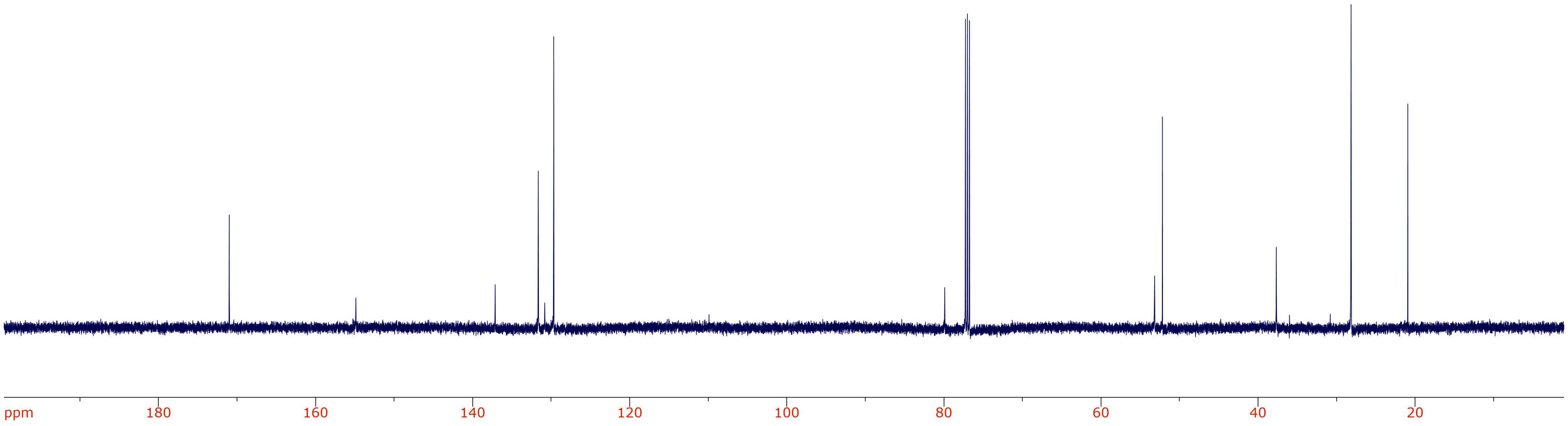
ppm
180
160
140
120
100
80
60
40
20

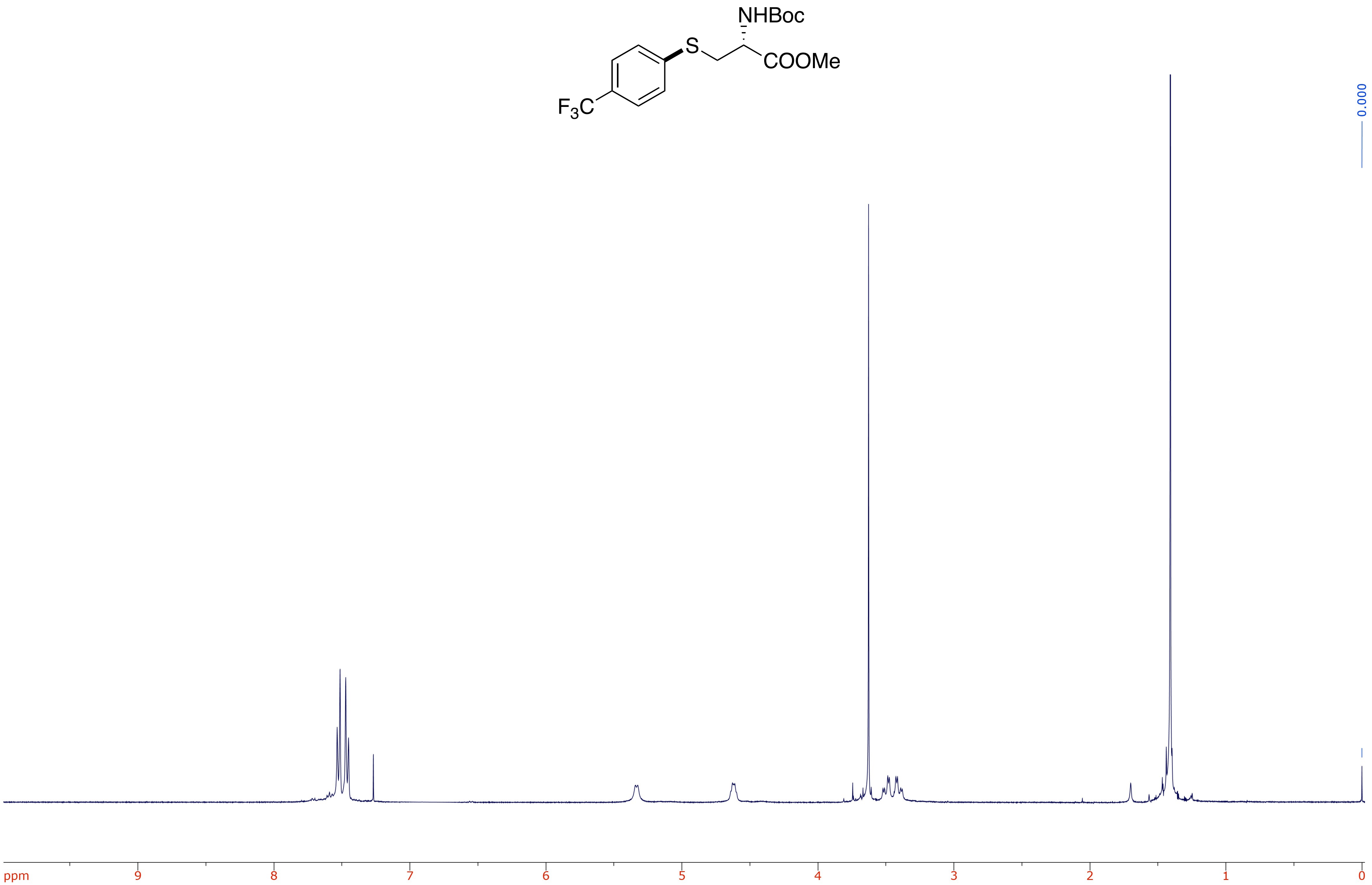
NHBoc
S
COOMe
F3C
0.000
ppm
9
8
7
6
5
4
3
2
1
0

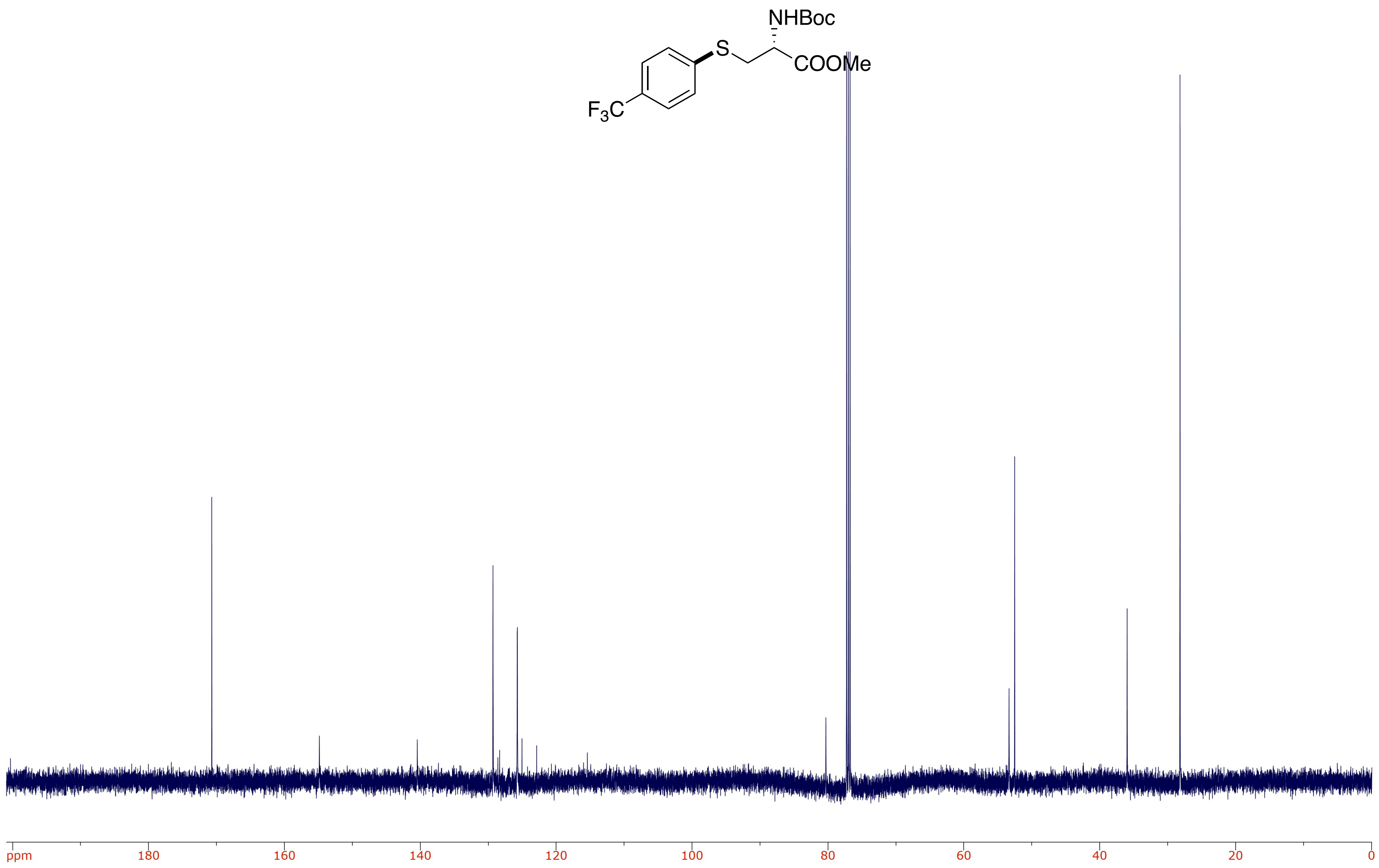
NHBoc
S
COOMe
F3C
ppm
180
160
140
120
100
80
60
40
20
0

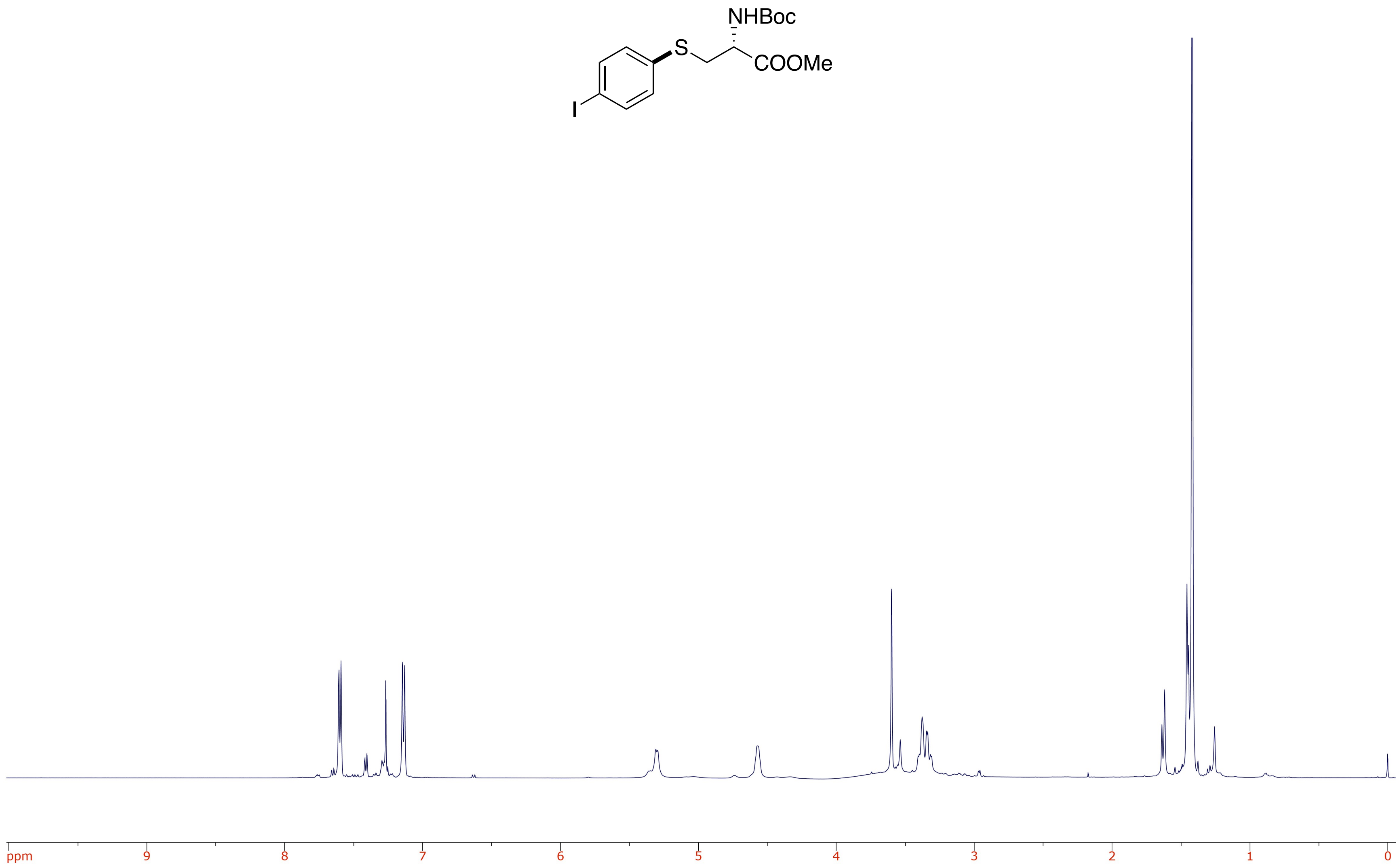
NHBoc
S
COOMe
I
ppm
9
8
7
6
5
4
3
2
1
0

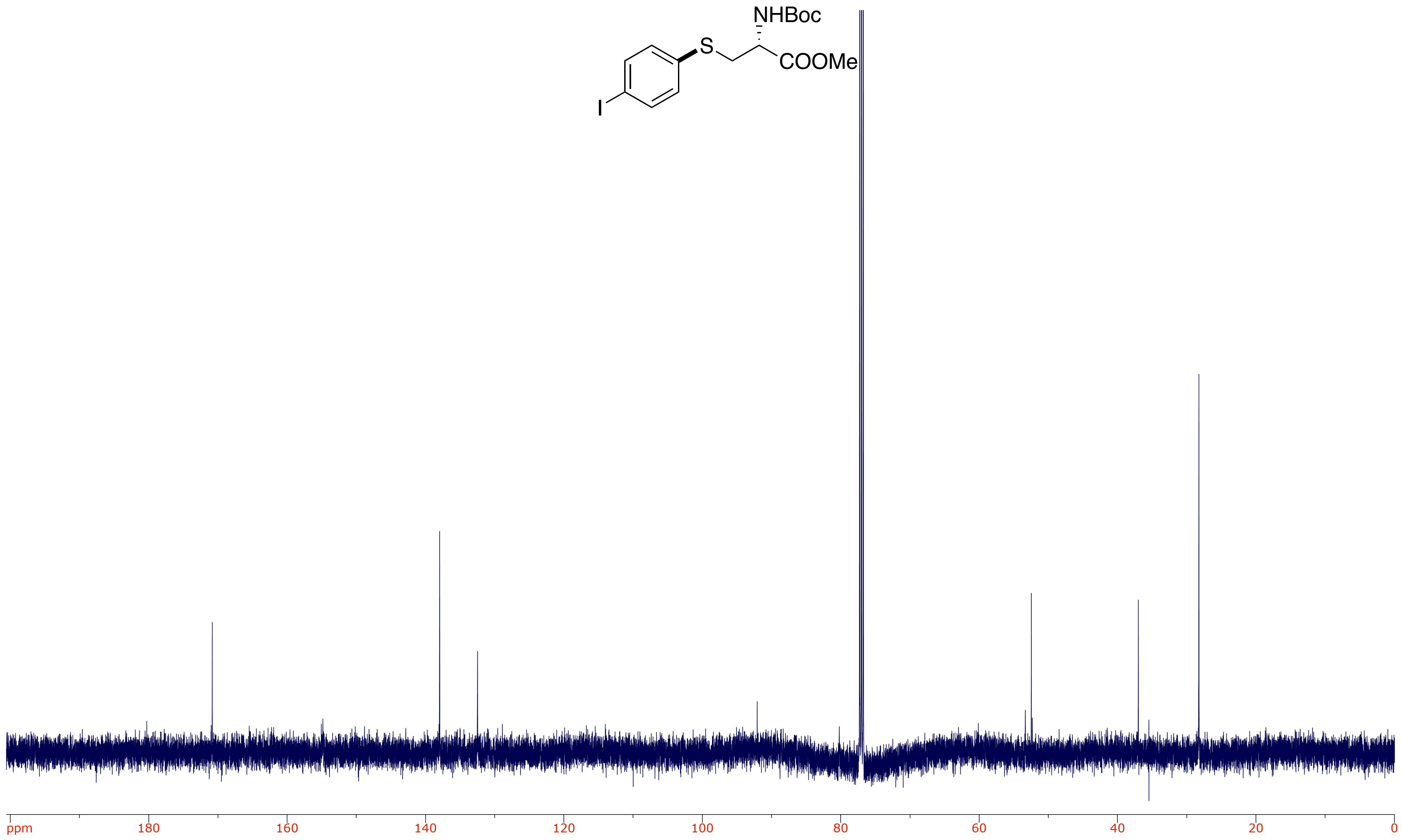
NHBoc
S
COOMe
I
ppm
180
160
140
120
100
80
60
40
20
0

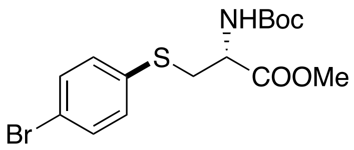
NHBoc
S
COOMe
Br

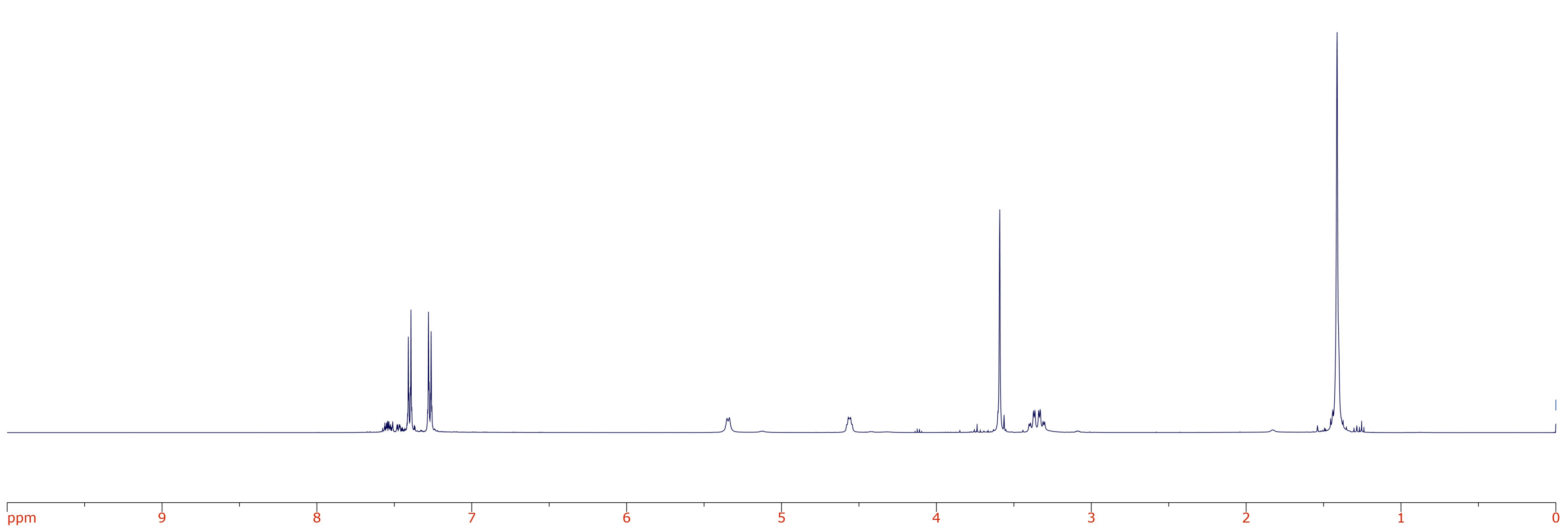
0.000
ppm
9
8
7
6
5
4
3
2
1
0

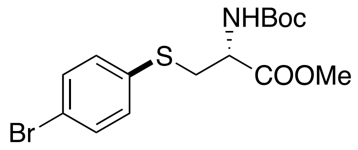
NHBoc
S
COOMe
Br

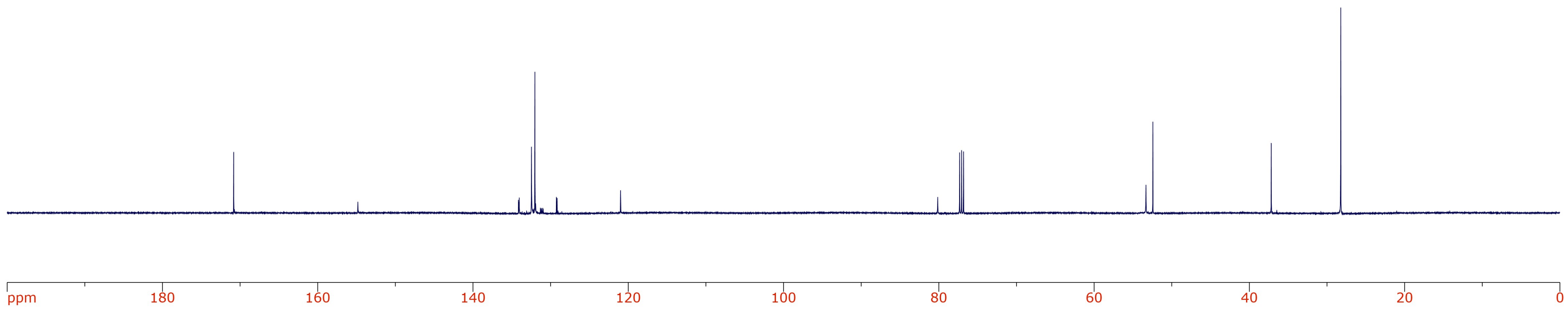
ppm
180
160
140
120
100
80
60
40
20
0

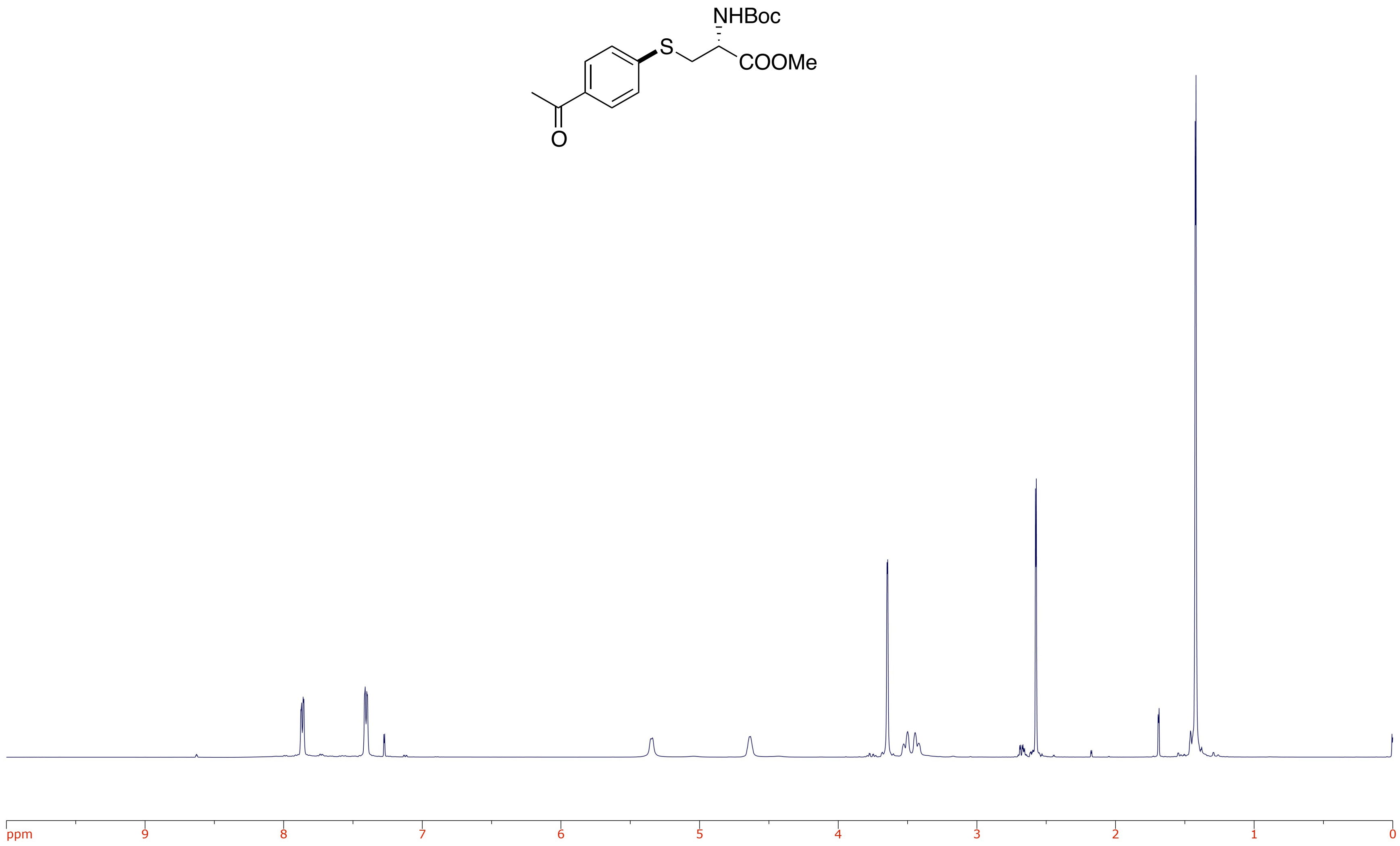
NHBoc
S
COOMe
O
ppm
9
8
7
6
5
4
3
2
1
0

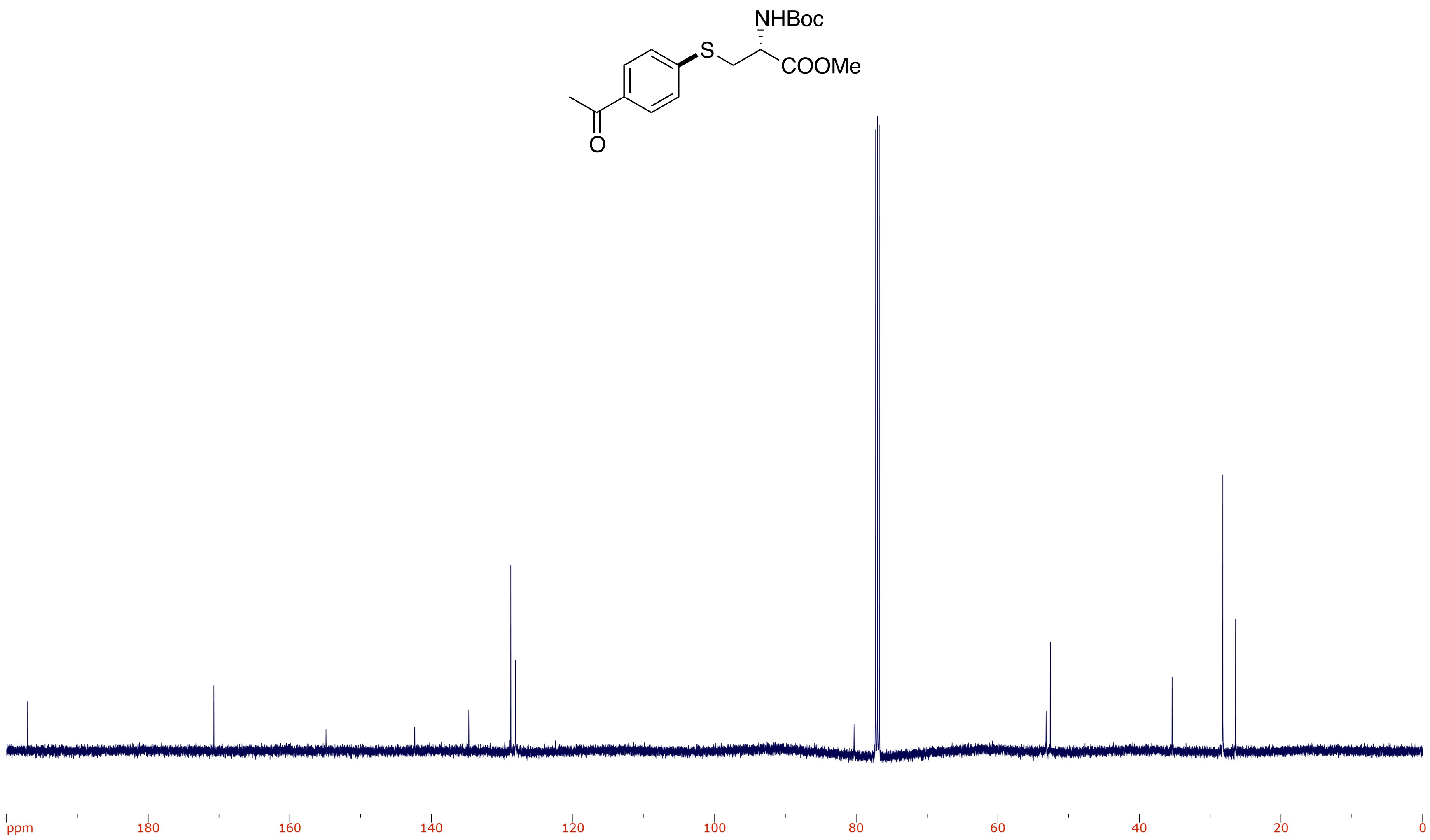
NHBoc
S
COOMe
O
ppm
180
160
140
120
100
80
60
40
20
0

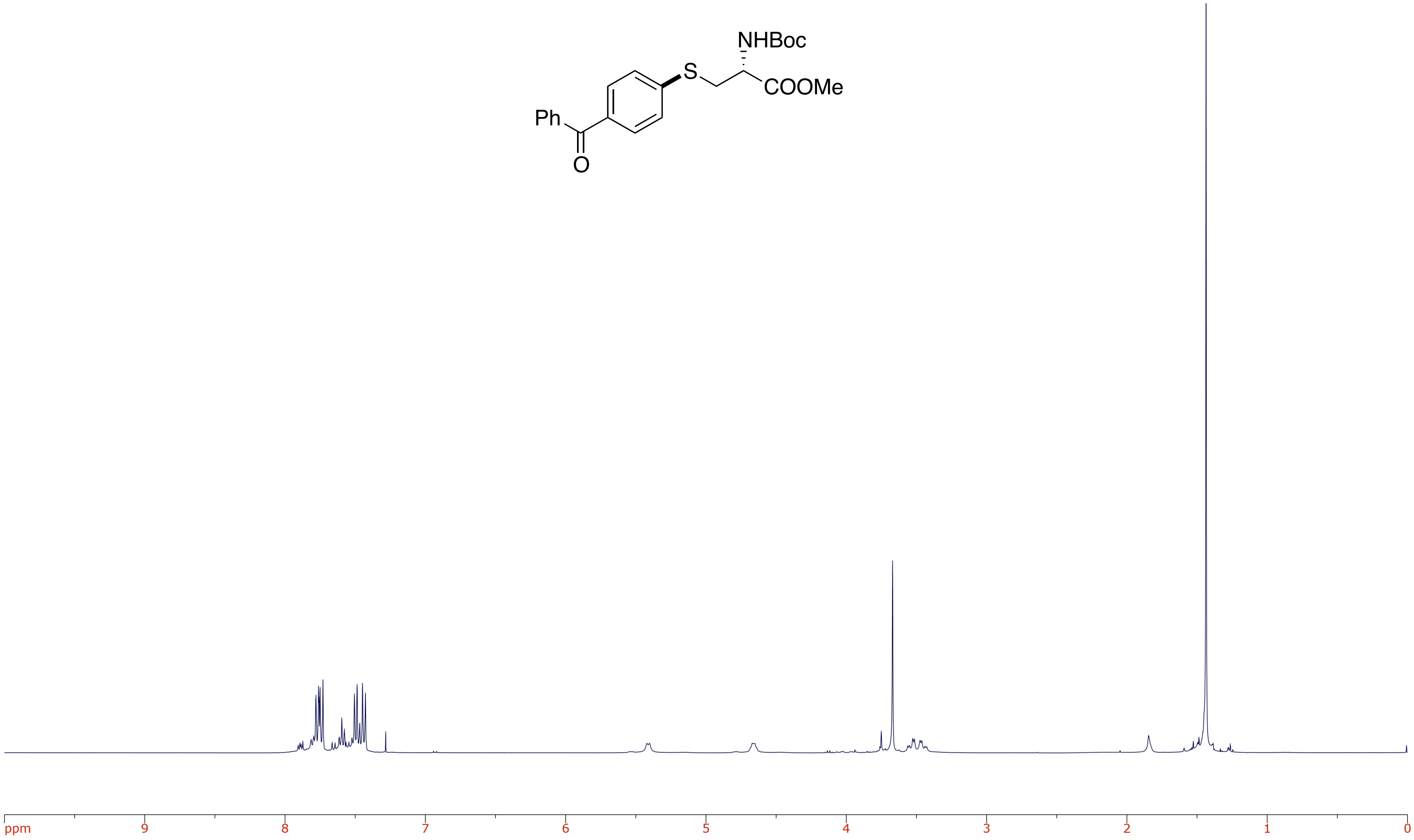
NHBoc
S
COOMe
Ph
O
ppm
9
8
7
6
5
4
3
2
1
0

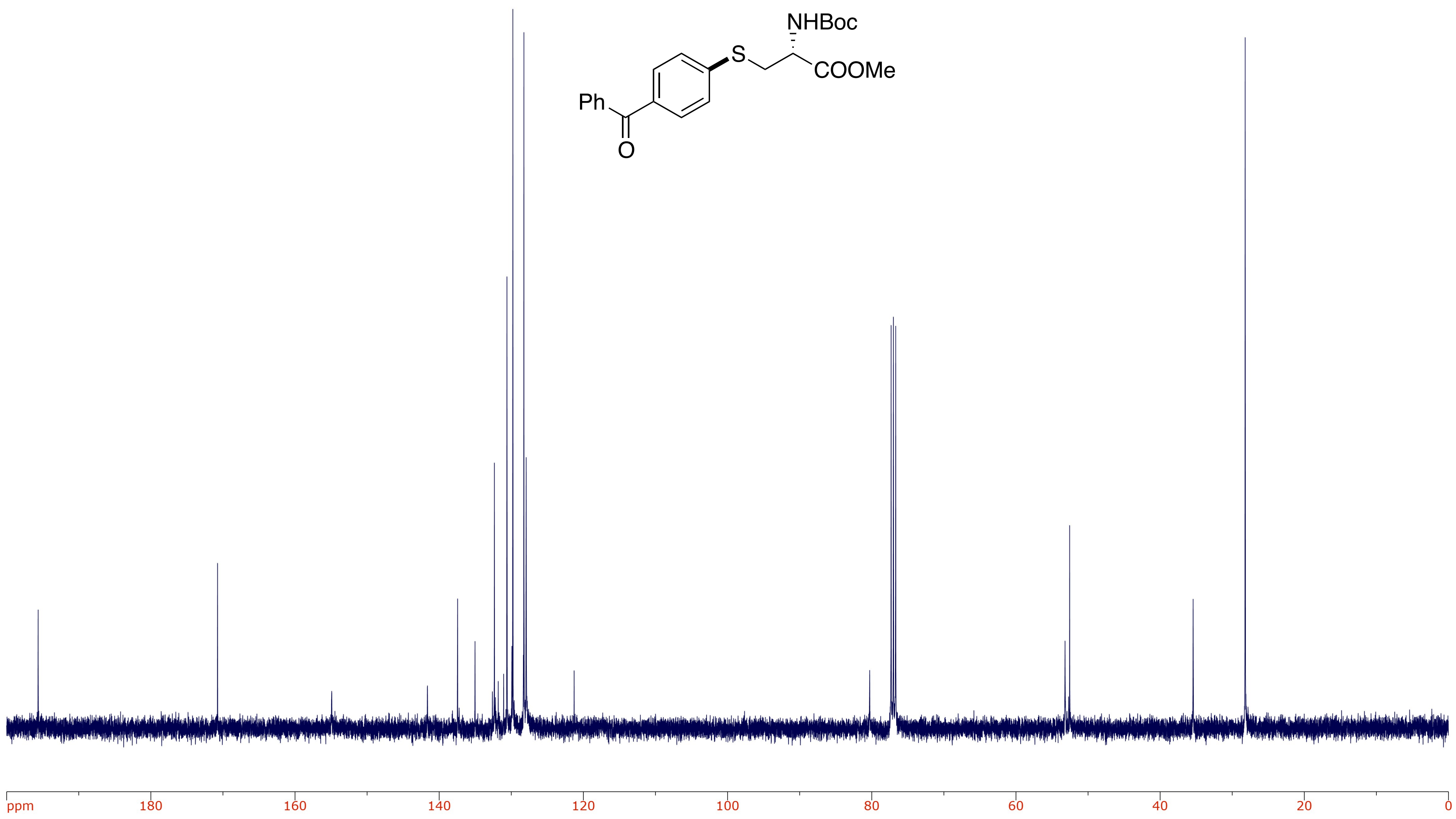

NHBoc
S
COOMe
Ph
O
ppm
180
160
140
120
100
80
60
40
20
0

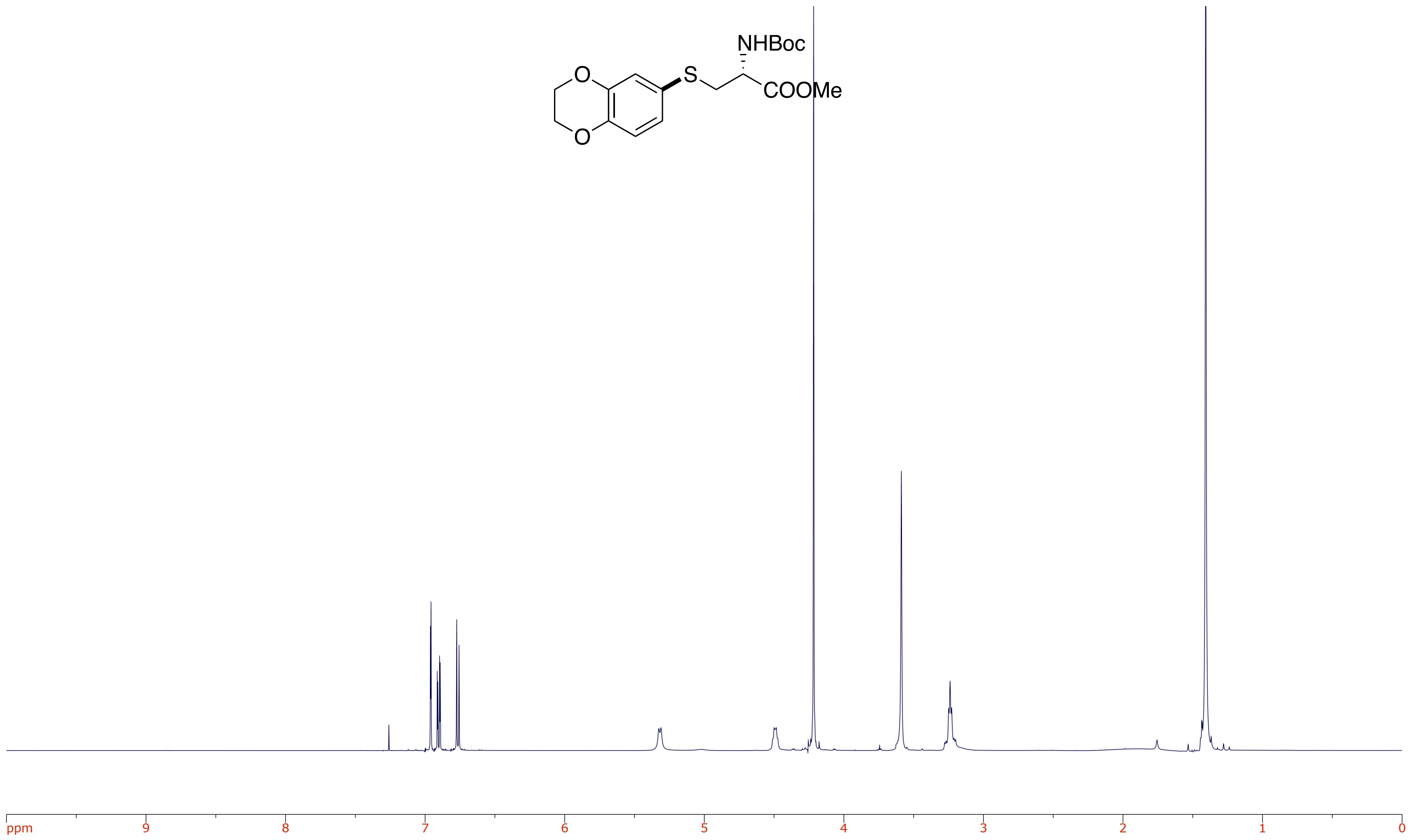

NHBoc
O
O
S
COOMe
ppm
9
8
7
6
5
4
3
2
1
0

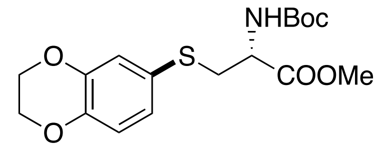
NHBoc
S
COOMe
O
O

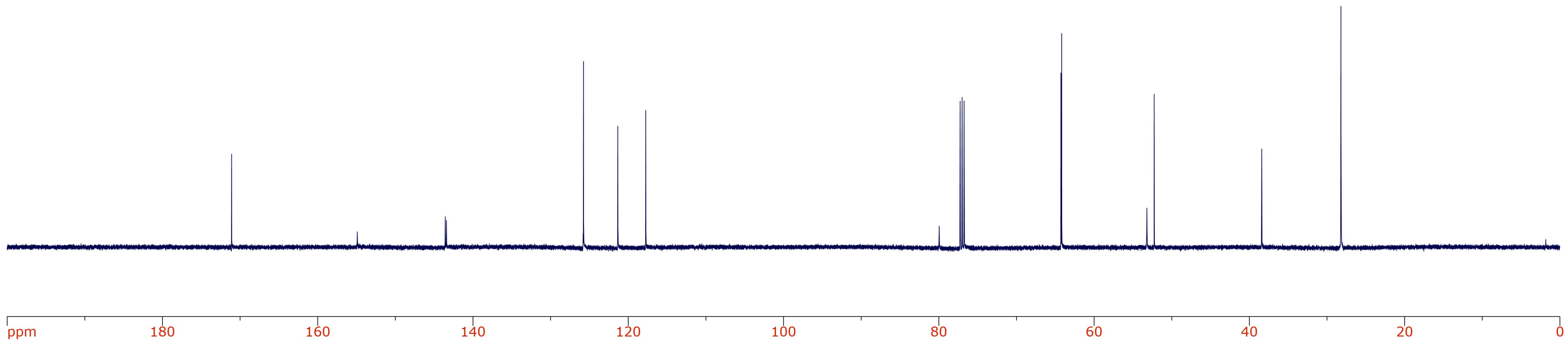
ppm
180
160
140
120
100
80
60
40
20
0

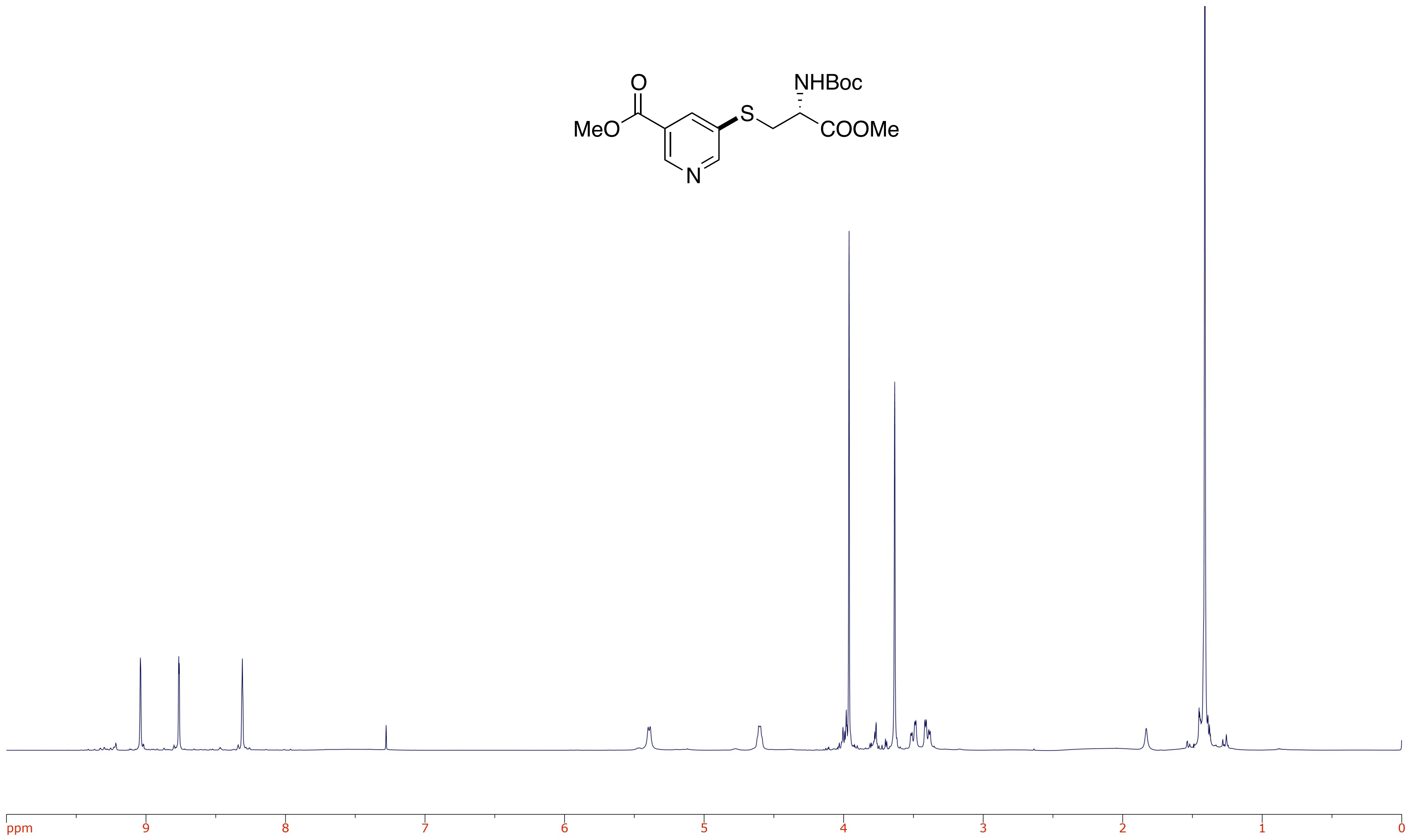
O
MeO
S
NHBoc
COOMe
N
ppm
9
8
7
6
5
4
3
2
1
0

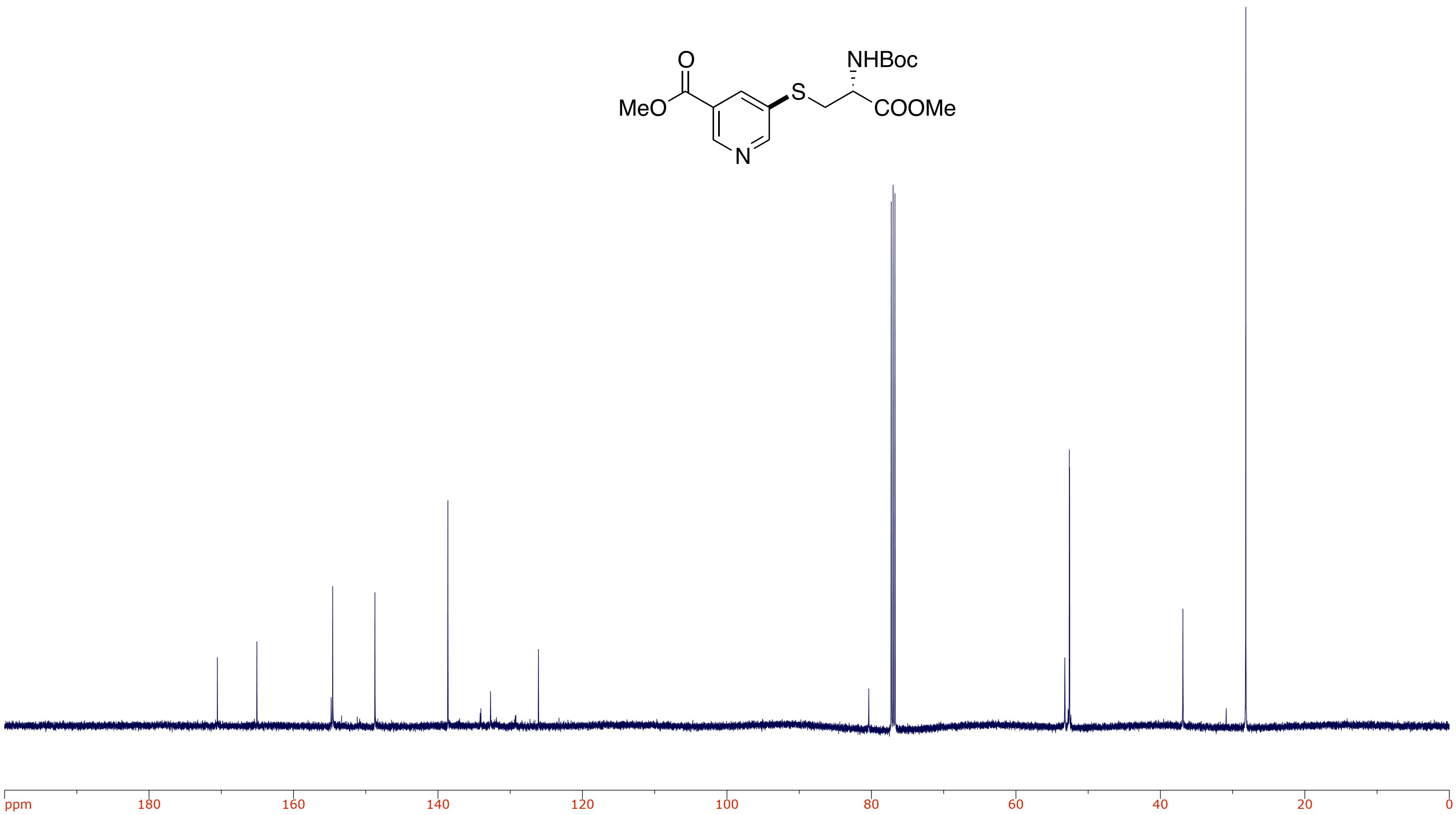
O
MeO
S
NHBoc
COOMe
N
ppm
180
160
140
120
100
80
60
40
20
0

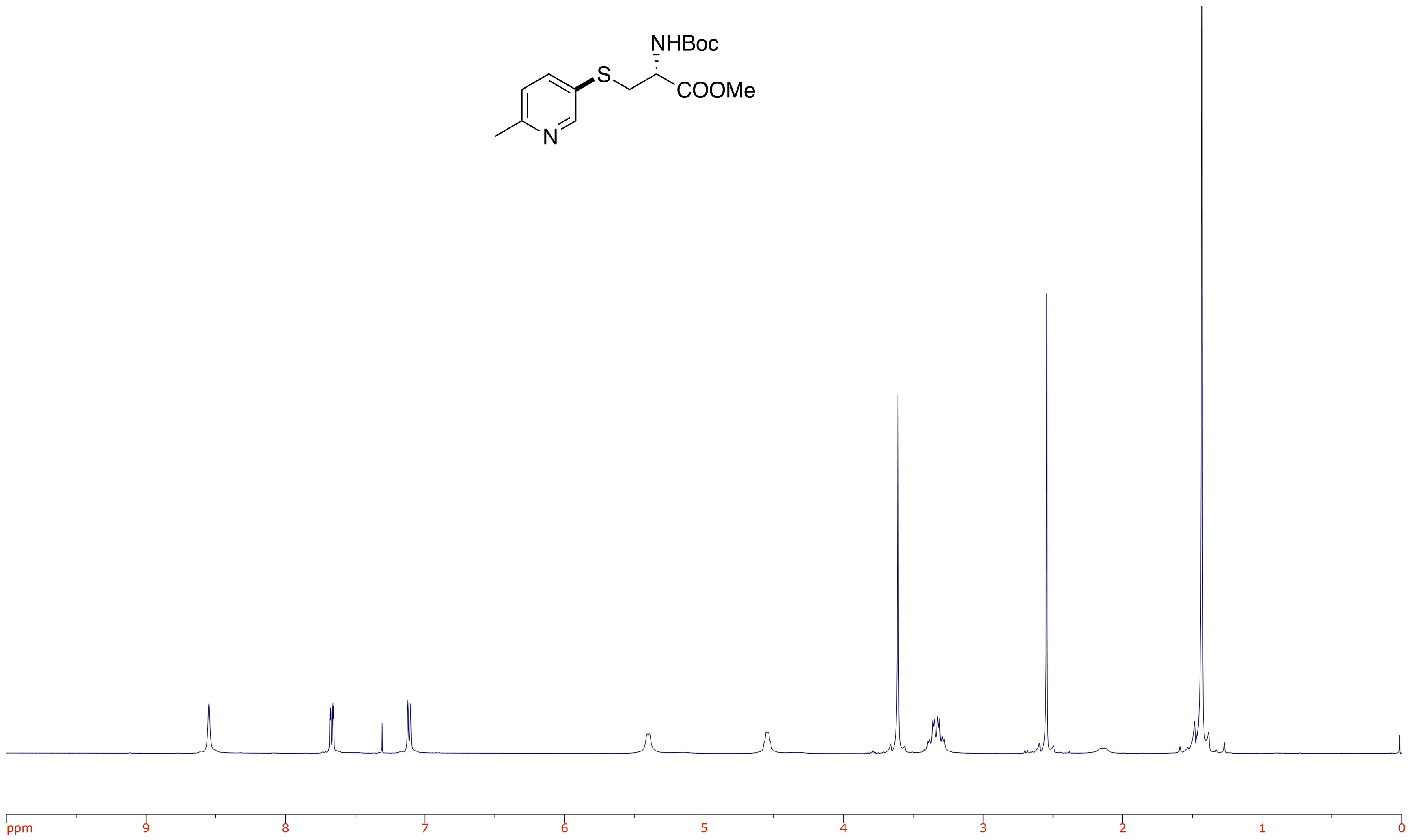
NHBoc
S
COOMe
N
ppm
9
8
7
6
5
4
3
2
1
0

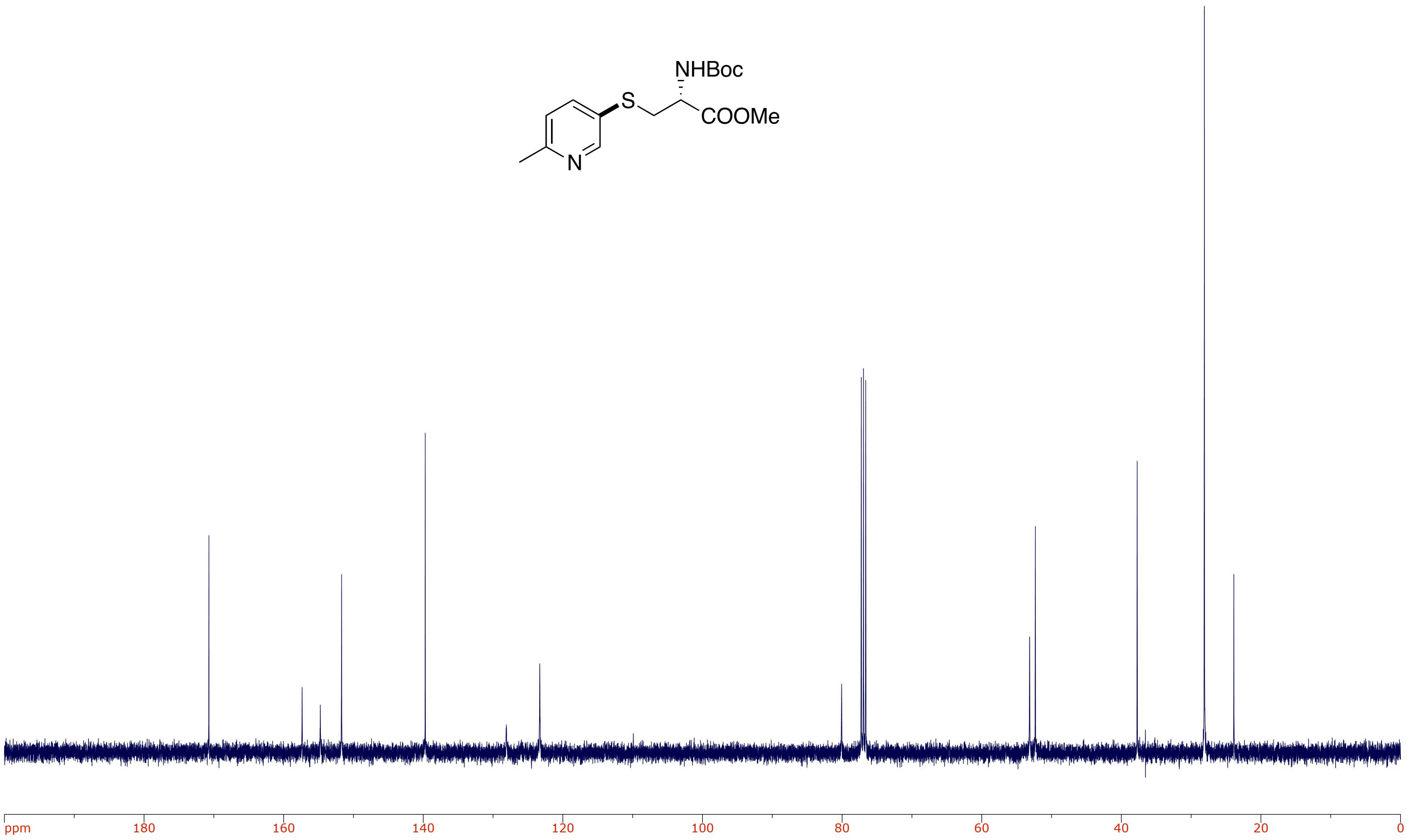
NHBoc
S
COOMe
N
ppm
180
160
140
120
100
80
60
40
20
0

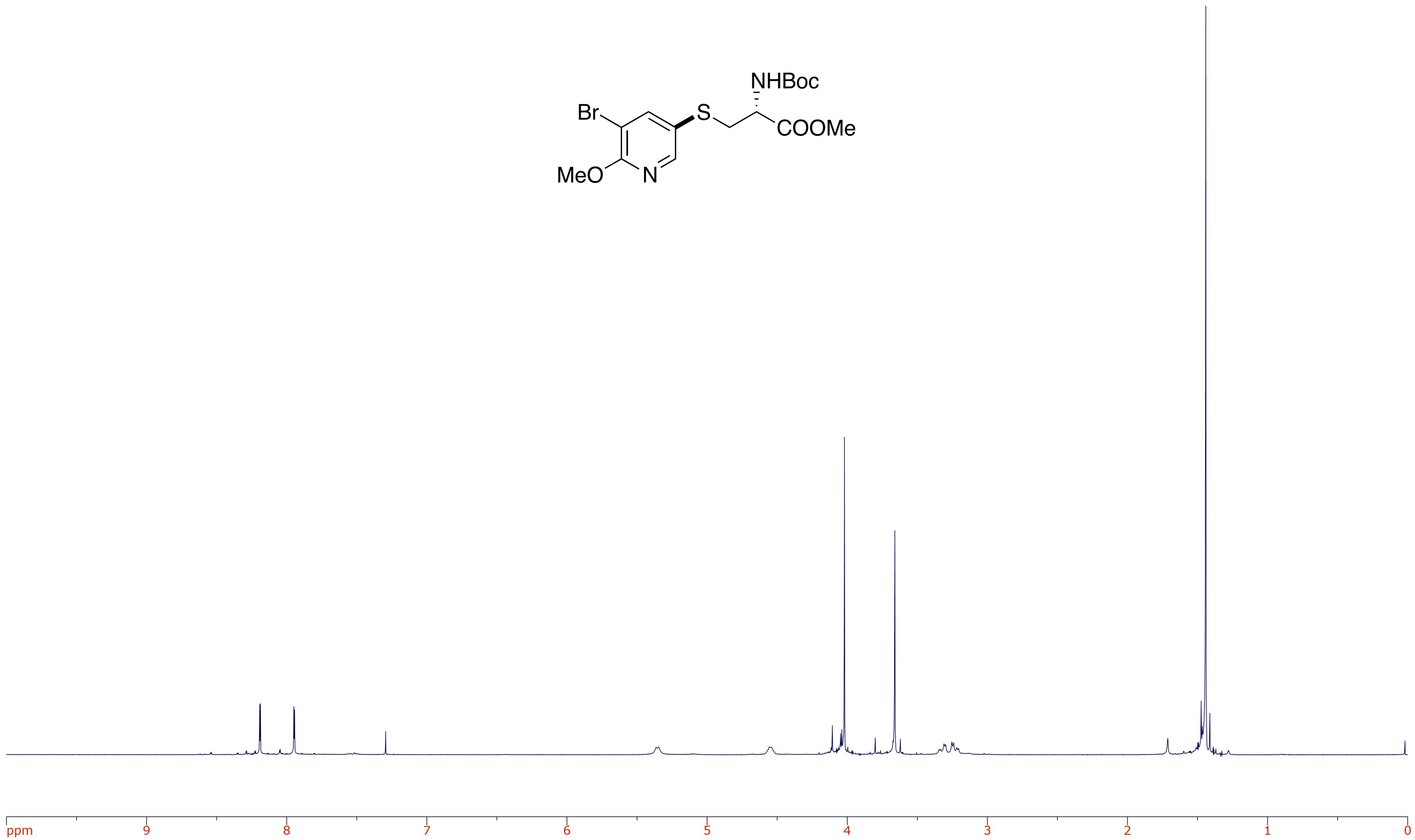
NHBoc
Br
S
COOMe
MeO
N
ppm
9
8
7
6
5
4
3
2
1
0

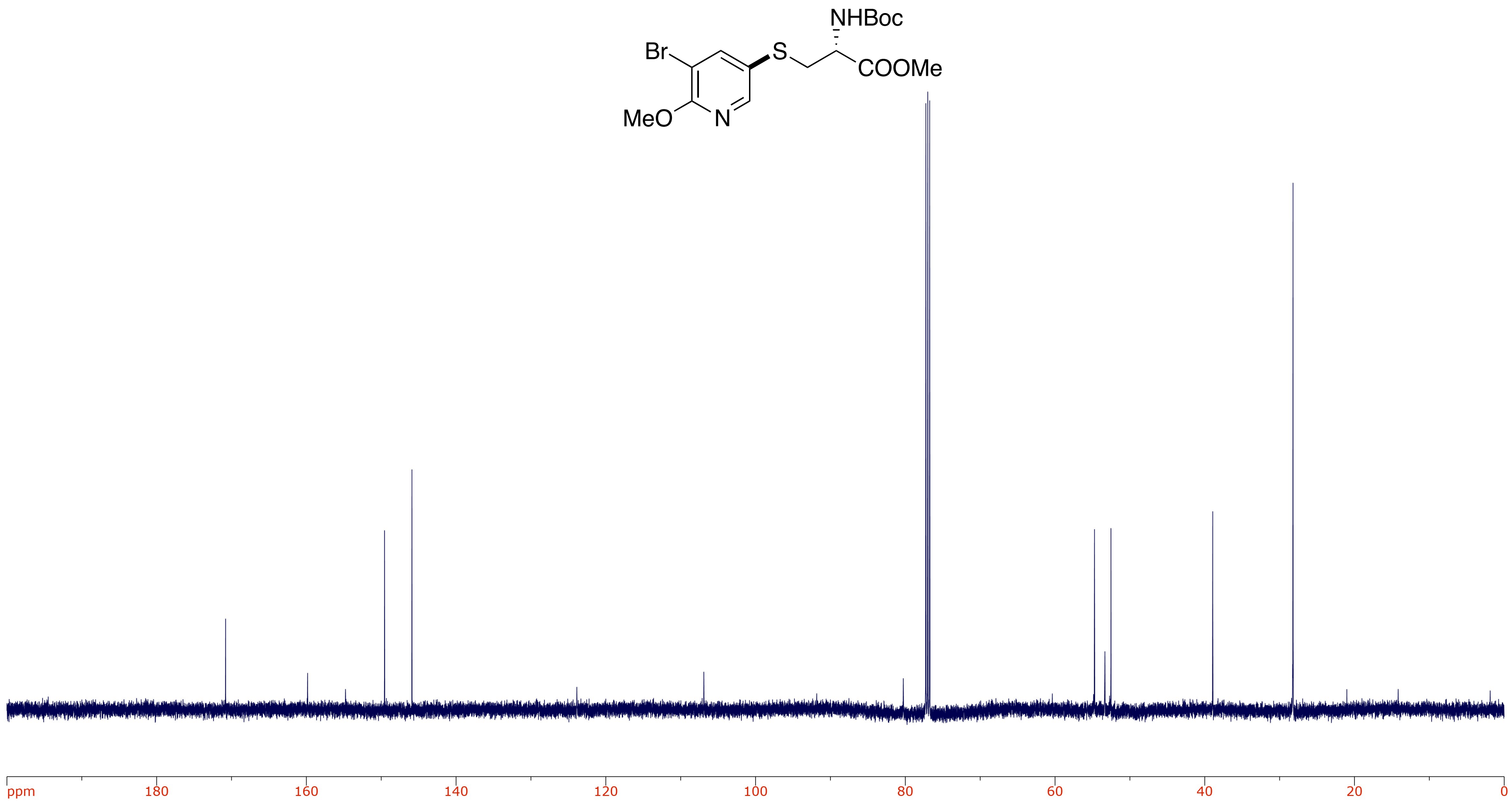
NHBoc
Br
S
COOMe
MeO
N
ppm
180
160
140
120
100
80
60
40
20
0

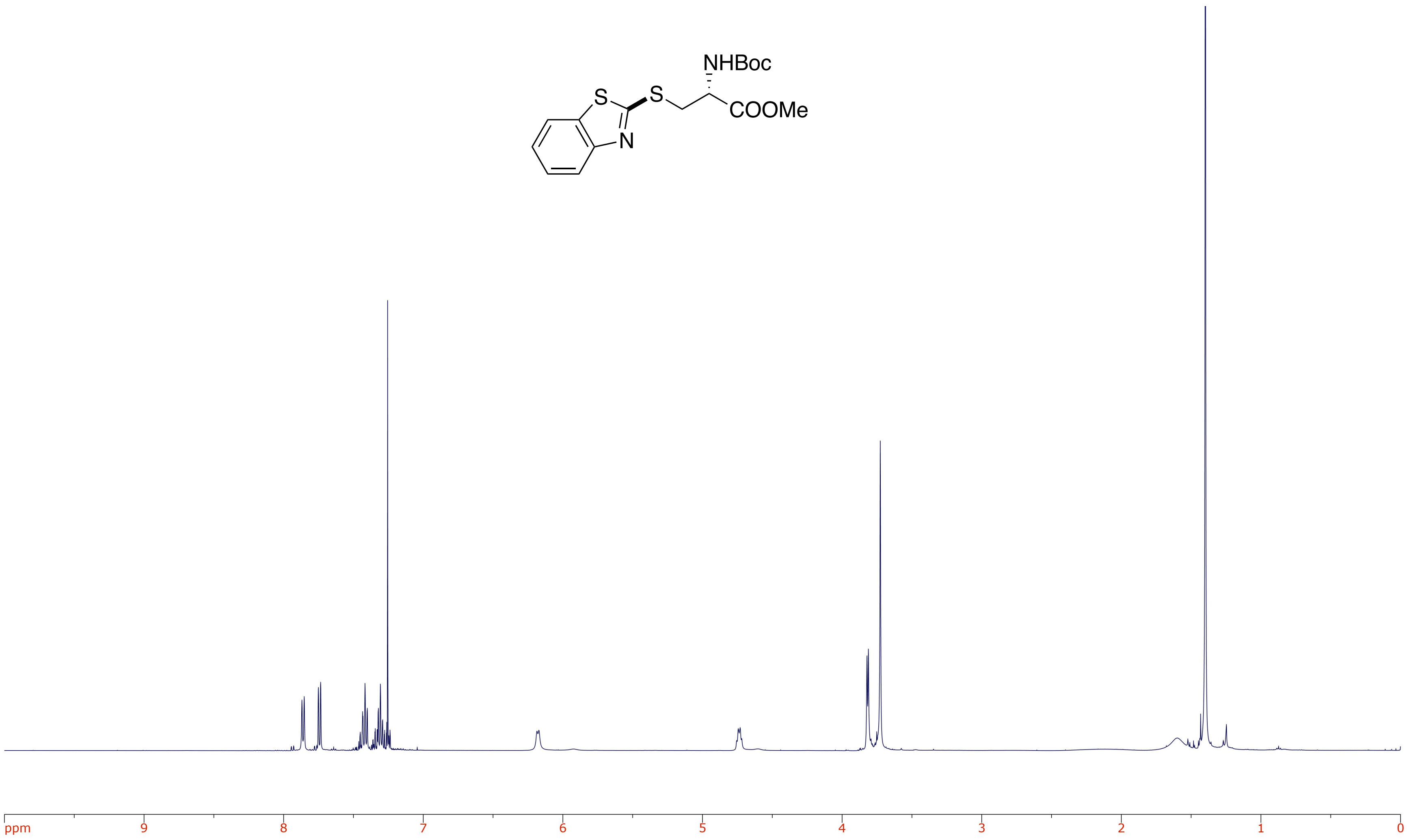
NHBoc
S
S
N
COOMe
ppm
9
8
7
6
5
4
3
2
1
0

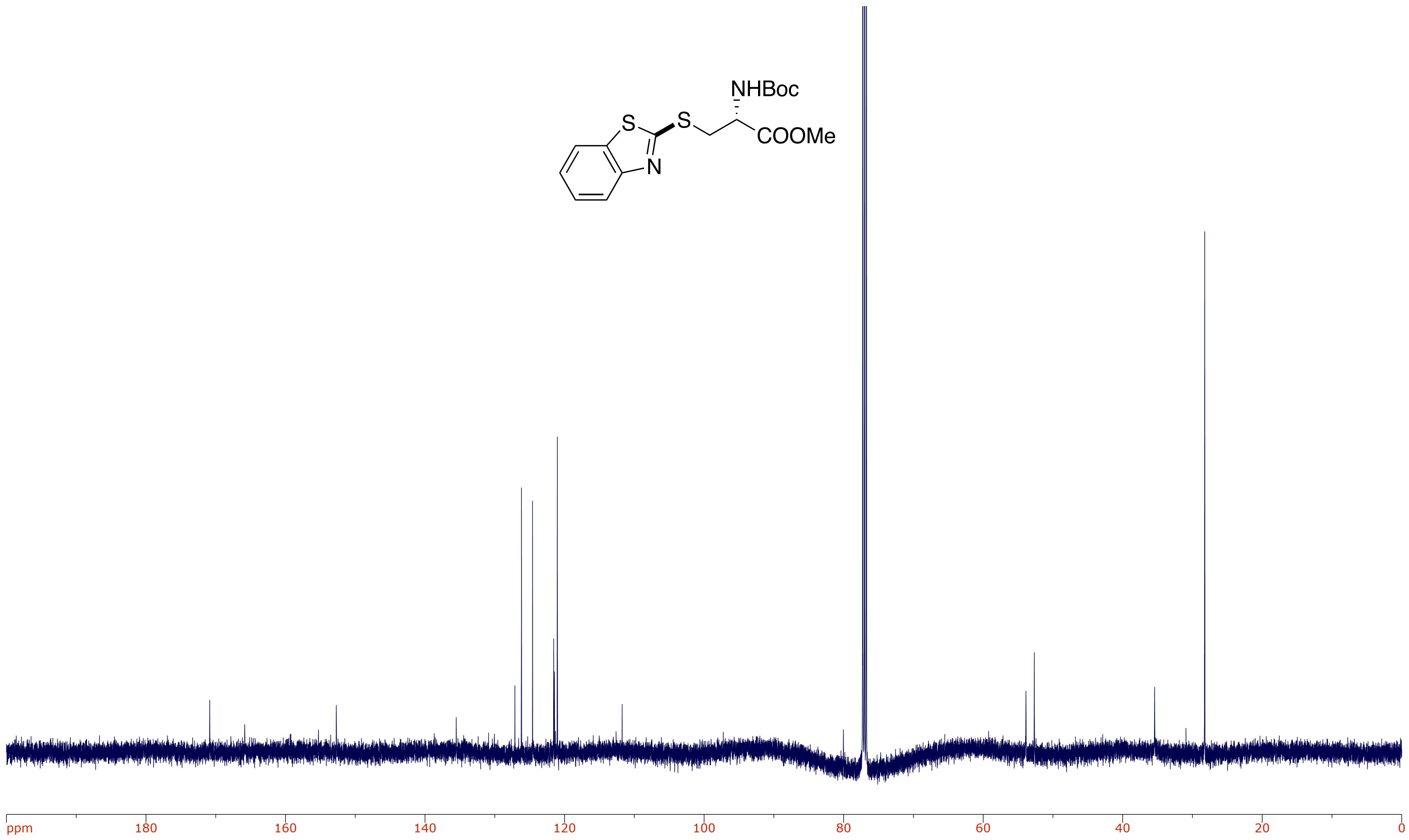
NHBoc
S
S
N
COOMe
ppm
180
160
140
120
100
80
60
40
20
0

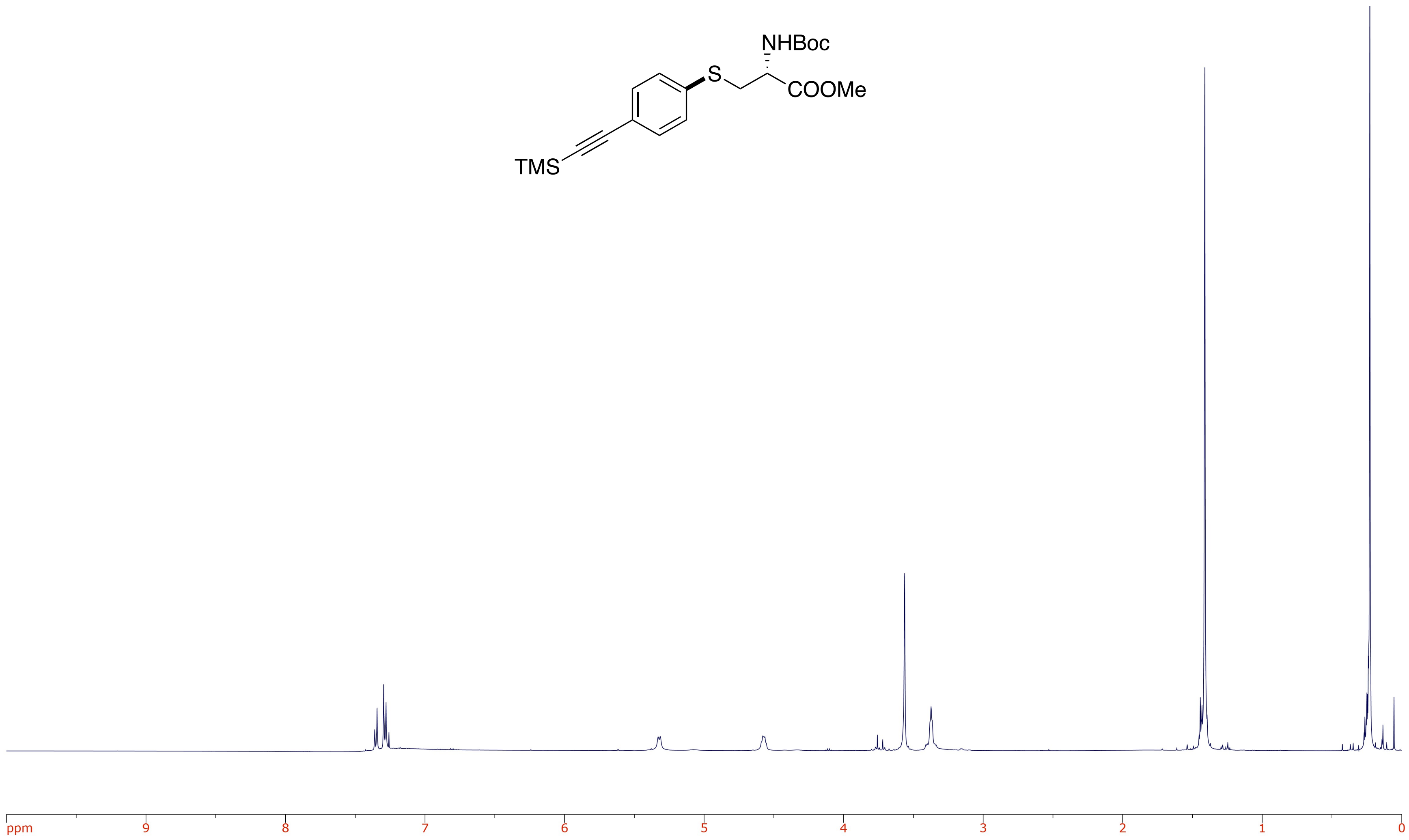
NHBoc
S
COOMe
TMS
ppm
9
8
7
6
5
4
3
2
1
0

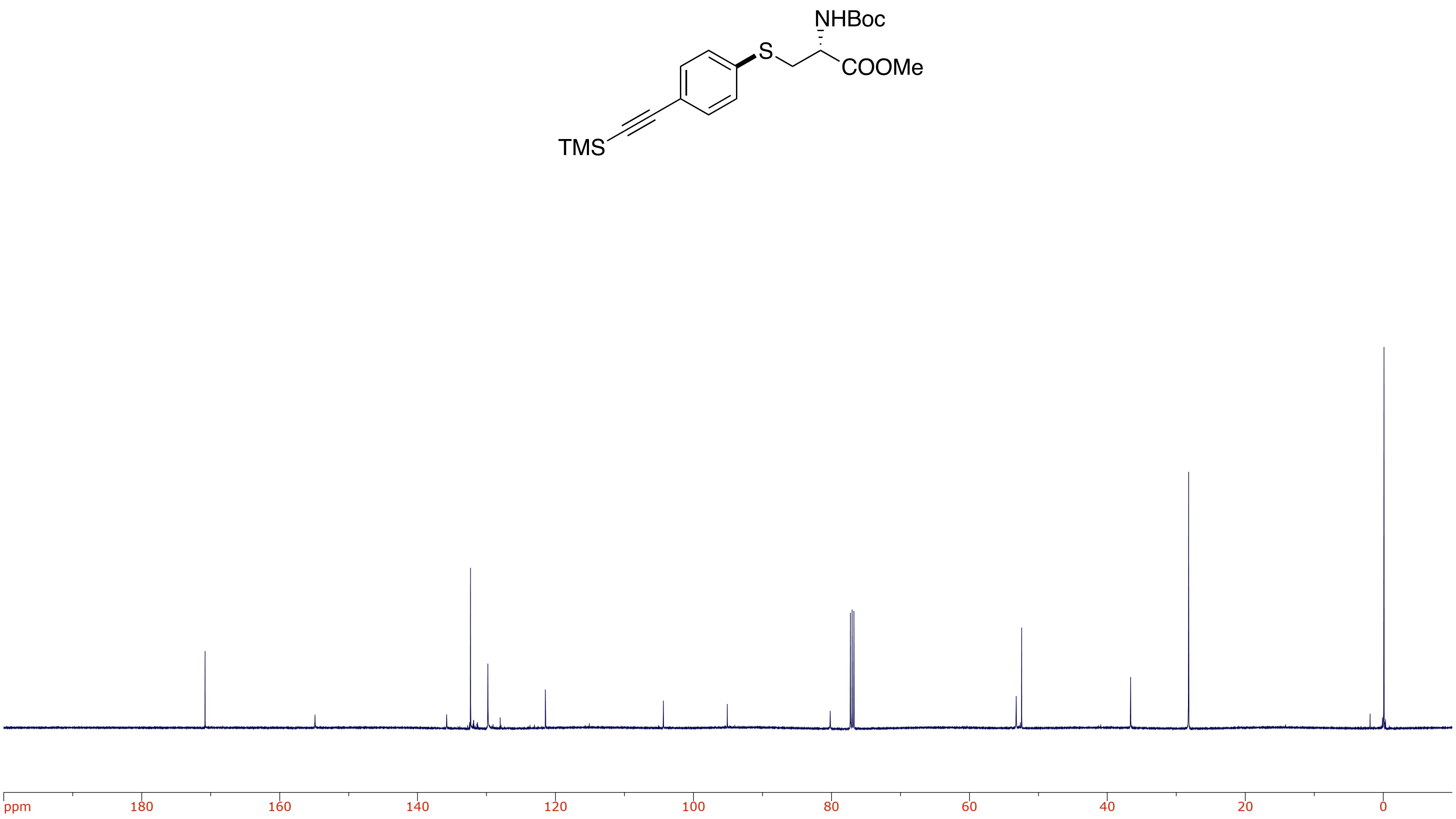

NHBoc
S
COOMe
TMS
ppm
180
160
140
120
100
80
60
40
20
0

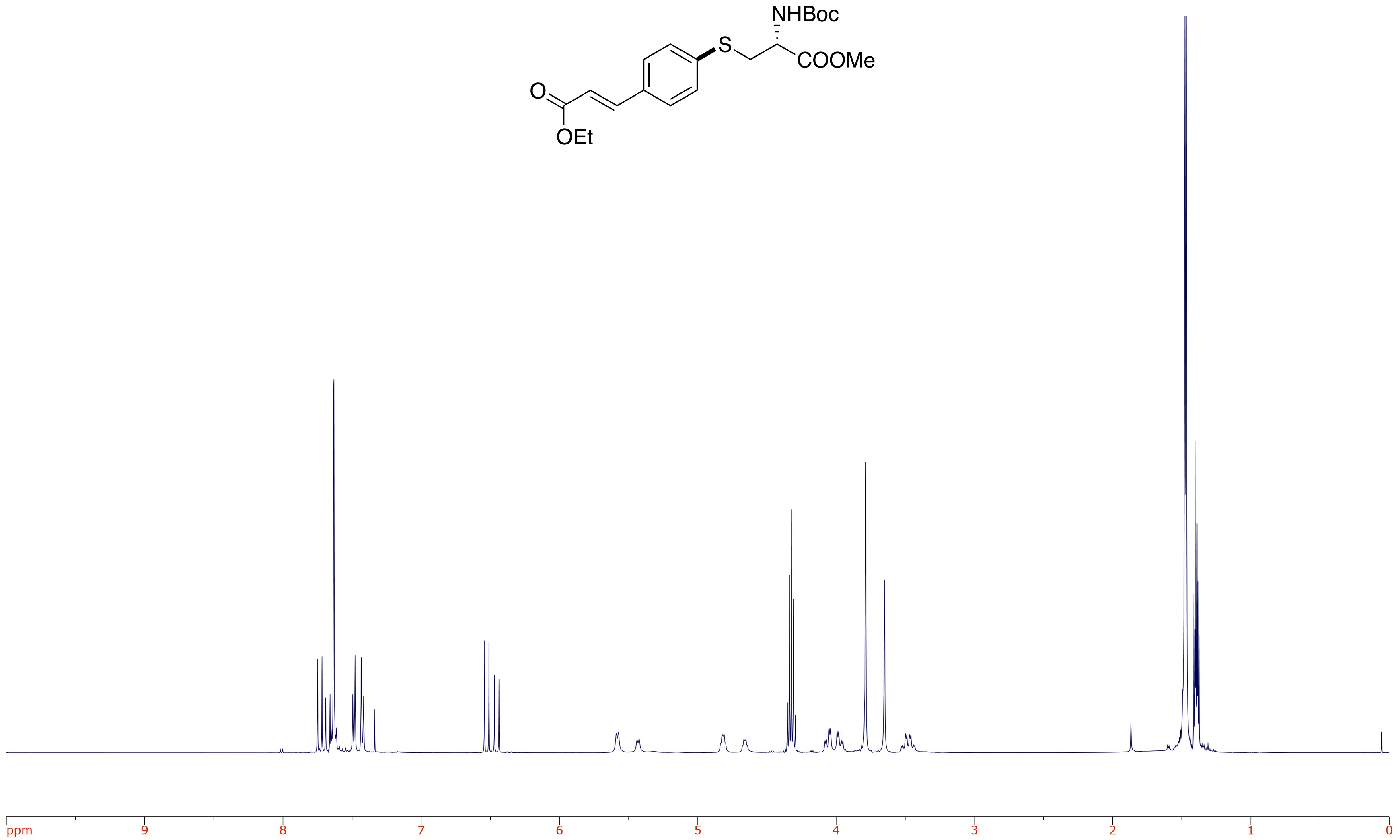
NHBoc
S
COOMe
O
OEt
ppm
9
8
7
6
5
4
3
2
1
0

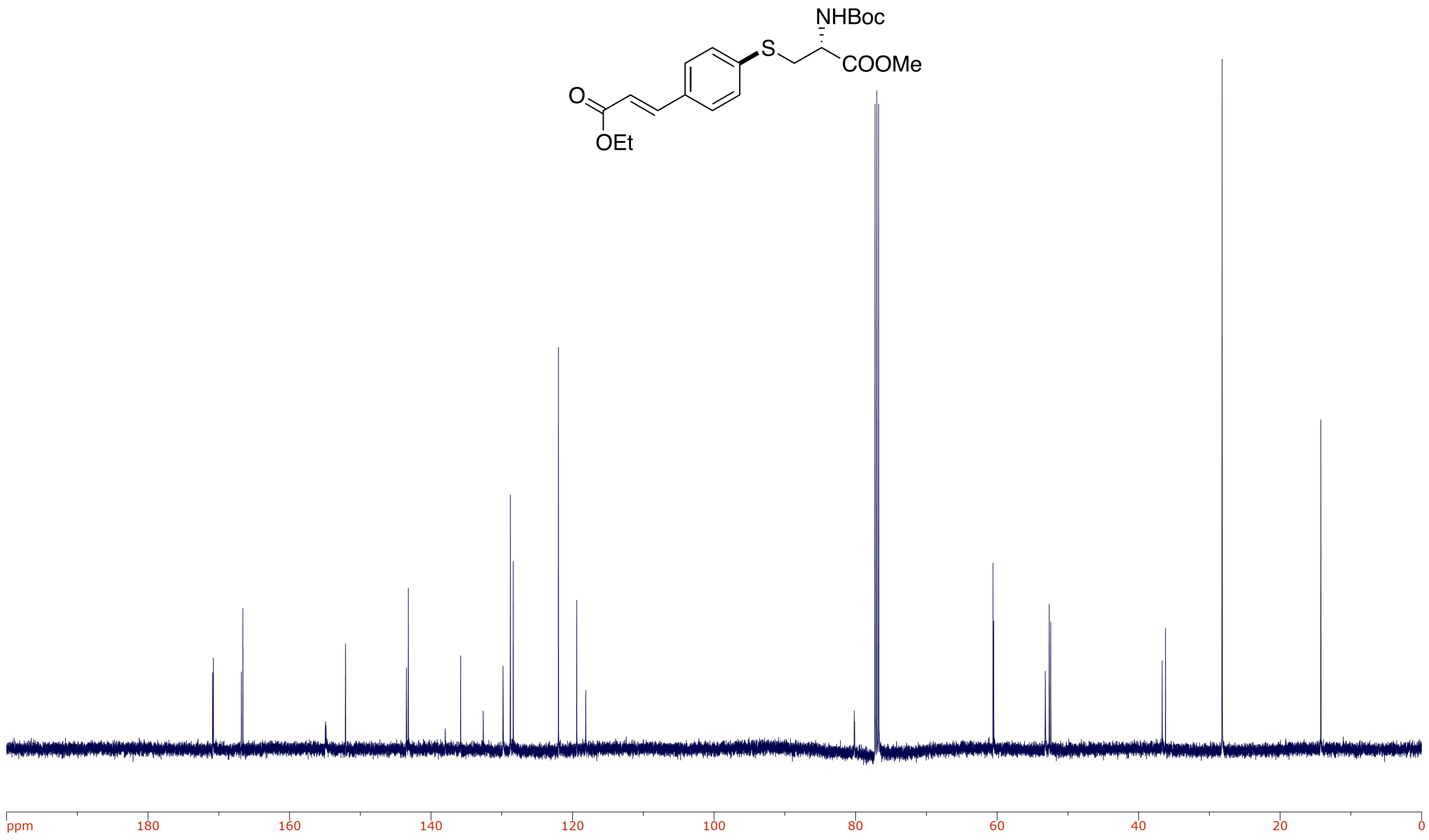

NHBoc
S
COOMe
O
OEt
ppm
180
160
140
120
100
80
60
40
20
0

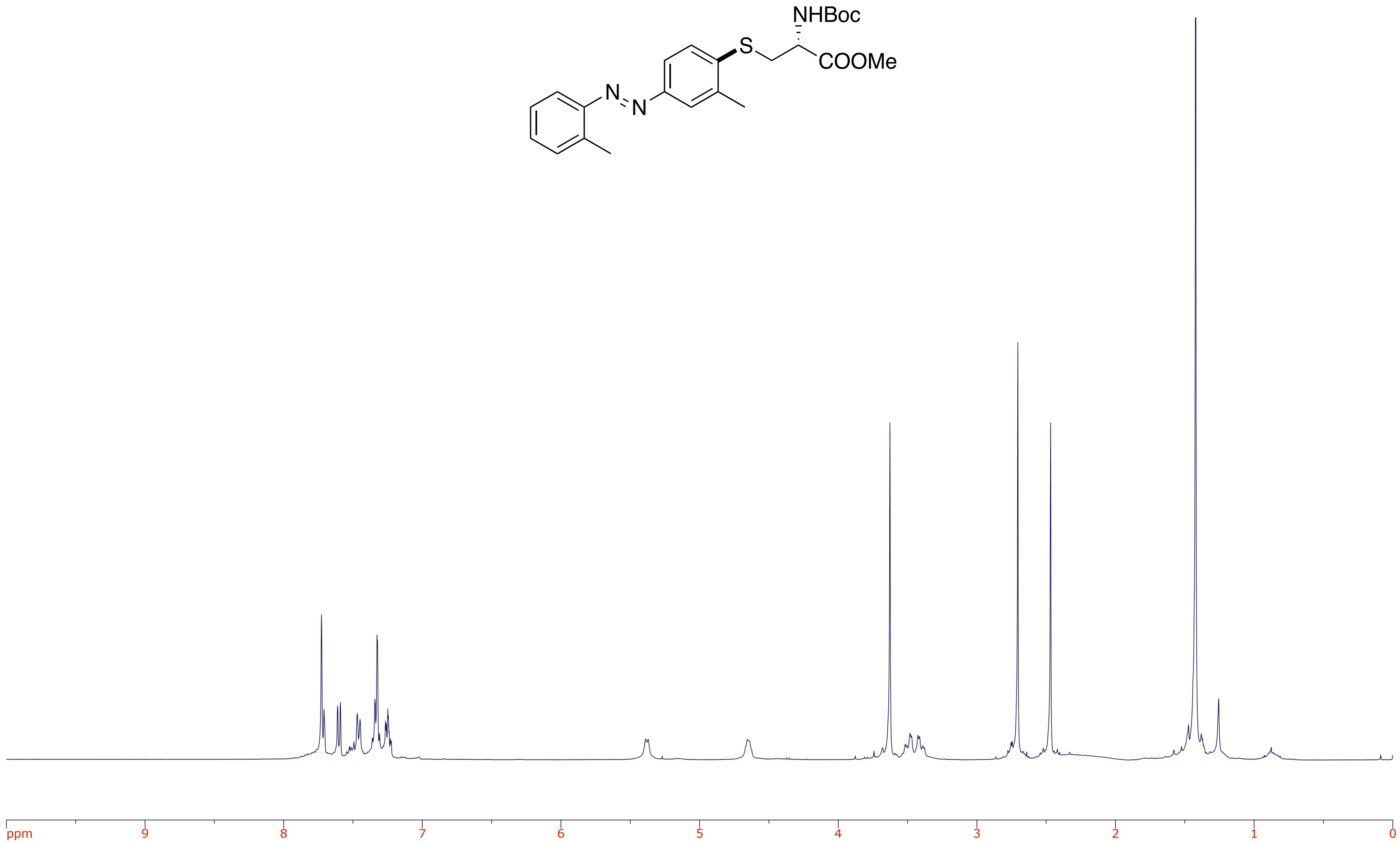
NHBoc
S
COOMe
N
N
ppm
9
8
7
6
5
4
3
2
1
0

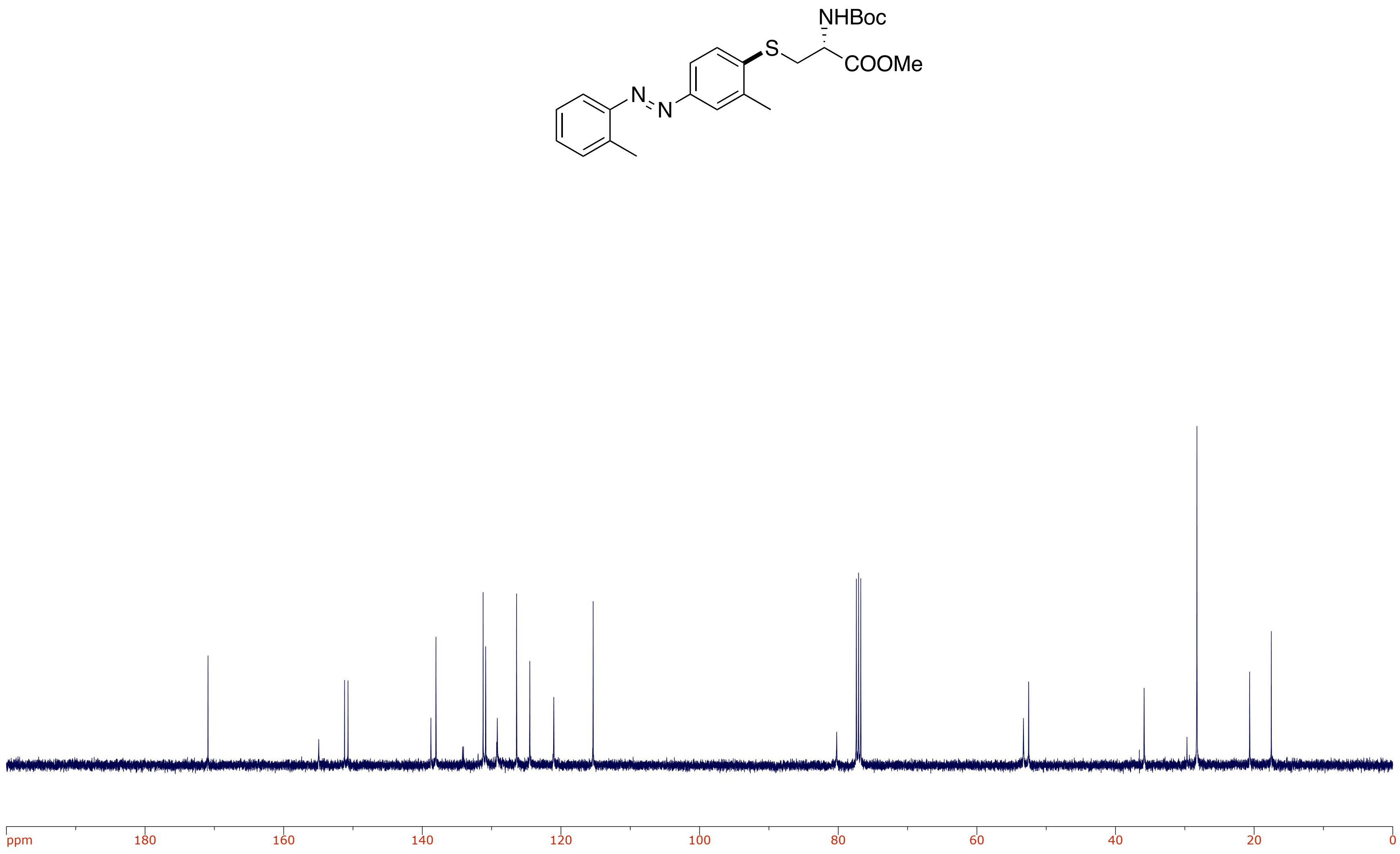
NHBoc
S
COOMe
N
N
ppm
180
160
140
120
100
80
60
40
20
0

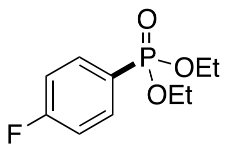
O
P
OEt
OEt
F

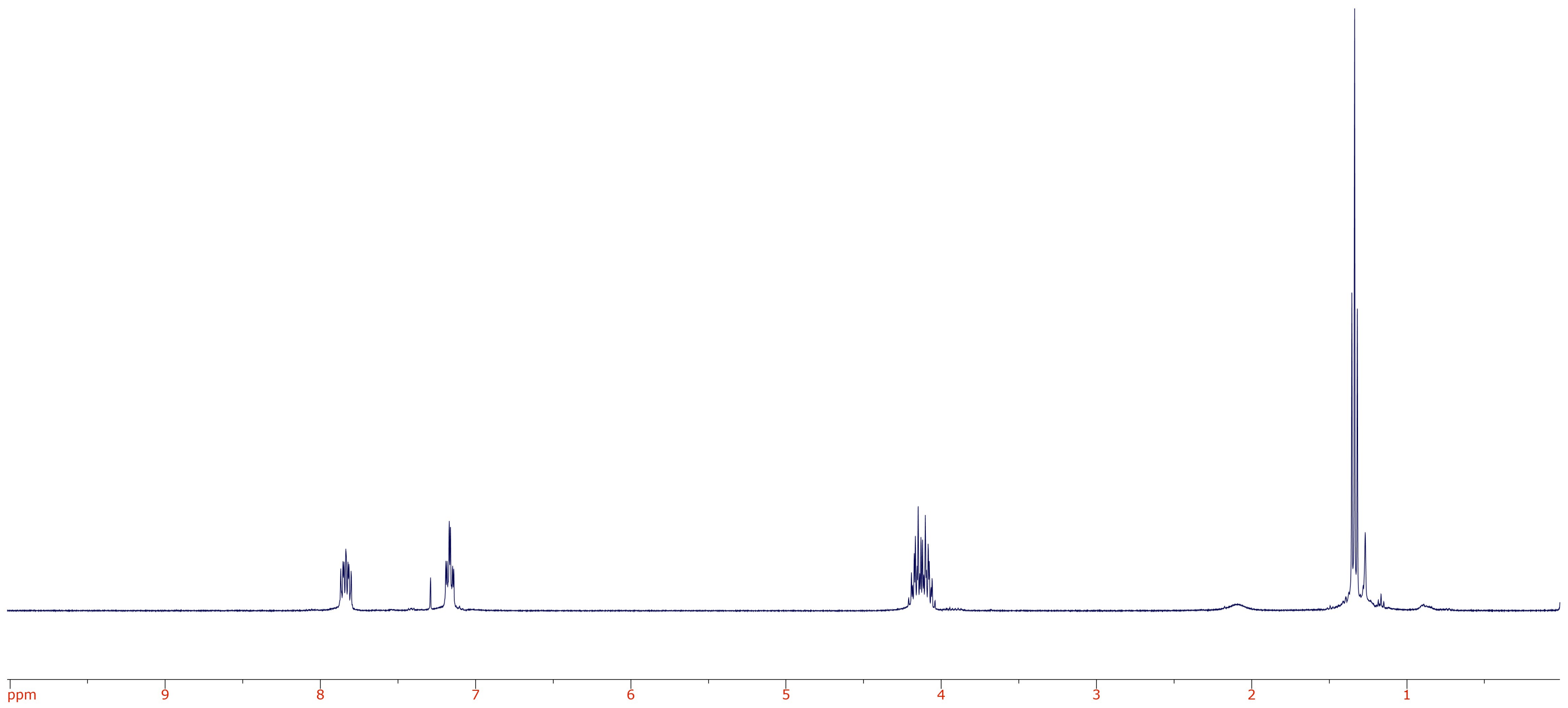
ppm
9
8
7
6
5
4
3
2
1

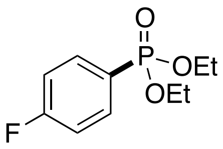

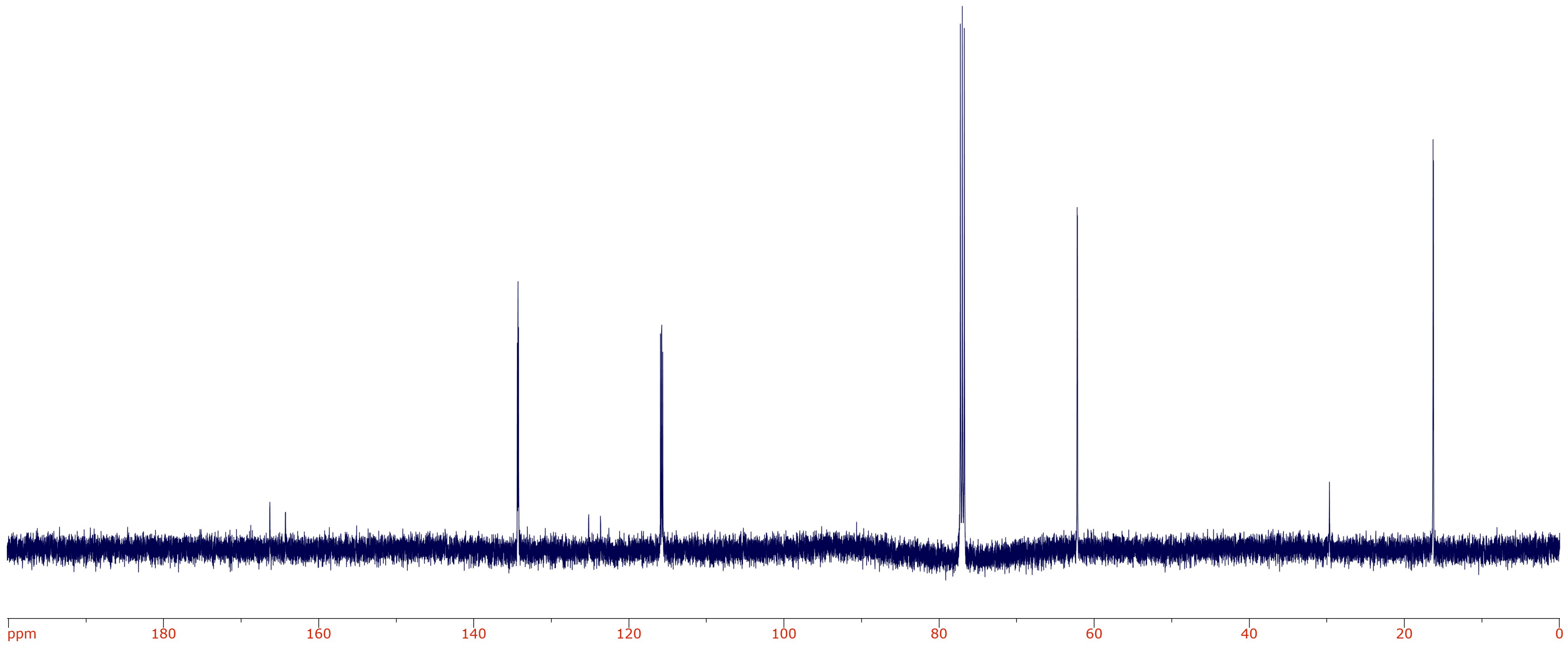
ppm
180
160
140
120
100
80
60
40
20
0

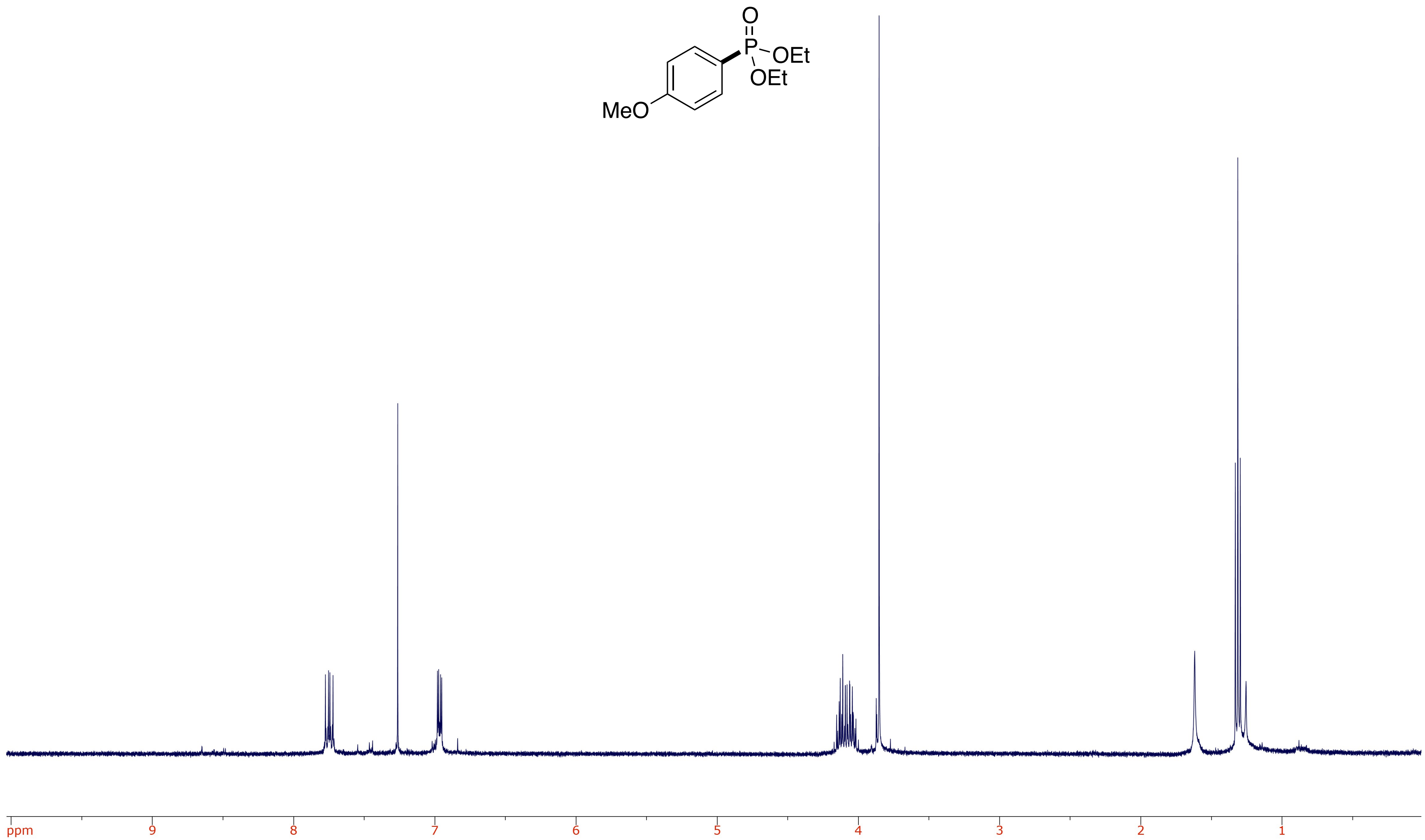
MeO
O
P
OEt
OEt
ppm
9
8
7
6
5
4
3
2
1

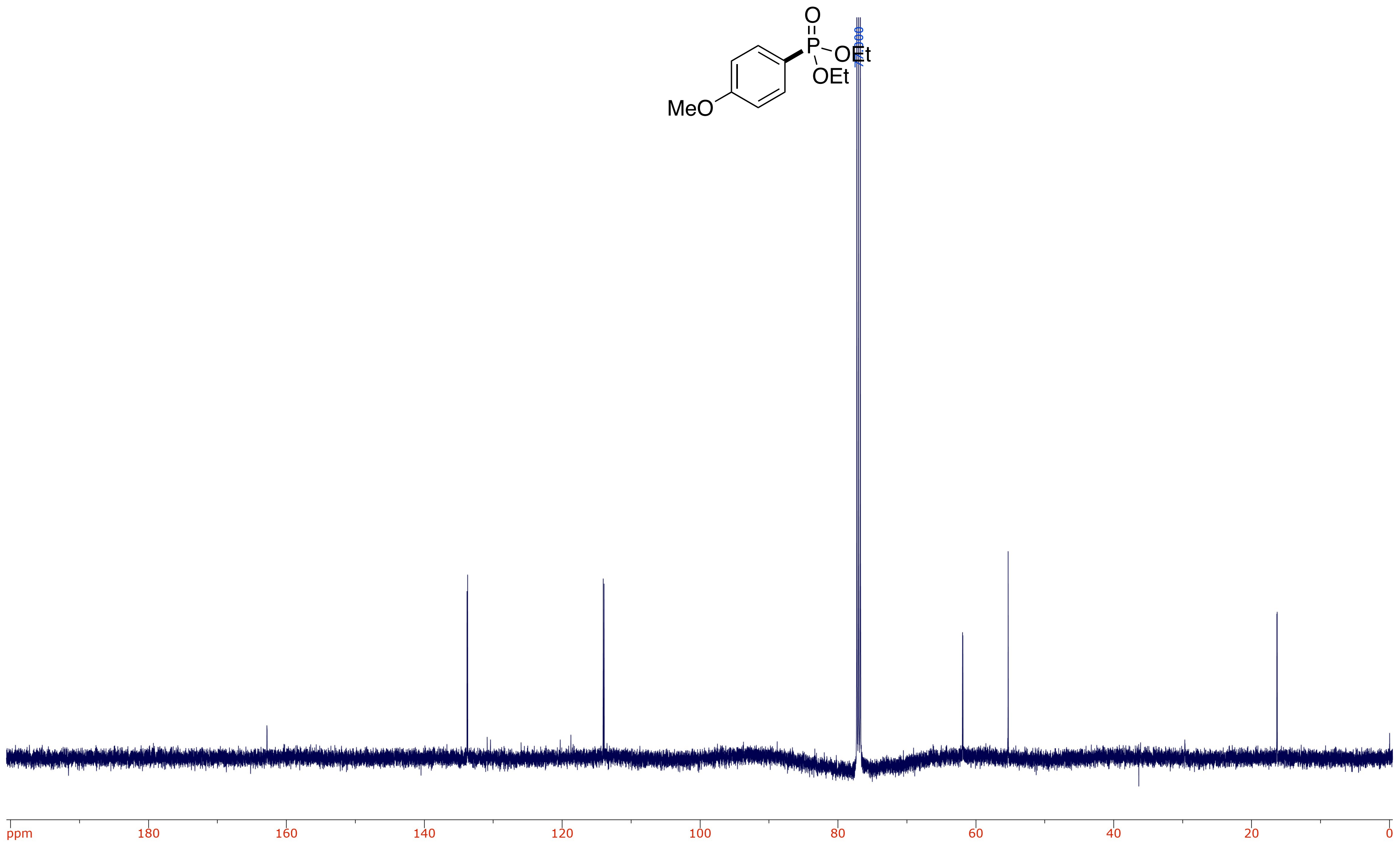
O
P
OEt
OEt
MeO
ppm
180
160
140
120
100
80
60
40
20
0

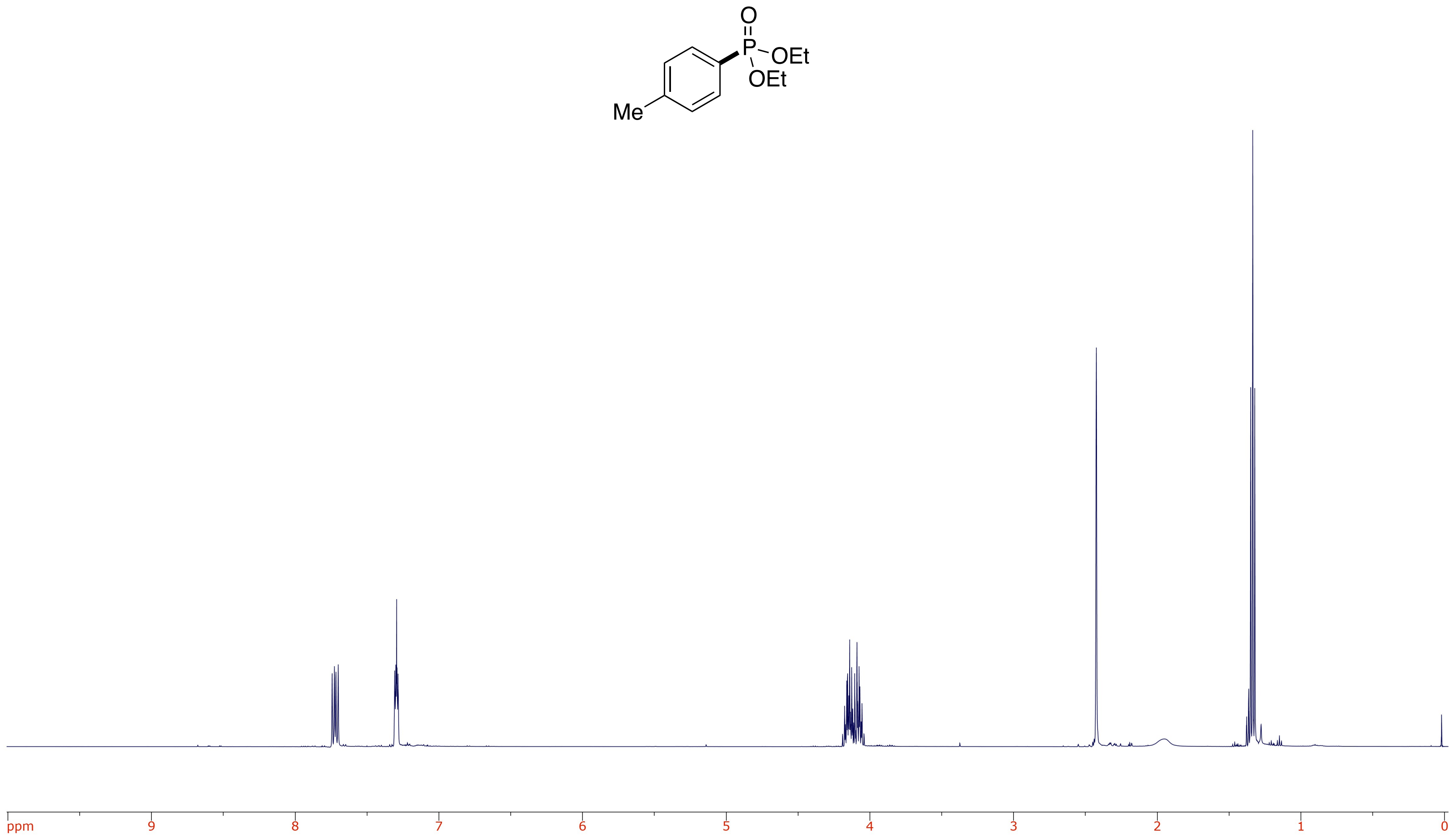
O
P
OEt
OEt
Me
ppm
9
8
7
6
5
4
3
2
1
0

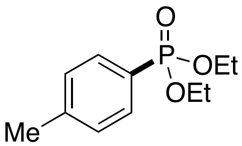
O
P
OEt
OEt
Me

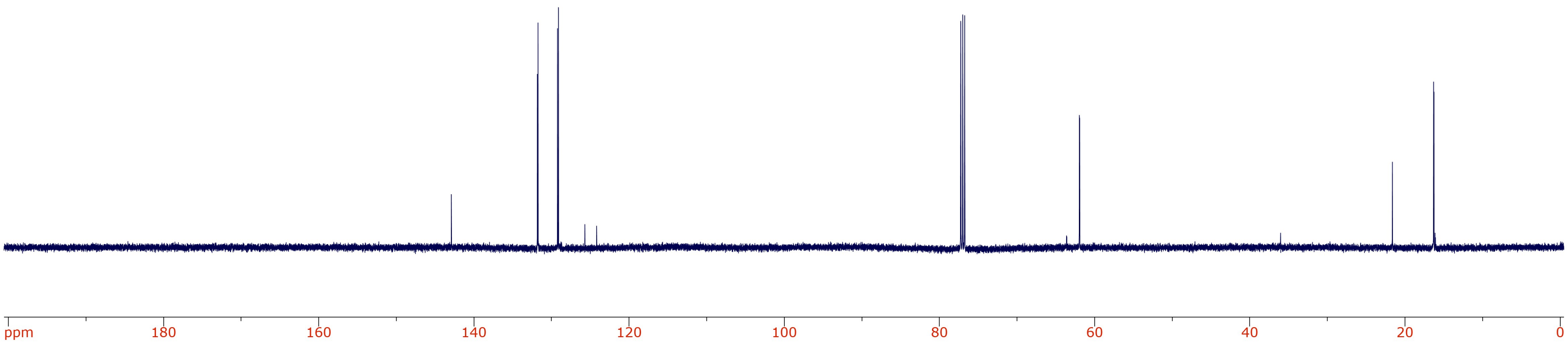
ppm
180
160
140
120
100
80
60
40
20
0

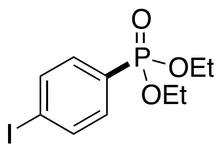
O
P
OEt
OEt
I

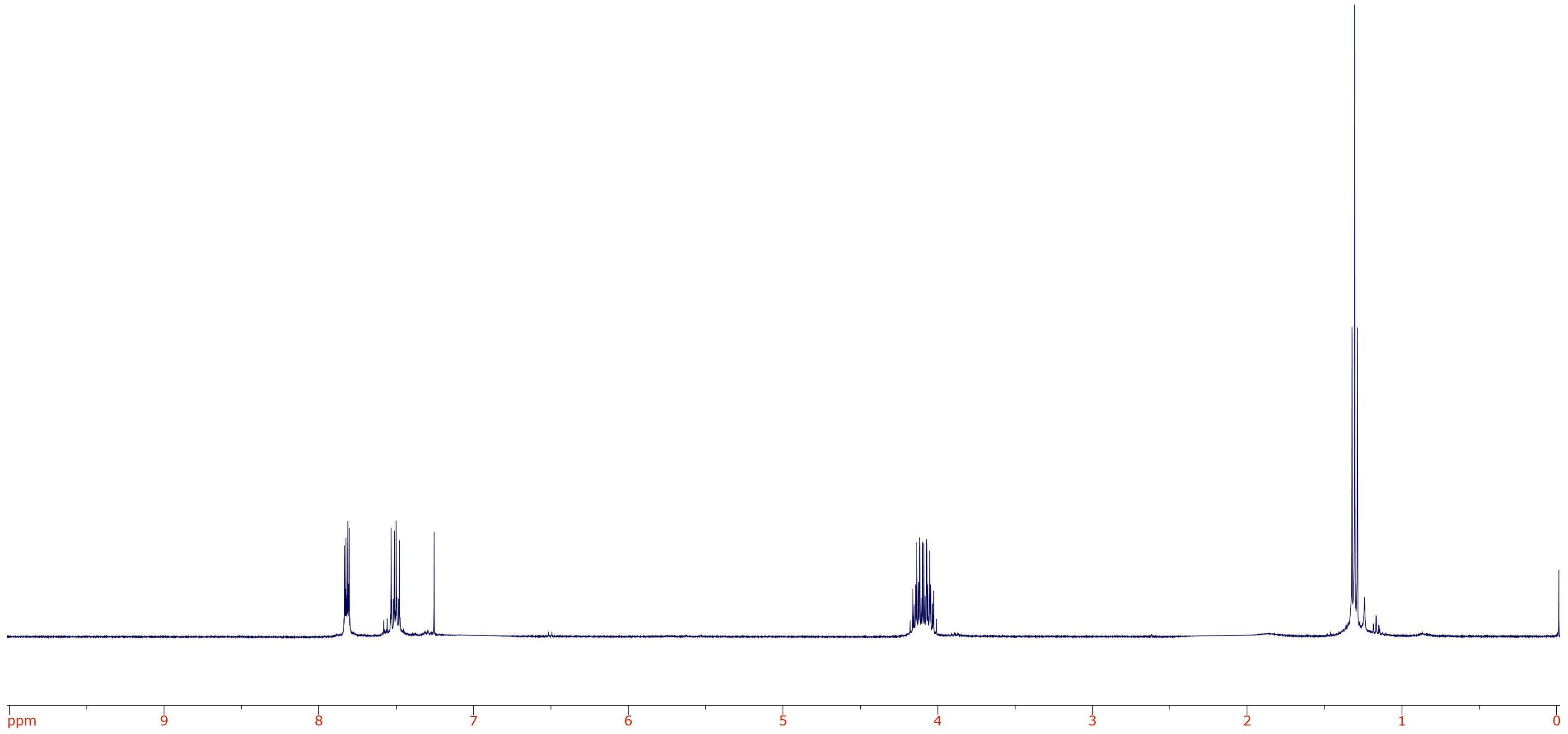
ppm
9
8
7
6
5
4
3
2
1
0

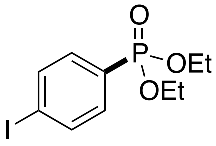
O
P
OEt
OEt
I

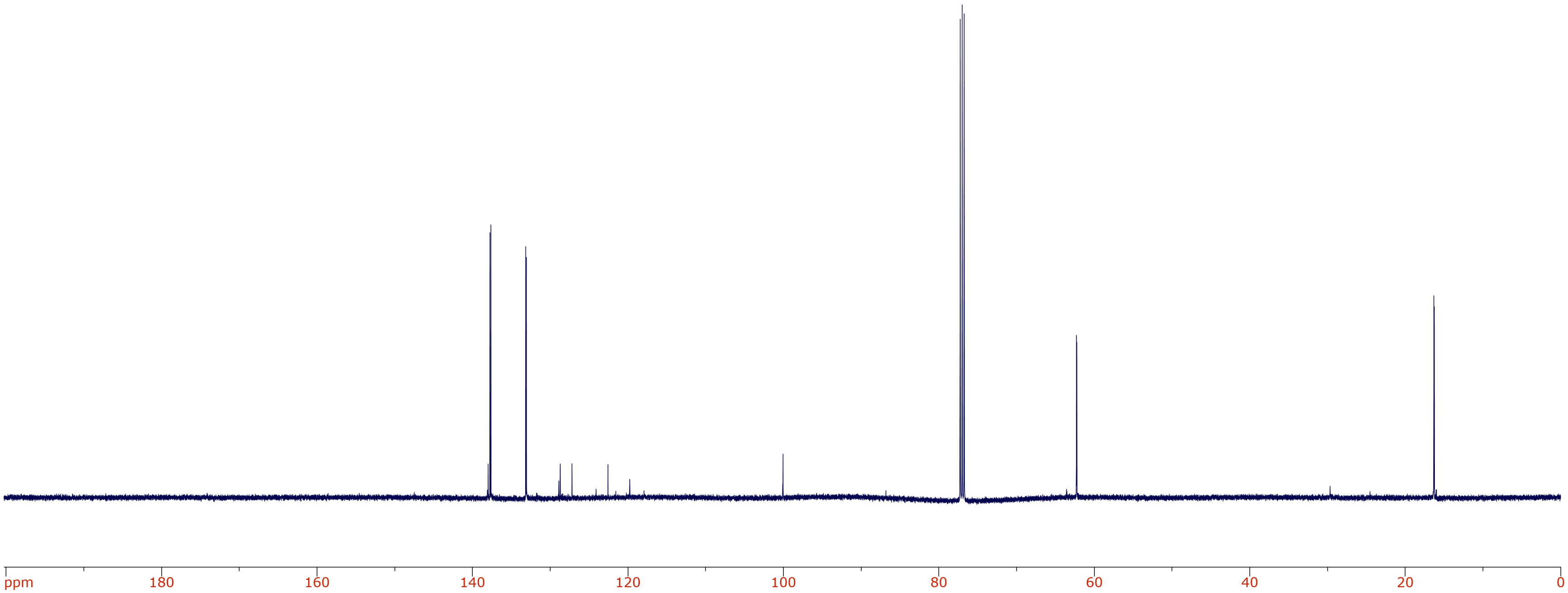
ppm
180
160
140
120
100
80
60
40
20
0

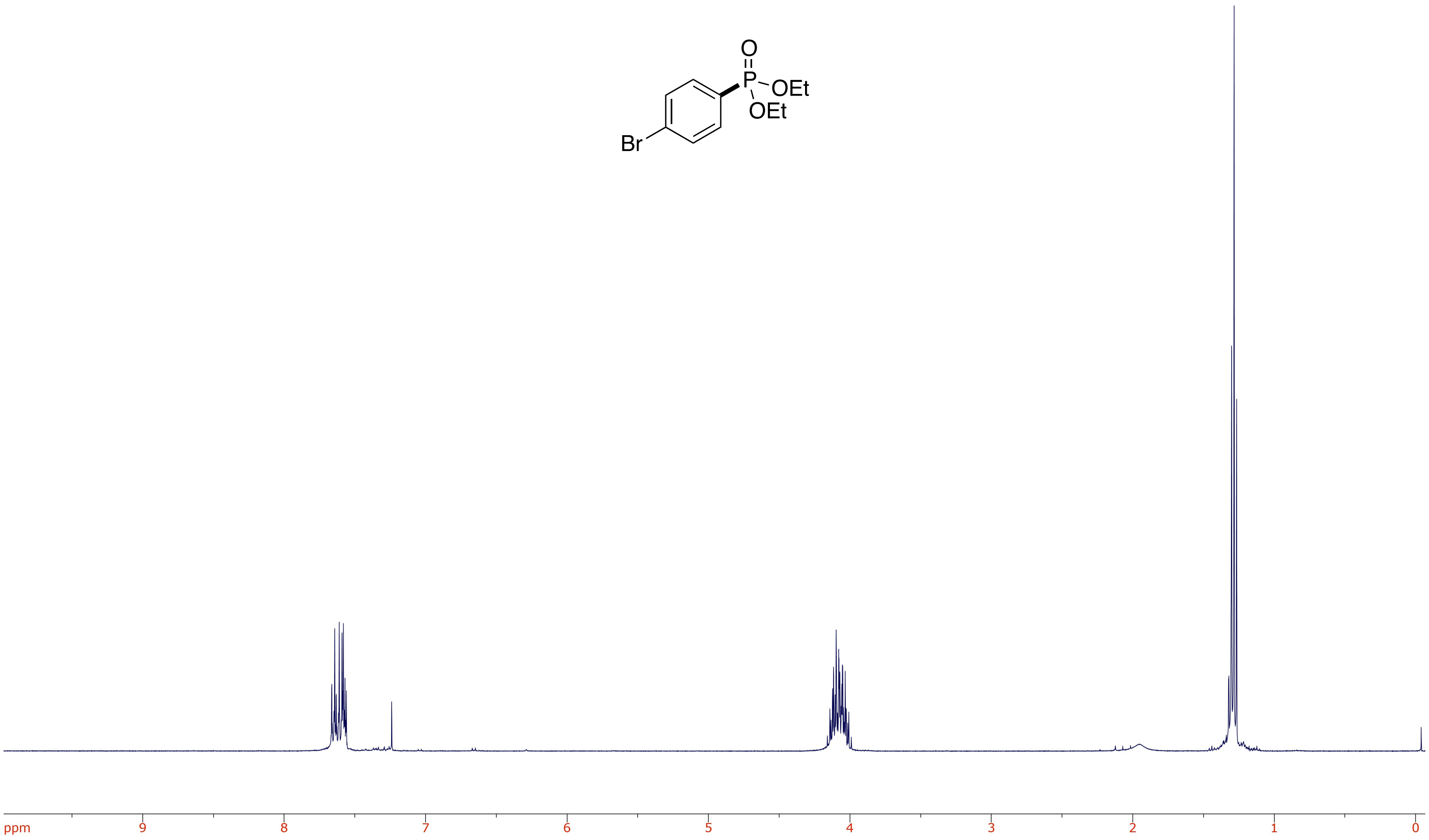
O
P
OEt
OEt
Br
ppm
9
8
7
6
5
4
3
2
1
0

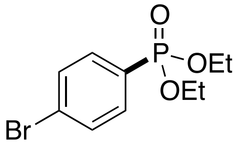
O
P
OEt
OEt
Br

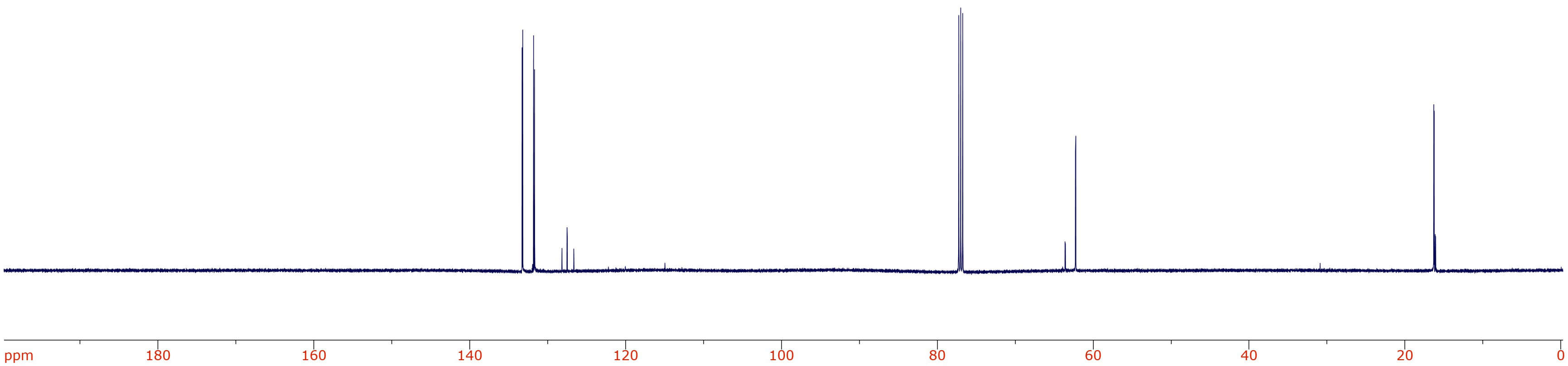
ppm
180
160
140
120
100
80
60
40
20
0

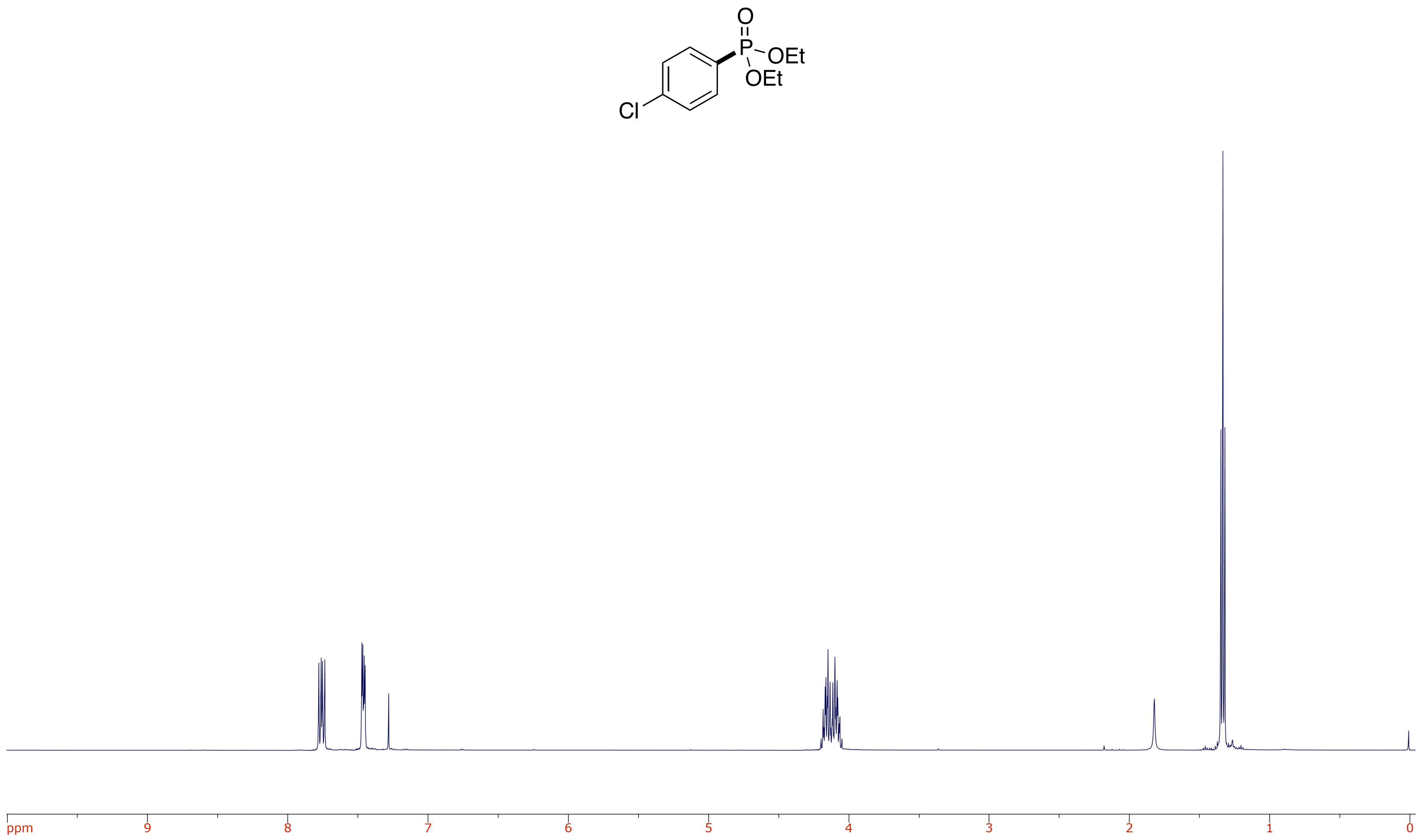
O
P
OEt
OEt
Cl
ppm
9
8
7
6
5
4
3
2
1
0

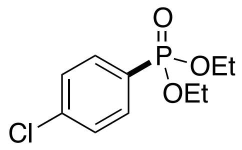
O
P
OEt
OEt
Cl

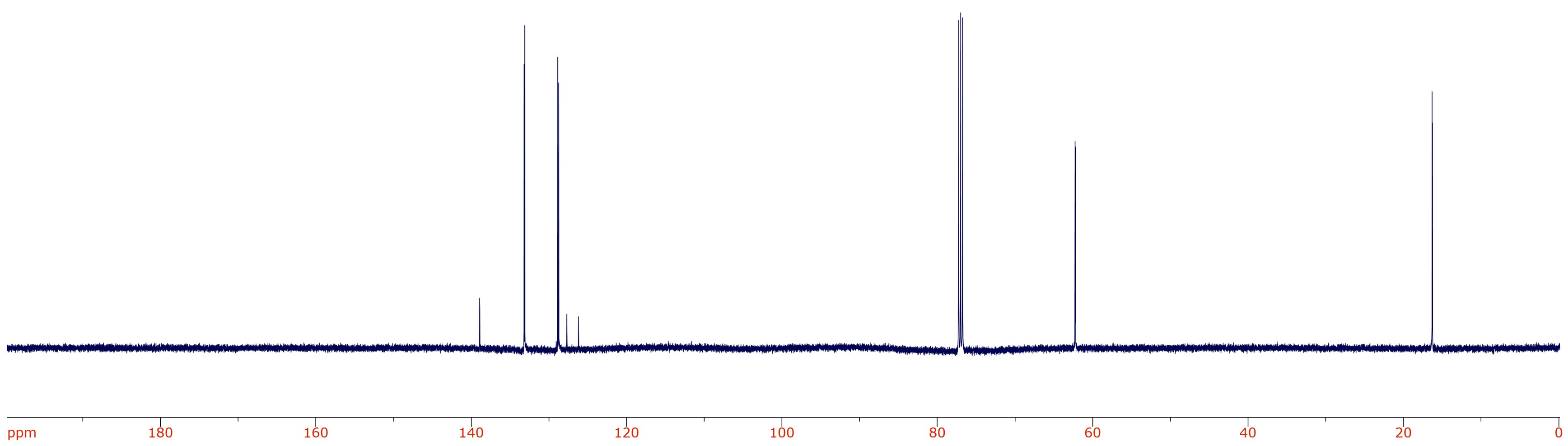
ppm
180
160
140
120
100
80
60
40
20
0

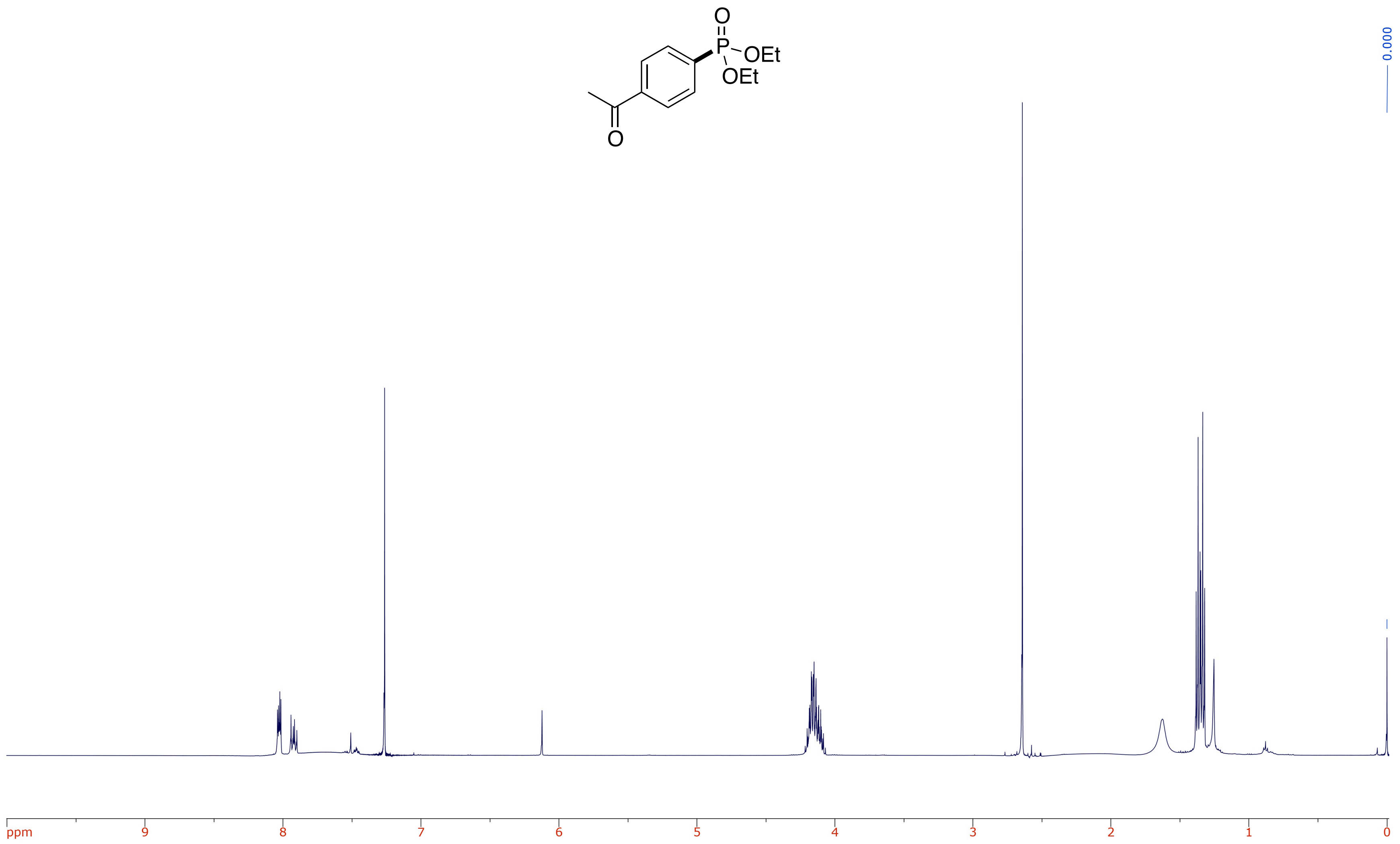

O
P
OEt
OEt
O
0.000
ppm
9
8
7
6
5
4
3
2
1
0

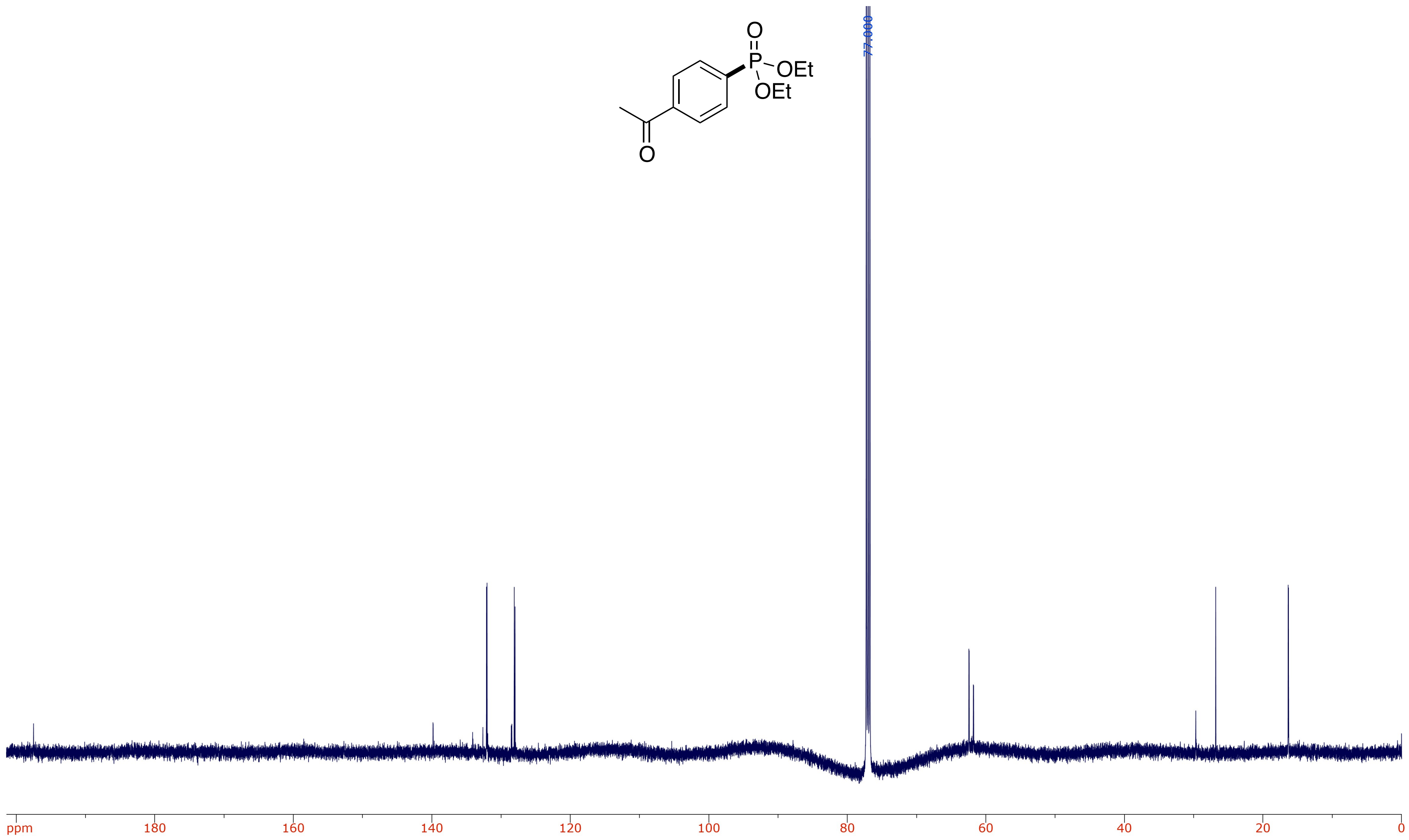
O
P
OEt
OEt
O
77.000
ppm
180
160
140
120
100
80
60
40
20
0

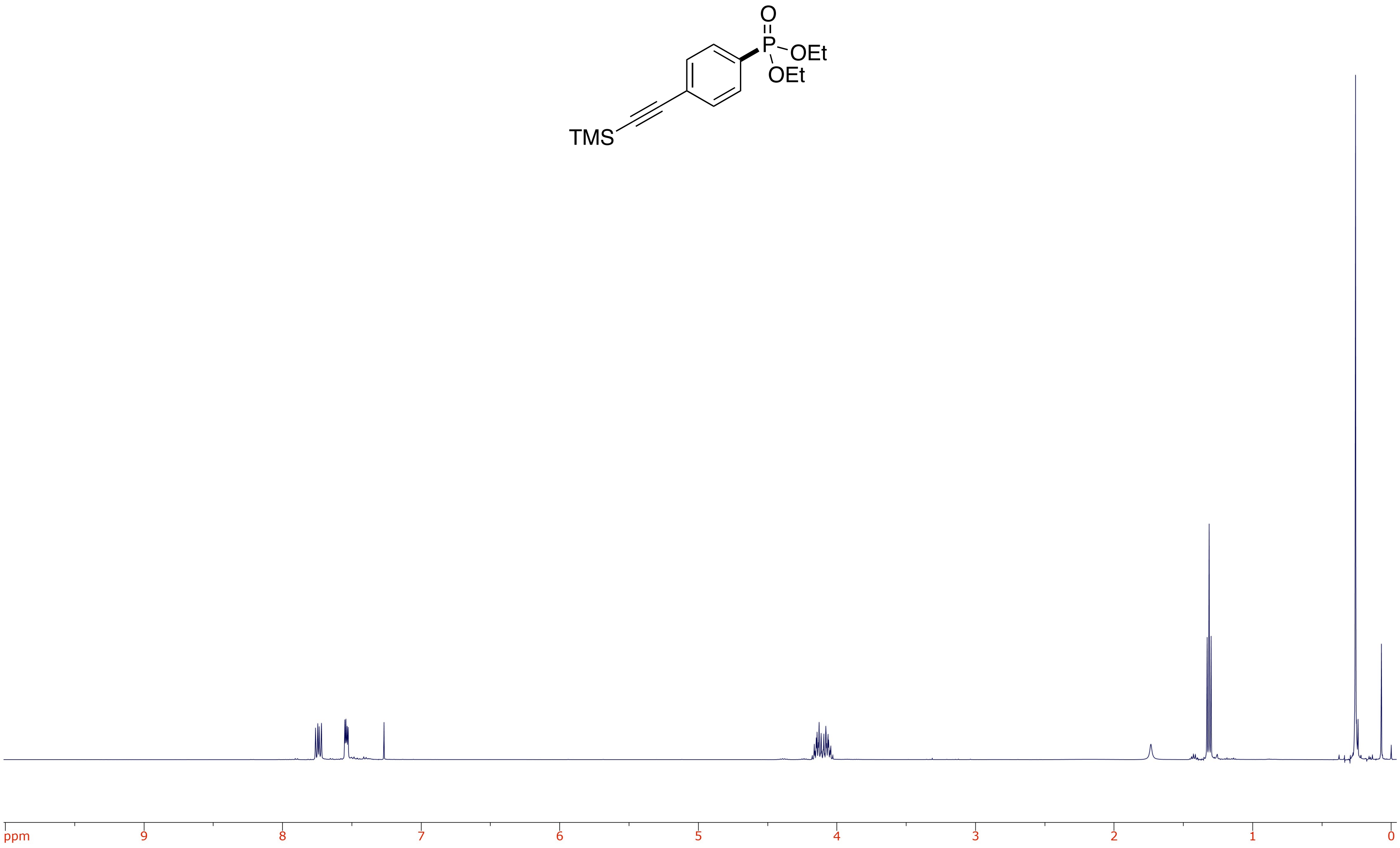

O
P
OEt
OEt
TMS
ppm
9
8
7
6
5
4
3
2
1
0

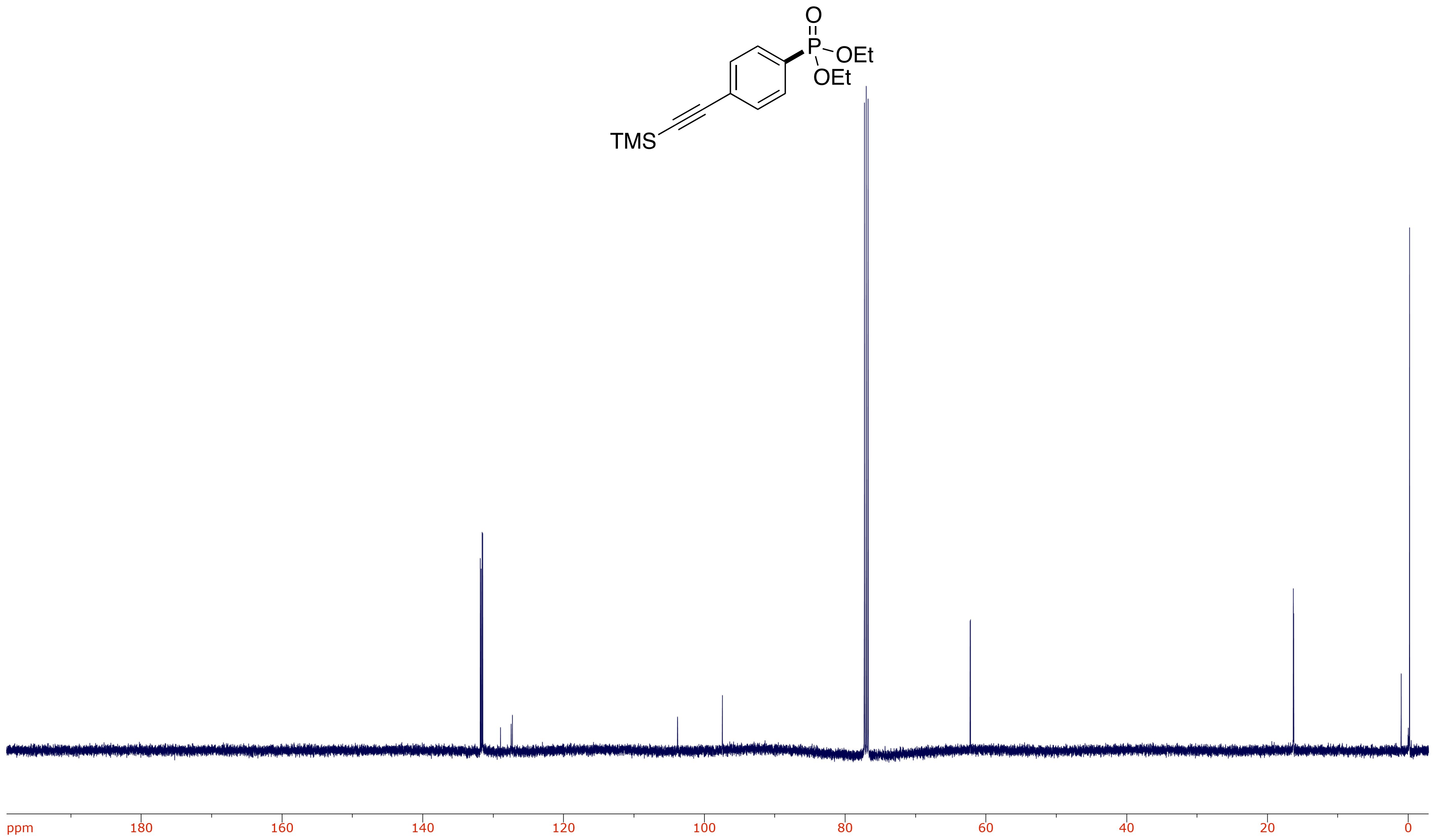

TMS
O
P
OEt
OEt
ppm
180
160
140
120
100
80
60
40
20
0

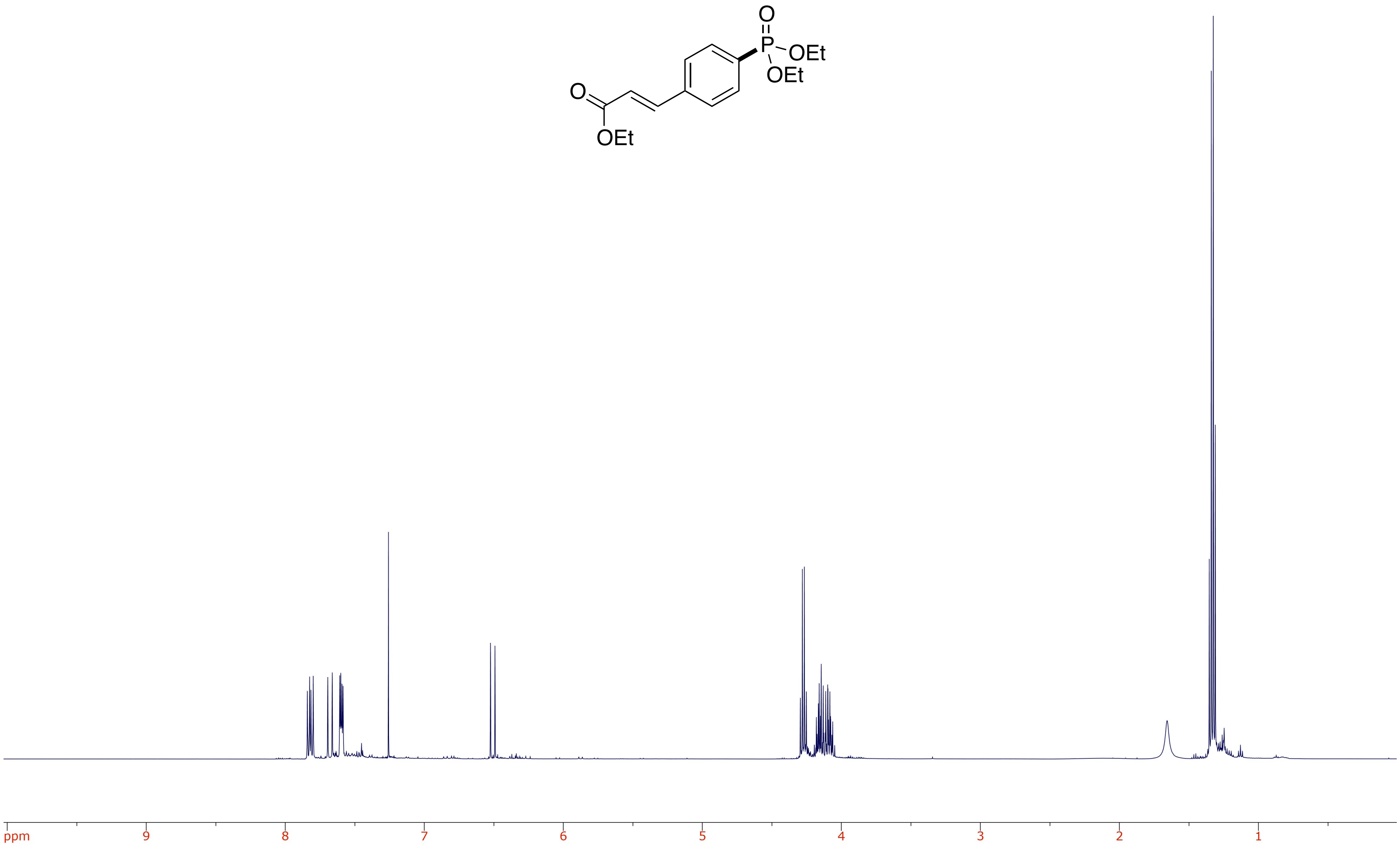
O
P
OEt
OEt
O
OEt
ppm
9
8
7
6
5
4
3
2
1

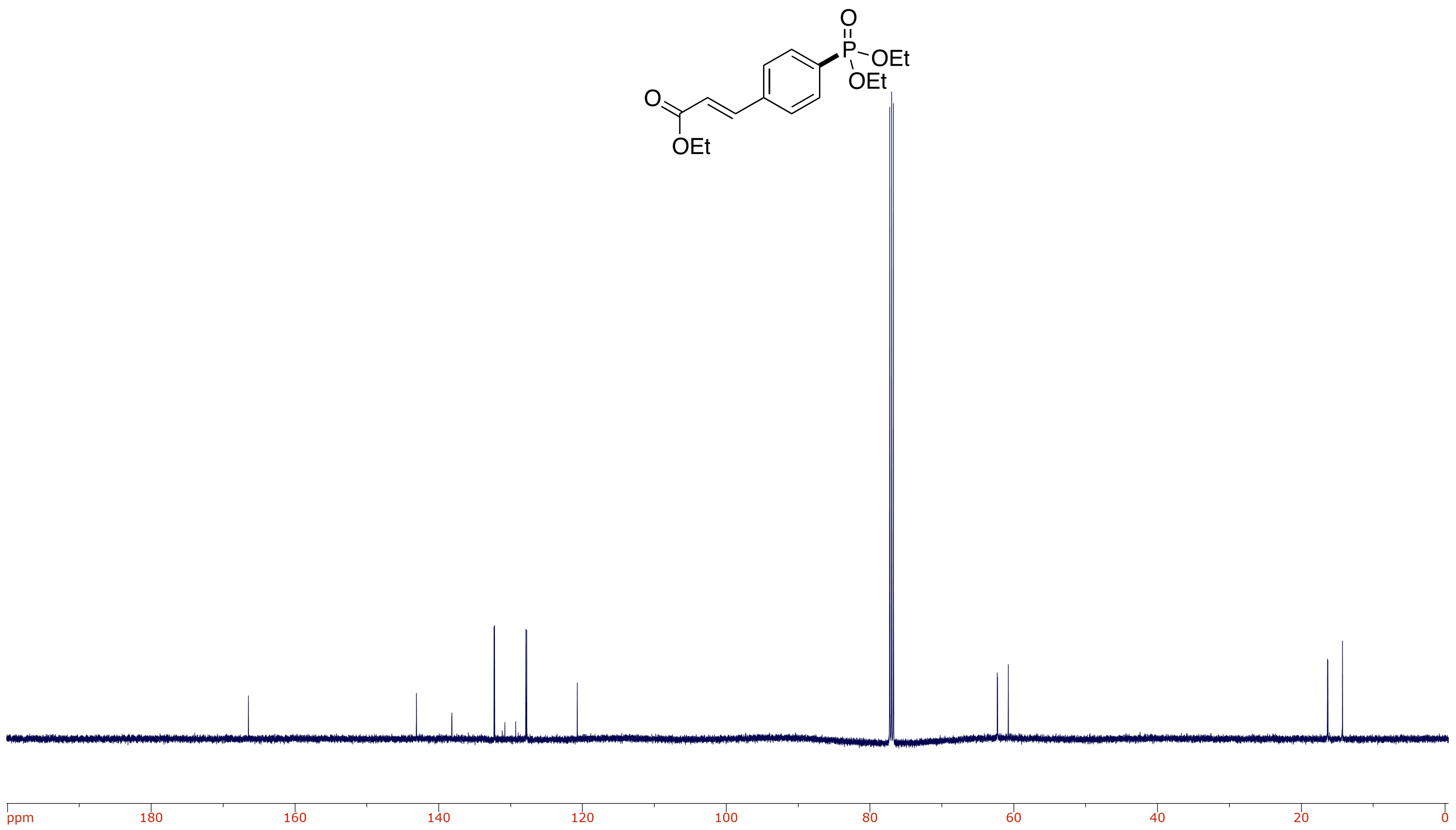
O
P
OEt
OEt
O
OEt
ppm
180
160
140
120
100
80
60
40
20
0

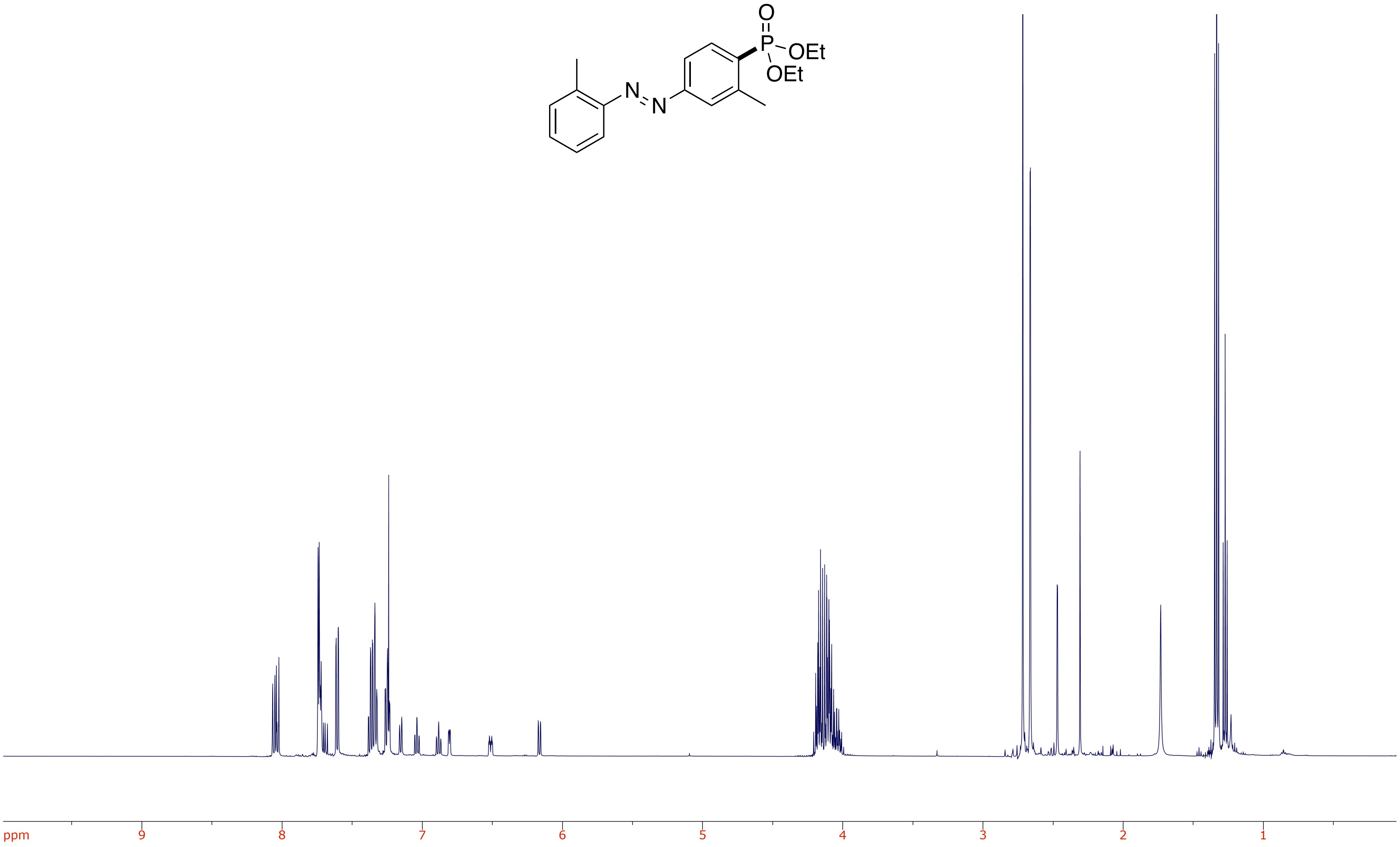

OEt
OEt
O
P
N
N
ppm
9
8
7
6
5
4
3
2
1

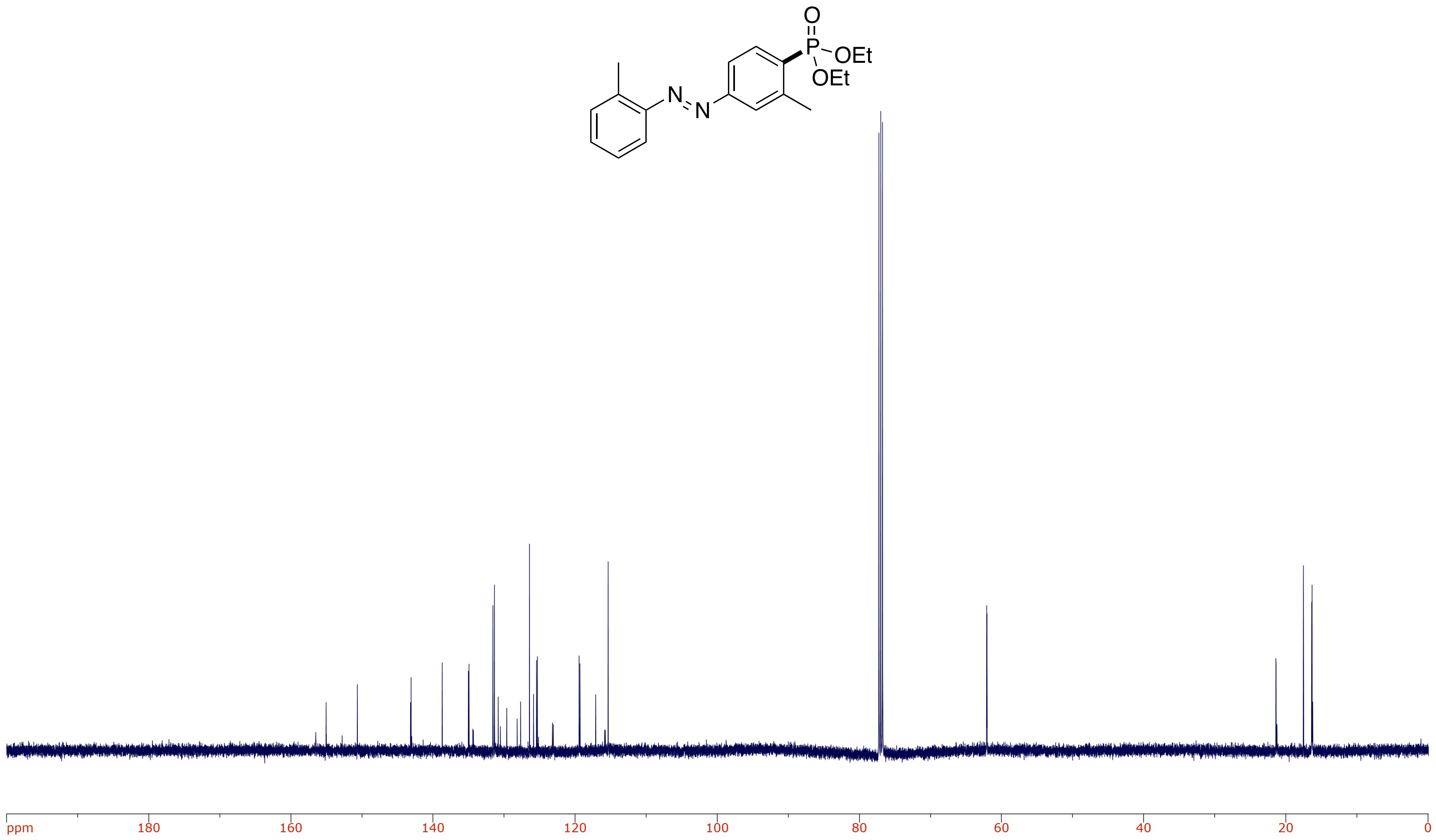
O
P
OEt
OEt
N
N
ppm
180
160
140
120
100
80
60
40
20
0
